# Use of Factorial Design for Calculation of Second Hyperpolarizabilities

**DOI:** 10.3390/nano15171302

**Published:** 2025-08-23

**Authors:** Igors Mihailovs, Ekaterina Belobrovko, Arturs Bundulis, Dmitry V. Bocharov, Eugene A. Kotomin, Martins Rutkis

**Affiliations:** 1 Institute of Solid State Physics, University of Latvia, 8 Ķengaraga St., LV-1063 Riga, Latvia; ekaterina.belobrovko@edu.rtu.lv (E.B.); arturs.bundulis@cfi.lu.lv (A.B.); dmitrijs.bocarovs@cfi.lu.lv (D.V.B.); martins.rutkis@cfi.lu.lv (M.R.); 2 Institute of Chemistry and Chemical Technology, Faculty of Natural Sciences and Technology, Riga Technical University, 3 Paula Valdena St., LV-1048 Riga, Latvia; 3 Institute of Applied Computer Systems, Faculty of Computer Science, Information Technology and Energy, Riga Technical University, 10 Zunda krastmala, LV-1048 Riga, Latvia; 4 Mechanical and Biomedical Engineering Institute, Faculty of Civil and Mechanical Engineering, Riga Technical University, 1 Paula Valdena St., LV-1048 Riga, Latvia; 5 Transport and Telecommunication Institute, 2 Lauvas St., LV-1003 Riga, Latvia

**Keywords:** nonlinear optics, second hyperpolarizability, full-factorial design, Sadlej-pVTZ basis set

## Abstract

There has been considerable scientific interest in third-order nonlinear optical materials for photonic applications. In particular, materials exhibiting a strong electronic optical Kerr effect serve as essential components in the ultrafast nonlinear photonic devices and are instrumental in the development of all-optical signal processing technologies. Therefore, the accurate prediction of material-relevant properties, such as second hyperpolarizabilities, remains a key topic in the search for efficient photonic materials. However, the field standards in quantum chemical computation are still inconsistent, as studies often lack a firm statistical foundation. This work presents a comprehensive in silico investigation based on multiple full-factorial experiments, aiming to clarify the strengths and limitations of various computational approaches. Our results indicate that the coupled-cluster approach at the CCSD level in its current response-equation implementations is not yet able to outperform the range-separated hybrid density functionals, such as LC-BLYP(0.33). The exceptional performance of the specifically tailored basis set Sadlej-pVTZ is also described. Not only was the presence of diffuse functions found to be mandatory, but also adding ample polarization functions is shown to be inefficient resource-wise. HF/Sadlej-pVTZ is proven to be reliable enough to use in molecular screening. Meta functionals are confirmed to produce poorly consistent results, and specific guidelines for constructing range-separated functionals for polarizability calculations are drawn out. Additionally, it was shown that many of the contemporary solvation models exhibit significant limitations in accurately capturing nonlinear optical properties. Therefore, further refinement in the current methods is pending. This extends to the statistical description as well: the mean absolute deviation descriptor is found to be deficient in rating various computational methods and should rather be replaced with the parameters of the linear correlation (the slope, the intercept, and the R^2^).

## 1. Introduction

In recent years, there has been considerable scientific interest in third-order nonlinear optical materials for photonic applications such as soliton microresonators, all-optical switches, and photon-based quantum computing, along with optical power limiting and data storage [1,2,3,4,5,6,7,8,9,10] as well as in other areas including remote sensing and [bio]chemical analysis [11,12]. In particular, materials demonstrating a pronounced electronic optical Kerr effect are a key component in devices for ultrafast data processing [13]. Among these, molecular materials have garnered great attention due to their fast response times and great potential for modification and tailoring [3,4,14]. Presently, it has become a common practice to precede experimental studies with a quantum chemical assessment of provisionally selected materials. This not only helps reduce the overall cost of the research, but also broadens its scope by greatly improving the efficiency of screening of a wide range of candidate materials [15].

Despite numerous methodological studies in the field [5,9,16,17,18,19,20,21,22,23,24,25,26,27,28] there still is no widely accepted approach to calculations of the electronic Kerr effect of molecular materials. In particular, studies that go beyond a narrow selection of density functionals and rely on more informative descriptors than the mean absolute deviation (MAD) remain scarce. Moreover, reference data used for benchmarking can be subject to uncertainties, which may not be immediately apparent to researchers without an experimental background. One of the main issues in the experimental characterization of optical Kerr response is the proper separation of various contributions to the intensity-dependent refractive index. While it is widely accepted that using lasers with a pulse width of <100 ps gives a correct estimation of the optical Kerr effect (OKE) [29,30], the measured value, alongside the electronic OKE contribution, incorporates multiple other contributions, the most dominant being the reorientation contribution [31]. To accurately evaluate the pertinence of a specific material for high-bandwidth all-optical applications, the electronic Kerr effect must be isolated from the slower contributions. The experimental separation of such contributions is not trivial and requires either a laser source with much narrower pulse widths than was mentioned above—of <200 fs, or polarization-resolved measurements [32]. This becomes very relevant when assessing the performance of computational methods, as having access to a reliable and well-characterized set of reference data is crucial. This study aims to address this need. We use reference experimental data from reference [31], which is one of the most extensive experimental studies where authors have presented separated Kerr contribution values for various simple compounds in the liquid state. Also, we tested various figures of merit for the correspondence between the experimental and calculated data, particularly correlation-related ones. Furthermore, we employed the full-factorial experiment design (in silico) to ensure that the comparison is statistically adequate. Finally, to evaluate the performance of methods, we used both frequency analysis with contingency tables (to assess the performance of specific computational parameters) and normalized contrast analysis (to assess the systematic impact of certain features common to multiple models).

## 2. State of the Art and Challenges in Second Hyperpolarizability Calculations

When a molecule is placed in an external electric field, its electronic system polarizes itself. The ability of the electronic system of the molecule to be polarized is described by its polarizabilities. For an accurate description of this polarization, a Taylor-series expansion of the polarizabilities is used, which in terms of energies is:

(1) $$E=E_{0}+{\vec{\mu }}_{0}\;\cdot \;{\vec{F}}_{a}+\alpha \;\cdot \;{\vec{F}}_{a}\;\cdot \;{\vec{F}}_{b}+\beta \;\cdot \;{\vec{F}}_{a}\;\cdot \;{\vec{F}}_{b}\;\cdot \;{\vec{F}}_{c}+\gamma \;\cdot \;{\vec{F}}_{a}\;\cdot \;{\vec{F}}_{b}\;\cdot \;{\vec{F}}_{c}\;\cdot \;{\vec{F}}_{d}+\ldots$$

The quantity $\mu_{0}$ is the static dipole moment (a vector), α is the linear polarizability (a Rank 2 tensor), β is the first hyperpolarizability (a Rank 3 tensor), the γ is the second hyperpolarizability (a Rank 4 tensor), and so forth. Each new order of the polarizability series allows for a more precise description of the electronic processes under the influence of one or multiple electric fields (some of which may be electromagnetic radiation, including light). The names “linear”, “first”, “second”, and so forth originate from the expansion of the generalized dipole moment, which describes the linear polarization of the molecule:

(2) $$\vec{\mu }={\vec{\mu }}_{0}+\alpha \;\cdot \;{\vec{F}}_{a}+\beta \;\cdot \;{\vec{F}}_{a}\;\cdot \;{\vec{F}}_{b}+\gamma \;\cdot \;{\vec{F}}_{a}\;\cdot \;{\vec{F}}_{b}\;\cdot \;{\vec{F}}_{c}+\ldots$$

Then α provides a linear correction to the generalized linear polarization, β accounts for the second-order correction, and γ—for the third-order correction. Therefore, the third-order nonlinear activity of a molecule is described by its second hyperpolarizability γ.

There are three main ways to compute hyperpolarizabilities, which are all related in some way to the perturbation expansion of the Hamiltonian in the presence of an external electric field (which is possibly time- or frequency-dependent), as well as to the response function theory. One is the indirect way, the coupled-perturbed self-consistent field (CPSCF, or usually CPHF/CPKS) [33,34,35,36,37,38,39,40,41]. There are also two direct approaches. The first direct approach is the analytical solution of the response equations (RE) in one or another form [42,43,44,45,46,47,48,49,50,51]. The second direct approach is the reconstruction of the response functions via the sum-over-states formalism (SOS), usually used in a truncated form [42,45,52].

Equally often, computational codes use the response equation and CPHF/CPKS formalism; however, it is not always implemented to the third order, usually stopping at the second-order property of β. The key reason for such a situation is the consequences of the so-called Wigner’s (2n+1) rule. According to this rule, it is possible to obtain the (2n+1)-th order corrections to the energy using the *n*-th order variational corrections. It follows therefore that the third correction to the energy in its Taylor series ($\beta \;\cdot \;F^{3}$; see Equation (1)), can be obtained from the first-order perturbation, which uses the μ operator) [34,44]. Consequently, the first hyperpolarizability is obtained with a rather slight effort in comparison to the higher-order quantities.

This limitation is sometimes circumvented by a numerical differentiation of the analytically obtained first hyperpolarizability β for the applied electric field. The numerical differentiation to obtain polarizabilities in general is called the finite-field (FF) approach. Among the four most widely used (see Supplementary Information) quantum chemistry (QC) packages in the field of optical nonlinearities, two use this approach to compute the third-order correction (i.e., the γ). These software suites are Gaussian [53,54] (uses CPSCF+FF) and ADF [55,56] (uses RE+FF). In contrast, the second and third most widely used QC codes in the field, Dalton [57,58] and GAMESS [59], support the computation of a fully analytical second hyperpolarizability. This is a significant advantage because the finite-field approach cannot account for the frequency dependence. The reason for this is that, by the definition of the FF approach, the applied field is held constant during the calculation, which is exactly why it is easier to implement such an approach. However, certain experimental properties require a certain symmetry of frequencies in the definition of the second hyperpolarizability, e.g., the measurement of optical Kerr effect (OKE), also known as the intensity-dependent-refraction-index (IDRI) measurement, has the frequency dependence of γ(−ω;ω,−ω,ω), which cannot be provided by the CPKS+FF approach. Yet this is the experiment from which the reference value for the present study comes. The notation γ(−ω;ω,−ω,ω) means that there are three incoming frequencies (after the semicolon) and one outgoing frequency (before the semicolon), which must be the sum of the incoming frequencies.

The characteristics of the methodologies are compiled in Table 1.

Besides the methodology, other computational parameters influence the results. Section S1 in the Supplementary Information shall provide the reader with a miniature review of the current state of the art in this respect. As studies on computing γ are not too numerous, we also—with caution—included studies discussing these “parameters” for β calculations. This should not hamper the analysis because there is a great deal of correlation between these quantities. Here, we must quickly summarize its key points.

Sometimes, not just the methodology of computing the hyperpolarizability matters, but also whether the frequency dependence is included in the model [16,60]; sometimes, the correlation between the static (zero-frequency) and the dynamic calculations is not good enough for these to be used interchangeably [17,61]. The environment effects can be pronounced [22,62,63,64], both the direct and the indirect ones [65,66,67,68] (the first group of effects describes the direct impact of the solvent reaction field on the polarization of the molecule; the second corresponds to changes in hyperpolarizability due to changes in the geometry of the molecule in the presence of a solvent). The Hamiltonian used for the geometry optimization is not considered too important for relatively small molecules [69,70], which constitute the set we used for the present study. On the other hand, for molecules which can form hydrogen bonds, it is well-known that the hyperpolarizabilities can be non-additive when increasing the number of molecules, and this non-additivity grows when so does the order of polarizability [71,72]. The ability to describe hydrogen bonding well varies noticeably among Hamiltonians [73,74]. In terms of the Hamiltonian used for the calculation of hyperpolarizability itself, the recommended methods are typically the Møller–Plessett perturbation theory methods (such as MP2) [16,25,75,76,77,78,79], the range-separated hybrid density functionals [18,20,24,25,64,76,80,81,82,83,84,85] and sometimes the meta density functionals [24,64,86] or even the Hartree–Fock method [4,16,17,27,87,88,89,90,91,92,93,94]. The composition of a range-separated functional can play an important role [19,21,22,80,83,95], and the same is true for the Møller–Plessett methods [68] and even for the coupled-cluster methods [20,77,96,97,98], which typically require a certain degree of sophistication.

Regarding the basis sets, their required composition for hyperpolarizability calculations typically features at least some diffuse [9,17,28,77,99] and polarization [17,27,76,99] functions, but performance can vary strongly between basis set families, and it is especially important not to be fooled by the similarity in the names and to look straight to their actual composition [5,61]. Some basis sets are specifically assembled and optimized for the calculations of polarizabilities. This property-tailoring allows making these basis sets relatively compact. Such basis sets are considered very promising [61,100,101,102,103], especially the basis set by Sadlej et al. [104,105].

With all that said, it is clear that there is still no consensus on the optimal computational method of second hyperpolarizability. Therefore, we opted for a thorough study, comparing the performance of some of the most popular methods for small organic molecules (solvents) with the purely electronic second hyperpolarizabilities experimentally measured via studies of the optical Kerr effect.

## 3. Materials Studied

For present study we selected 22 organic solvents which have been experimentally studied by Zhao et al. in 2018 [31]: (a) aromatic and heteroaromatic compounds (benzene, toluene, p-xylene, pyridine, o-dichlorobenzene, nitrobenzene); (b) partially unsaturated compounds (acetone, acetonitrile, DMSO, DMFA, carbon disulfide CS_2_); (c) chlorinated hydrocarbons (chloroform, dichloromethane, tetrachloromethane); (d) polar saturated compounds (water, methanol, ethanol, 1-buthanol, 1-octanol, tetrahydrofuran); (e) non-polar saturated compounds (*n*-hexane, cyclohexane). Solvents are a suitable choice, as many experiments on third-order nonlinearity are conducted in solution, and the hyperpolarizability of the solvent must therefore also be taken into account. Moreover, the choice of small (but not too small) molecules allows us, in a reasonable time, to perform a study that is more or less comprehensive in terms of methods covered. It was expected that there could be complications with describing the experimental values for the water and the alcohols due to the hydrogen bond network formation, and because the experimental values for the water and the lowest alcohols were below the error level. This was addressed by creating clusters of molecules and observing how the second hyperpolarizability per monomer changes with the size of a cluster. See the Supplementary Information for all optimized chemical structures used in this study. Experimental values are of pure third-order electronic second hyperpolarizability, free of thermal contributions due to the use of femtosecond pulses and separated from any collisional, librational, vibrational, and reorientational contributions; this makes Zhao et al.’s [31] results exceptionally useful for comparisons with the results of theoretical computations.

## 4. Computational Methods

This computational study considers multiple aspects of quantum chemical computations at the same time, using a full-factorial design for subgroups of these aspects (see below) [106]. In the language of experiment design theory, these “aspects” or “parameters” of the calculation are named *factors*, each with two or more *statistical levels* (options, or possibilities). The brief description of the factors is given below; the more detailed description is given in the Supporting Information.

1. Computational methodology (one statistical level: coupled-perturbed Hartree–Fock or Kohn–Sham augmented with the finite field for the third dimension (CPHF+FF or CPKS+FF); another statistical level: response-equation formalism—RE);
2. two-electron (exchange and correlation) Hamiltonian: up to 18 statistical levels in total—(a) wave-function-based methods: HF [107], CC2 [108], CCSD [109]; (b) global-hybrid (GH) density functionals: B3LYP [110,111] ($\alpha_{HF}$ = 0.2), BH&HLYP [112] ($\alpha_{HF}$ = 0.2); $\alpha_{HF}$ here is the contribution of HF-like exchange; (c) τ-meta-global-hybrid (MGH) density functionals: M06 [113] ($\alpha_{HF}$ = 0.27), M06-2X [113] ($\alpha_{HF}$ = 0.54) [114], MN15 [115] ($\alpha_{HF}$ = 0.44); (d) eight range-separated-hybrid (RSH) density functionals, based on LC-BLYP [116] and CAM-B3LYP [117], scheme, with systematic variation in α, β, ω parameters (see the Supporting Information); (e) a τ-meta-range-separated-hybrid density functional M11 [118] (ω = 0.25, α = 0.43 and β = 1.00);
3. basis sets—up to 8 statistical levels (Pople sets 6-311G(d,p), 6-311+G(d,p), 6-311++G(d,p) and 6-311++G(3df,3pd); Ahlrichs basis sets aug-cc-pVDZ and aug-cc-pVTZ; tailored basis sets Sadlej-pVTZ [104,105] and aNLO-V [27]);
4. whether the solvation is included in the model (two statistical levels, vacuum and solvated: C-PCM [119,120] in Gaussian and PELib [121,122] in Dalton);
5. type of solvation model in Gaussian (three statistical levels, C-PCM, IEF-PCM [123] and SMD [124]; the latter is IEF-PCM extended with non-electrostatic terms);
6. integration grid for DFT methods, tested separately for Gaussian and Dalton because of incomplete compatibility between software suites; the differences are explained in Section 2 “The details of the quantum chemical calculations performed”, paragraphs *g* and *h*, in Supplementary Information.
7. static or dynamic calculation of the second hyperpolarizability (two statistical levels)—available only for Dalton calculations; this is explained in Section 2 “The details of the quantum chemical calculations performed”, paragraph *i*, in the Supplementary Information.

We should mention separately the method used for geometry optimizations (five statistical levels of Hamiltonian, two statistical levels of whether the C-PCM description of the medium is included in the model). This part of the study did not cover all molecules, and it was decided that the best combination of statistical levels is ωB97XD/6-311G(d,p)/C-PCM; see the description and the further discussion in Supplementary Information. Throughout the rest of the study, we used geometries computed with this method. Thus, it cannot be considered a fully fledged part of this study.

Calculations employing the CPKS+FF and CPHF+FF methods were performed using Gaussian 09, revision D.01 [125] (for points calculated with FineGrid) and Gaussian 16, revision C.01 [126] (for points using UltraFineGrid), as previously described. For response-equation-based computations, Dalton 2020.0.1 [117] was utilized. Visualization and geometry-related tasks were carried out using Avogadro versions 1.2.0 [127,128] and 1.93.0 [127,129], as well as UCSF Chimera version 1.15 [130,131].

As computational run time was also compared for a selected subset of calculations, it is important to note that all hyperpolarizability computations within this subset were performed on nodes of the Latvian SuperCluster (LaSC), equipped with 40-core Intel(R) Xeon(R) Gold 6148 CPUs (2.40 GHz), 192 GB RAM, and SSD scratch storage. Outside of this subset, other nodes in the same cluster were occasionally used. To provide a realistic and practical context, we report parallel (rather than serial) computation times. We explicitly acknowledge that different methods vary in their degree of parallel implementation; in particular, Dalton 2020.0.1 does not support parallel computation of CCSD(fc) hyperpolarizabilities.

For statistical analysis and data presentation, we used the R programming language (version 4.3.3, 2024-02-29) [132], employing the following packages: *graphics*, *mblm*, *car* [133], *rcompanion* [134], *nnet* [135], *data.table* [136], and *wordcloud* [137].

## 5. Design and Statistics: Comparison Between the Calculated and the Experimental Data

### 5.1. Choice of Quality Descriptors for Modeling

To describe the relative quality of computational “parameters”, we need to select certain quality descriptors. Traditionally, the mean unsigned error (MUE), or the mean absolute deviation (MAD) of calculated values from the experimental ones, is used for this purpose. However, the most reliable result of a series of quantum chemical calculations is a trend, not the absolute value [17]. Moreover, a statistically sound comparison between two continuous-value datasets is a model of correlation. As we expect a one-to-one correspondence between the calculated and the experimental data, a linear correlation model was selected. The adjusted coefficient of correlation (adj. $R^{2}$) describes the goodness of fit, whereas the slope and the intercept describe how well the calculated values mirror the experimental ones. The best prediction quality of a computational model is when the intercept nears zero and the slope and the correlation coefficient nears one; to assess this quality, we defined the descriptor for the correlation coefficient quality as $1-R^{2}$ and for the slope quality to be:

(3) $$b^{\prime }=1-\frac{1}{b}$$

where *b* is the slope.

MAD, in fact, is a container of the slope, the intercept, and the correlation coefficient, related to the undivided sum of squares. We also employed an alternative average descriptor, the average deviation from the desired value for every descriptor, as stated above. This descriptor allows us to assess the overall correlation quality on the assumption that everyone of the three correlation descriptors (and MAD) is equally important. Naturally, all the data were centered and normed before the calculation of average, using, respectively, the median and the standard deviation of the subset of the best 1/10 of all results for each descriptor (to avoid the bias by any outliers and different width of deviation distribution, which is especially evident when comparing the slope and the adjusted $R^{2}$). In addition, we decided to test the average without the slope, as a method that yields a good correlation without a good slope is better than a method that yields a good slope but a bad correlation.

Overall, this study considers six quality descriptors for the computed data: the slope, the intercept, the adjusted $R^{2}$ (from the correlation with the experimental data), the MAD, the average of the deviation from the ideal for the four previously mentioned descriptors, and the same average excluding the slope.

### 5.2. Factorial Design of the Study

It is necessary to discuss the design of this study from the viewpoint of statistics and the experiment design theory. It is important to highlight that this in silico study does not adopt the traditional approach to experiment design, where certain parameters are fixed while one or two others are varied. Although this methodology is widespread in computational chemistry, it lacks robustness: techniques that perform well under specific parameter combinations may fail under others, often due to accidental or adverse error compensation. To overcome this limitation, we employed a full-factorial design (FFD), in which subsets of factors and their statistical levels are selected, and all possible combinations of these levels are used to define a model chemistry - each such combination is referred to as a *treatment*. This approach allows for a statistically sound comparison between factor levels, as for every specific level (every functional, every basis set, etc.), there are many data points with different levels in other factors [106]. This ensures we are not selecting a particularly lucky combination of statistical levels but the levels (Hamiltonians, basis sets, etc.) that tend to provide optimal results. To ensure the quality of the results, every statistical level of a factor occurs an equal number of times within each dataset, thus the in silico experiment design is balanced.

As it is virtually impossible and even unnecessary to calculate every combination of every factor (sometimes the calculation cannot converge, or we are wasting too much time checking for a specific factor), we divided our in silico experiment space into multiple subspaces, or data subsets, 19 in total. These datasets (their composition and normality) are described in the Supplementary Information.

### 5.3. Statistical Analysis of the Results

In terms of data analysis, we used two ways to look at the data: performance of specific levels of the factors or performance of a specific trait among the statistical levels (see below). To test the performance of specific statistical levels (basis sets, etc.), we sorted data points for each descriptor separately (each point corresponds to a specific combination of the factor levels, for example, B3LYP/aug-cc-pVDZ/vacuum would be one point). Then we take the top 10% of the sorted dataset and count the number of occurrences for every statistical level (e.g., for an experimental set of 88 combinations, LC-BLYP was used in 2 of the top 8 combinations sorted by the absolute value of the intercept). This comparison tells us what factor levels tend to yield the best results for a specific descriptor (intercept in the previous example). The same process was also repeated for the bottom 10% of the sorted datasets; this comparison tells us what factor levels tend to yield the worst results. In fact, what we obtain is a standard contingency table, on which $\chi^{2}$-based comparisons can be performed. The $\chi^{2}$-test, however, did not produce any meaningful results in our case, possibly because of too small a number of cases in the 10% datasets, and Fisher’s exact test is rather too conservative. That is why we used just the ratio of actual counts in the top 10% bin and the counts that would follow from the uniform distribution of the statistical levels. For datasets 1, 2, 4, 5, 9, and 10, the ratio was then also divided by the $\chi^{2}$-distribution quantile, appropriate for the size of the sample. This measure allows for an increase in the discriminating power of the analysis, but only if the number of statistical levels of the factor in question is high enough. As for datasets 3, 6, 7, 8, and 11–19, dividing by the quantile actually diminished the discriminating power for the third factor of the in silico experiment, so we resorted to not applying such a normalization for these data subsets. All results from these distribution comparisons were visualized as a heat map, giving us 38 heat maps for 19 subsets of data.

To evaluate the performance of specific traits, we calculated linear contrasts [106]. These are linear combinations of data built to reflect specific differences across a dataset, to evaluate systematic differences between data points. The definition of linear contrasts is more thoroughly described in the Supplementary Information.

We visualized the *p*-values for different contrasts and descriptors as a heat map, making it three heat maps per a subset of data and 57 heat maps in total for this study (not counting the maps associated with the calculations of the geometry optimization method influence). Owing to space constraints and clarity considerations, only a representative selection of these heat maps is presented in the main text, while the extended set is provided in the Supporting Information.

## 6. Results and Discussion

### 6.1. Examination of Scatter Plots

**Figure 1:**
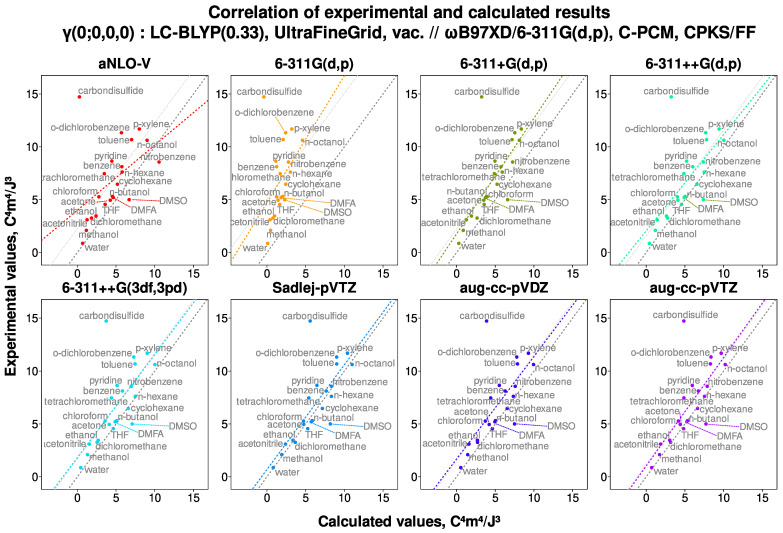
An example of correlation between the experimental and the calculated values for the static second hyperpolarizability γ(0; 0, 0, 0), computed with the CPKS+FF formalism and the LC-BLYP(0.33) density functional. The colored line shows the linear approximation; the dashed grey line is the 1–1 diagonal, and the dotted grey line is the diagonal shifted so as to go through the center of mass of the data points. Here, the point for CS_2_ is an obvious outlier; fully saturated compounds tend to lie very close to the diagonal, but the larger the electron delocalization, the more the calculated value is underestimated.

**Figure 2:**
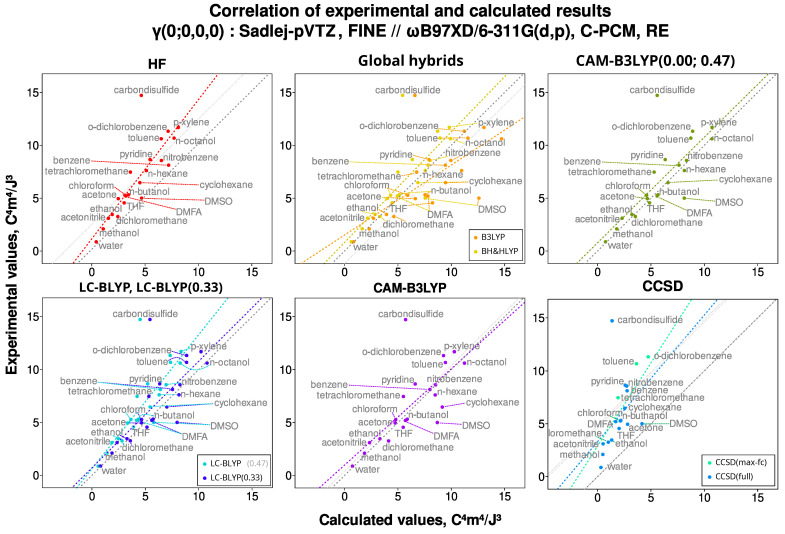
An example of correlation between the experimental and the calculated values for the static second hyperpolarizability γ(0; 0, 0, 0), computed with the RE formalism and the Sadlej-pVTZ basis set. The colored line shows the linear approximation; the dashed grey line is the 1–1 diagonal, and the dotted grey line is the diagonal shifted to go through the center of mass of the data points. Here, we can see that not for every Hamiltonian are the points for the fully saturated compounds closest to the diagonal, and how far from the diagonal CCSD results are.

We first examine the scatter plots, which serve to visually evaluate the degree of correlation between the calculated and the experimental values. As representative examples, two sets of plots are presented: the first (Figure 1) corresponds to CPKS+FF calculations performed with different basis sets, while the second (Figure 2) illustrates the outcomes of RE calculations employing various exchange–correlation Hamiltonians.

First, the most striking feature of the correlation plots is the outlier point for carbon disulfide, CS_2_. It is present in all the calculated correlations, not just ones shown in Figure 1 and Figure 2. Regardless of treatment, the γ value for CS_2_ seems to be either computationally underestimated or experimentally overestimated, in any case severely. Also, in many cases, the point for DMSO is borderline to be an outlier. This contrasts with the CS_2_ case, though, because the calculated result is overestimated with respect to the experimental one. Notably, these two are the only molecules that contain sulfur, and in both cases, the sulfur participates in double bond formation. Other molecules containing elements from the third period of the periodic table (chlorine) are not misbehaving in this fashion, except for slightly larger deviations for tetrachloromethane. However, the chlorine only participates in single bonds in any of them.

There could be multiple explanations for these peculiarities. One is that for carbon disulfide, there is a large resonance contribution to the experimental value of the second hyperpolarizability [138,139]. Indeed, CS_2_ has a one-photon transition around 337 nm (See the Supplementary Information), which is symmetry-forbidden. The rule is lifted in reality due to finite-temperature vibrations, causing experimentally observed non-negligible one-photon molar absorbance of ca. 0.025 L/(mol·cm) around 400 nm [140,141]. On the other hand, nitrobenzene absorbs much more intensively at around 400 nm, about L/(mol·cm) [142,143], which corresponds to the calculated transition at 313 nm. However, the nitrobenzene point is not an outlier in any scatter plot.

Our previous experimental studies, however, revealed that the γ spectrum of CS_2_ has prominent nonlinear absorption around 800 nm. The phenomenon was explained not by the γ-related third-order effects but by the fifth-order effects and three-photon resonance. It persists up until 1190 nm and can significantly influence the measured results in the spectral region around 800 nm [144]. This study was conducted in the femtosecond range and is therefore also free from the reorientation contributions, as are the data for Zhao et al. [31] we use for the experimental reference in the present study. These findings about the fifth-order effects are in concordance with other groups [145,146]. The fifth-order effects are present only for intense laser beams (over 75 GW/cm^2^) [145], but it is hard to deduce the precise intensity used by Zhao et al. [31]. Nevertheless, the presence of fifth-order contributions provides a plausible explanation for the anomalous behavior observed in the case of CS_2_.

The discrepancy observed for DMSO remains unresolved, as the experimental value is lower than the calculated one. Given that the study by Zhao et al. [31] employed femtosecond laser pulses, it is difficult to determine the exact origin of this deviation. Further experimental investigations are necessary to clarify this inconsistency. In light of these considerations, we excluded the CS_2_ data from subsequent analysis, since it is a clear outlier, likely due to dynamic effects not captured by most of our computational methods, while retaining the DMSO data, as it does not unambiguously exhibit outlier behavior.

Reflecting on the other visually detectable results, it can be noticed that the points associated with the saturated molecules lie very close to the 1–1 diagonal for most of the methods. When a double or a triple bond appears, the calculated results are slightly underestimated, and this trend becomes more intense for the aromatic molecules. This is true for both the static and the dynamic calculations. The only difference is that, for the static ones, the value of γ is underestimated, and for the dynamic ones, the value of γ is overestimated—the more heavily the longer is the π conjugation path. Unfortunately, this persists even for the coupled-cluster case, showing the limits of contemporary quantum chemical description of electronic polarizabilities. Interestingly, chlorinated methanes also depart from the diagonal, especially the CCl_4_ molecule, signifying the presence of delocalized states due to the overlap of neighboring halogen atoms.

Moreover, it can be seen that the τ-meta density functionals are extremely susceptible to the choice of the basis set and produce desperately poor correlation quality (especially for the slope descriptor). Specifically, the γ values for seemingly random molecules (e.g., benzene but not toluene, or the lower alcohol molecules) were either dramatically underestimated or overestimated with meta functionals. Overall, the quality of the correlation with these Hamiltonians was, in general, uncharacteristically low for the present study. This happened for any basis set but the two Dunning’s sets (aug-cc-pVDZ and aug-cc-pVTZ). The reason for this is probably the comparatively much higher number of diffuse functions in these basis sets, and not the basis set design/family, as the researchers at the Minnesota University use Pople-style basis sets for their calculations [147].

One last thing to note is the effect of the solvation model’s presence in the Hamiltonian. From the scatter plots (see the Supplementary Information), it can be seen that the absolute values of γ increase when the solvation model is added. Unfortunately, this usually means that both the slope and the adjusted $R^{2}$ diminish (the points spread out from the trend), which is true for both the PELib and (noticeably stronger) for C-PCM. Even the cancellation of errors does not occur, as the change in values due to the solvation model is usually much stronger than the discrepancy that existed previously. More examples of the correlation scatter plots are available in Supplementary Information.

### 6.2. Analysis of Factor Influence

#### 6.2.1. Hamiltonians and Basis Sets

We begin the analysis from Data Subsets 1 (in combination with Subsets 4 and 10) and Subset 5 (in combination with Subset 9), representing the in silico experiments with the highest statistical levels of Hamiltonians. The reason for investigating the combinations rather than the results for just a single subset is the complementary nature of these data subsets. For example, Subset 1 contains results for CC methods but fewer basis sets (due to lower computational affordability), which limits the number of data points and probably distorts the results. On the other hand, the analysis with more basis sets does not include all the exchange–correlation Hamiltonians.

**Figure 3:**
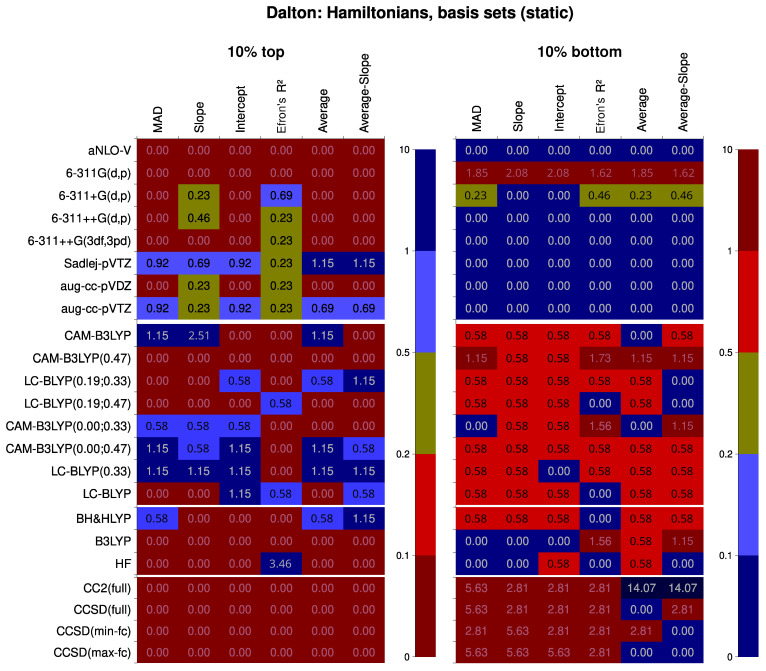
Performance of specific Hamiltonians and basis sets within the response-equation formalism, calculated as frequency thereof within the top 10% and bottom 10% bins of the Data Subsets 1, 4, and 10. The ratio is subsequently divided by the corresponding quantile of $\chi^{2}$ distribution for the normalization to the number of points. The closer the color is to dark red, the worse the performance; the closer the color is to dark blue, the better the performance. The numbers show the normed probability of a treatment to fall into the 10% or 10% worst bin; because of this, higher values are colored blue for the “10% best” pane but darkly red for the “10% worst” pane.

For the Subsets 1/4/10 (Hamiltonians and basis sets in Dalton, including CC), the performance of specific statistical levels of both factors is demonstrated in Figure 3. The only part that comes from the analysis of Subset 1 is the CC part; the rest agrees with Subsets 4 and 10 in general but not in details, and Subsets 4 and 10 agree fully with each other, so they were chosen to represent the trends.

First, we will make comments on the basis set performance. The arguably most robust turns out to be the compact but property-tailored Sadlej-pVTZ basis set. This basis set yields the best results for every descriptor but the $R^{2}$; also, it never produces particularly bad results. Close comes the aug-cc-pVTZ basis set. The worst-performing basis set is 6-311G(d,p), which has no diffuse functions. Peculiarly, the basis set highly packed with the polarization functions, 6-311++G(3df,3pd), is losing the competition even to 6-311++G(d,p).

Considering the Hamiltonians, the best-performing group is the range-separated hybrid density functionals, though only certain combinations of the three range-separation parameters produce valuable results. If we consider only the data Subset 1, the best among the RSH is LC-BLYP(0.19; 0.33). However, if we use Subsets 4 or 10, which both have more data points per Hamiltonian, this conclusion is rescinded: the most well-performing RSH turns out to be LC-BLYP(0.33). It was the second best for Subset 1. Tailing is CAM-B3LYP(0.00; 0.47), which is, again, true for all three subsets. The least well-performing is CAM-B3LYP(0.47) density functional.

Among the non-RSH SCF methods, BH&HLYP is nearing an average RSH in terms of performance, which is albeit not too good in comparison with LC-BLYP(0.33). Notably, B3LYP does not produce particularly good results, but also seldom produces exceptionally bad results either, except for $R^{2}$. Plain Hartree–Fock performs slightly better than it, though, especially for $R^{2}$, for which it performs decidedly better than any other Hamiltonian considered. These results are mostly in line with previous findings.

Interestingly, all the coupled-cluster methods perform worse than any other method, except that they typically do not give very bad results on average. This is an astounding result, as these methods are believed to represent the actual electron interaction to the finest extent available. The most probable cause is that the RE formalism is not yet well implemented for the coupled-cluster methods in Dalton. It is an undertaking task, as the algorithm of computation is significantly different from the SCF-based one. This is probably upheld by the observation that CC2 or frozen-core CCSD is performing slightly better than the full-electron CCSD, which might be due to some random fluctuations, a telltale sign of the algorithmic instability. The important practical conclusion, though, is that the CC should not be the method of choice if we are using RE as implemented in Dalton 2020. This should hopefully improve in future versions of this program. While this may seem to contradict the findings by other groups, these were either special cases or calculations with the finite-field approach, where there are no algorithmic hindrances.

Next, we should discuss the systematic traits of performance for different statistical levels of both factors (the Hamiltonian and the basis set). First, the results now bear striking differences between Subsets 1, 4, and 10. Significant systematic trends in the composition of a basis set are only found for Subset 1 (containing also the results with the CC methods). It follows that there is a greater dependence of the CC methods on the basis set than there is for density functionals. This manifests itself, in particular, in the need for ample diffuse functions to have a better value of intercept. The CC methods also have the most intense improvement of results if Sadlej-pVTZ basis set is used. This also results in more hits for the zeta factor (Sadlej-pVTZ is classified as double-zeta because some of the zeta shells are obviously diffuse).

**Figure 4:**
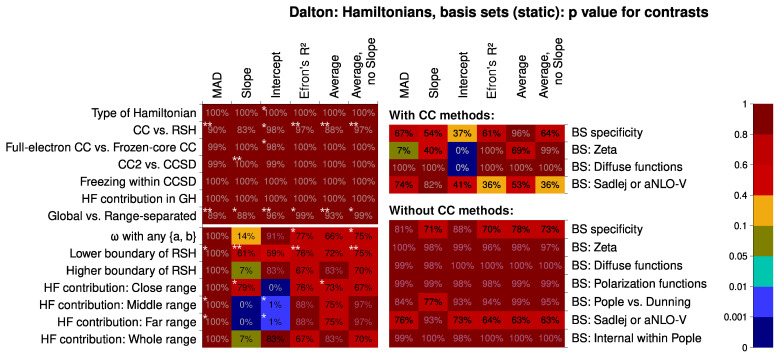
Statistical significance of certain linear contrasts for the different computational quality descriptors, Data Subsets 1, 4, and 10 (see the main article text for the clarifications). Significance is given by the *p* value of the F tests within the contrast-based analysis of variance (ANOVA). As the contrasts related to the basis sets bear much greater significance, for the reader’s comfort, we have marked the relative significance of the contrasts corresponding to the Hamiltonians. Specifically, contrasts making up more than 50% of the contrast mean squared deviation for the Hamiltonian contrasts alone, would be marked with three stars *** (but are not present); contrasts making up more than 20% are marked with two stars **; and contrasts making up more than 7.5% are marked with a single star *.

The performance of different Hamiltonians is, however, rather uniform if we only look at the results for the relatively broad Subset 1. There are only slight indications (marked with stars in Figure 4) that the RSH functionals are performing better than both the global hybrids and the CC methods as implemented in Dalton. In addition, CC2 is noted for its tendency to produce much lower slopes than CCSD. Also, the lower boundary of RSH (0% vs. 19% of HF-like exchange at the small inter-electron distances) is revealed to be somewhat important. The situation does not change much if we exclude the CC methods from the picture; then, almost no factors are deemed to be systematically important.

However, if we look at the dataset 10 only, with all the RSH functionals, we can notice specific traits there. First, the ω value is quite important for the slope, as well as the HF-like exchange proportion in the middle and the far inter-electronic range. To have a good intercept, the right HF proportion in the close range is also very important. By comparing with the classification of the RSH functionals (see the Supplementary Information), this analysis suggests that, on average, a lower percentage of HF-like exchange is favorable in the far and middle range, and an even lower percentage is beneficial in the close range. The reader should notice how these suggestions do not capture well the particularly successful statistical level, the LC-BLYP(0.33) functional. The other functionals do adhere to this trend, however, so this may be a useful design guide to construct even better range-separated hybrid functionals for calculations of the second hyperpolarizability. Two other significant conclusions can be drawn. First, the overall HF-like exchange contribution does not play a remarkable role in classifying the functionals. Second, the MAD descriptor is not discriminating at all, thus much less useful than the correlation descriptors (the slope, the intercept, and the $R^{2}$). This is a worrying finding, as many computational studies do employ exactly this descriptor to rate quantum chemical methods. The shortcomings of using MAD were previously highlighted by Pernot and Savin [148,149]. They proposed using bootstrap estimates for ranking various methods, a practice also greatly useful for single-factor comparisons. Probably, the practice of using MADs should be abandoned in further research. This lesser discriminatory power is much more evident in the contrasts and virtually absent from the distribution analysis, indicating the importance of analyzing the contrasts and trends.

**Figure 5:**
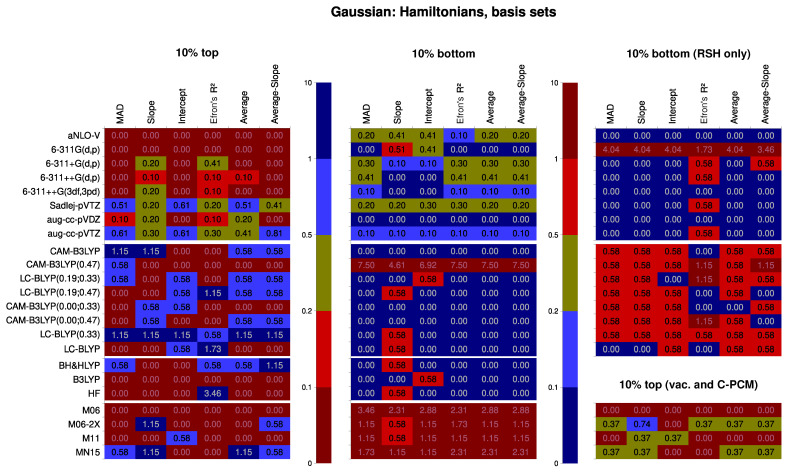
Performance of specific Hamiltonians and basis sets within coupled-perturbed Kohn–Sham formalism, calculated as frequency thereof within the top 10% and bottom % bins of the Data Subsets 5 and 9. The ratio is subsequently divided by the corresponding quantile of $\chi^{2}$ distribution for the normalization to the number of points. The closer the color is to dark red, the worse is performance; the closer the color is to dark blue, the better the performance. The numbers show the normed probability of a treatment to fall into the 10% or 10% worst bin; because of this, higher values are colored blue for the “10% best” pane but dark red for the “10% worst” pane.

For the coupled-perturbed SCF datasets (see Figure 5), the dominance of Sadlej-pVTZ is not as firm, save for the RSH-only dataset. Otherwise, it occupies the second place only, overtaken by the aug-cc-pVTZ basis set. Also, if we consider the broader range of density functionals, Sadlej-pVTZ considerably more frequently yields the results in the bottom 10% bin. Interestingly, with meta-hybrid functionals in the picture, the results of 6-311++G(3df,3pd) basis set are noticeably improved, at least in the bottom-10% bin. Together, these traits most probably signify the importance of an ample set of polarization (and diffuse) functions in the basis set for the meta functionals. This can also be seen from the inspection of particular combinations of the functional and the basis set. In the rare cases when the meta-hybrids are performing well, it is only with aug-cc-pVTZ, aug-cc-pVDZ, and occasionally with 6-311++G(3df,3pd).

Regarding the range-separated hybrid functionals, it is again confirmed that the CAM-B3LYP(0.47) functional is the worst RSH model within the present study. LC-BLYP(0.33), on the contrary, is again found to be the best RSH and overall the best-performing exchange–correlation Hamiltonian. Performance of BH&HLYP approaches that of a moderate RSH, while B3LYP does not perform well, just like for the response-equation methodology. Peculiarly, HF again yields good results in terms of data point dispersion around the trend line, absolutely dominating the top for this parameter. Among the meta-hybrid density functionals, only the non-separable range-separated MN15 produces considerably good results. Nevertheless, it suffers, along with the rest of meta functionals, from the tendency to yield particularly bad results if the basis set used lacks a sufficient amount of polarization and diffuse functions.

**Figure 6:**
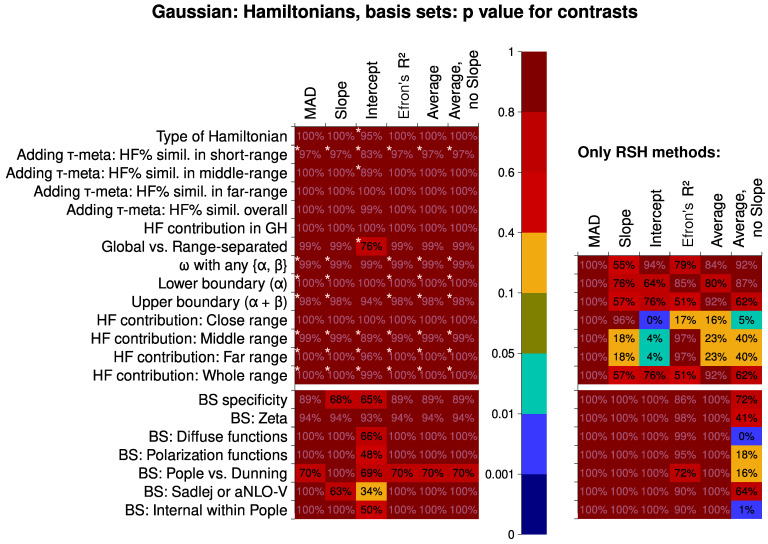
Statistical significance of certain linear contrasts for the different computational quality descriptors, Data Subsets 5 and 9 (see the main article text for the clarifications). Significance is given by the *p* value of the F tests within the contrast-based analysis of variance (ANOVA). As the contrasts related to the basis sets bear much greater significance, for the reader’s comfort, we have marked the relative significance of the contrasts corresponding to the Hamiltonians. Specifically, contrasts making up more than 50% of the contrast mean squared deviation for the Hamiltonian contrasts alone, are marked with three stars *** (but are not present); contrasts making up more than 20% are marked with two stars ** (not present either); and contrasts making up more than 7.5% are marked with a single star *.

Considering the contrasts (shown in Figure 6), they give very weak discriminatory power if the meta-hybrid functionals are still in the picture. Changing the contribution of the HF-like exchange is, again, producing more consistent change in the productivity than adding the meta term into the exchange–correlation Hamiltonian. The only exception is the close range (EXX contribution is then not so important). The ω and the boundaries of RSH are also somewhat consistently impacting the performance. The contrast of Pople vs. Dunning basis sets is the most prominent, as is expected, because of a relatively higher amount of polarization and diffuse functions (simultaneously) in the Dunning basis sets.

Intercept, just like for the response-equation data subsets, is the most sensitive descriptor.

The situation changes substantially, however, if we only look at the dataset with just the RSH density functionals. Hartree–Fock-like exchange contribution in the close range is now a very important contrast, less so the contribution in the middle and the far range. Similarly to what was seen before, the overall contribution of EXX is not discriminating much. The basis set contrasts are much less prominent in this case, indicating the high sensitivity of meta-hybrid functionals to the composition of the basis set. The average of intercept, $R^{2}$, and the MAD is, surprisingly, having a great discriminatory power. The findings, however, seem to be rather generic. The fact that the separate descriptors (and especially the average with the slope) did not yield any discrimination leads us to the conclusion that the use of this average descriptor does not have a practical advantage. Together with it, the intercept still has the strongest discriminating power among the descriptors.

**Figure 7:**
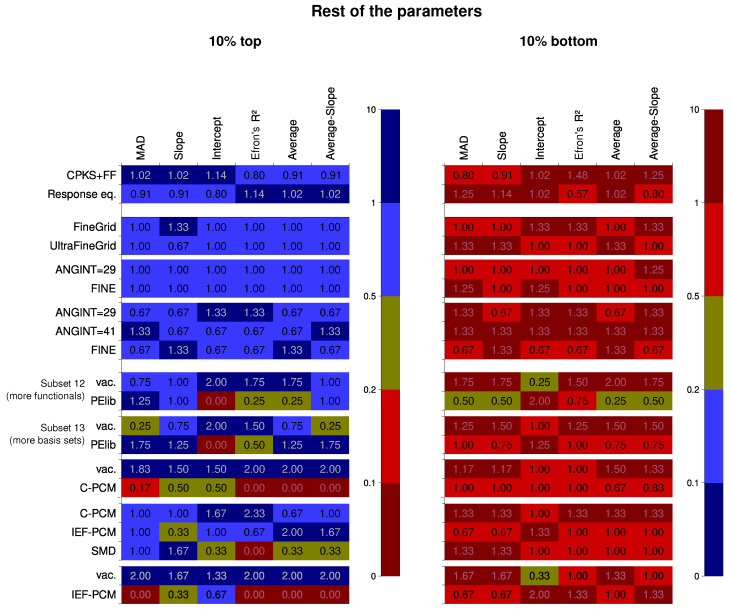
Performance of specific statistical levels of other factors, calculated as frequency thereof within the top 10% and bottom 10% bins of the Data Subsets 3, 6–8, and 11–19. The closer the color is to dark red, the worse is performance; the closer the color is to dark blue, the better the performance. The numbers show the normed probability of a treatment to fall into the 10% or 10% worst bin; because of this, higher values are colored blue for the “10% best” pane but dark red for the “10% worst” pane.

Parameters other than the basis set and the Hamiltonian were tested for the rest of the data subsets. The results of the analysis are presented in Figure 7. There is little difference between the computational approach to the second hyperpolarizability (CPKS+FF vs. response-equation formalism). Only for the $R^{2}$, there is a slightly better performance of the RE formalism. The integration grid does not influence the results much, even though for the comparison between FineGrid and UltraFineGrid, a meta-hybrid functional M06-2X was present in the data. This could be explained by the fact that the orientation of the molecules did not change during the study (this can alleviate the grid dependence [150]).

The situation is much more intriguing for the solvation factor. The study within the CPKS+FF formalism features a strong advantage of vacuum vs. PCM calculations in terms of the quality of the results, evident for the top-10% bin. For the bottom-10% bin, however, the reverse is true: calculations in the presence of a solvent are less prone to produce extremely bad results. The prevalence is not strong, nevertheless. It could have been expected that the reason for this is that the simplest variant of the PCM model, the C-PCM, was employed. Yet, the comparison among the three different PCM models basically shows the opposite (although for the more strictly defined IEF-PCM, fewer results are found in the bottom-10%-tear). It could be tempting to think that some error compensation leads to this. For the average descriptors, the performance of IEF-PCM is slightly better than for C-PCM, however. The SMD approach greatly improves the results for the slope but performs worse for anything else. The question of whether the IEF-PCM results are still worse than those of calculations in vacuum prompted us to check this separately on the available dataset. However, across the top-10% bin, the vacuum results still readily outperform IEF-PCM ones, even more strikingly than in the case of C-PCM vs. vacuum comparison. The result for the bottom-10% bin is, similarly, less in favor of IEF-PCM over the vacuum than it was for C-PCM over the vacuum.

On the other hand, the performance of the PELib model is much better for the response-equation formalism data subset. It is virtually impossible to tell whether the inclusion of the solvation model into calculations improves or worsens the result, except for two descriptors: the intercept and $R^{2}$. For these, the inclusion of PELib is, unfortunately, detrimental.

Overall, solvent models are still not ready to provide consistently better results for the second hyperpolarizability, but PELib is close to at least matching the accuracy of vacuum calculations.

**Figure 8:**
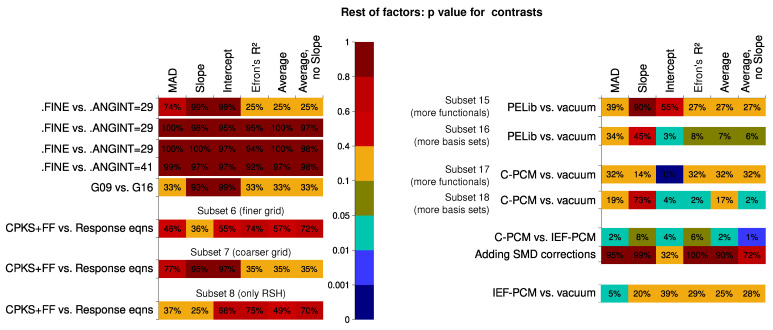
Statistical significance of certain linear contrasts for the different computational quality descriptors, Data Subsets 3, 6–8, and 11–19 (see the main article text for the clarifications). Significance is given by the *p* value of the F tests within the contrast-based analysis of variance (ANOVA).

The contrasts for the other parameters (displayed in Figure 8) show that sometimes the contrast is seemingly substantial for the formalism used and the integration grid, but this is most probably because of a lower number of factor levels available. The influence is, of course, much more significant for the solvation factors. The largest contrasts are between C-PCM and IEF-PCM, which were also an important reason to check the results for the data Subset 20.

The most sensitive descriptor is again the intercept, whereas the least sensitive is the slope.

#### 6.2.2. Computation Time

**Figure 9:**
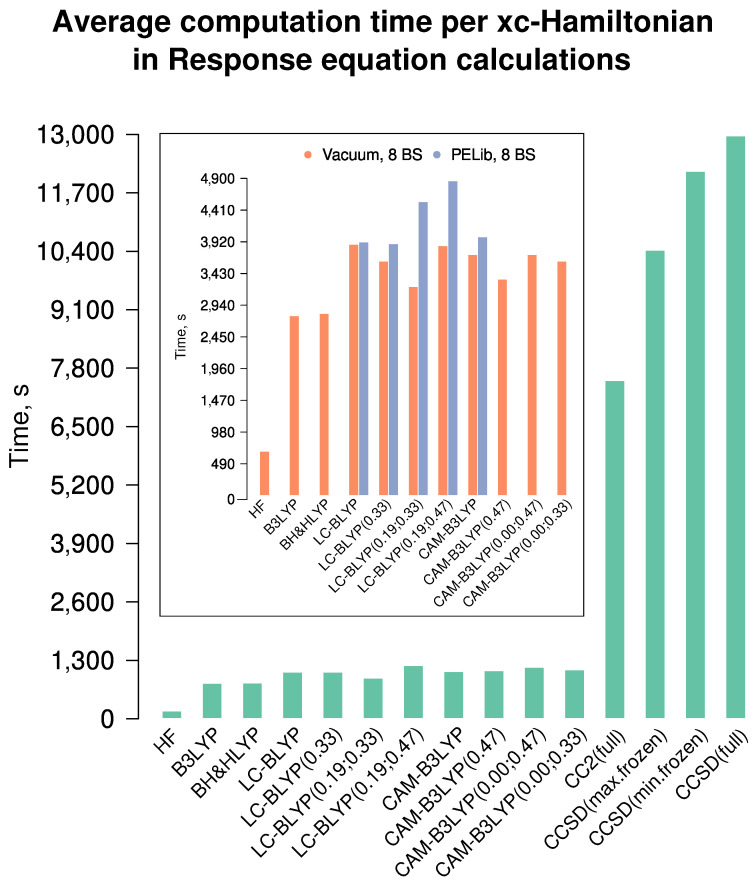
Average computation times (s) for various exchange–correlation Hamiltonians in Dalton. All calculations in this part were performed on 40-core cluster nodes. In the main part, five basis sets were used for each dataset, and the averages were concluded; for the inset part, eight basis sets were used for the average.

Before the conclusions are drawn, we also must discuss the time used for computation, as can be seen in Figure 9 and Figures S2 and S3 in the Supporting Information. We decided not to serialize the calculations but to use parallel ones, to give the reader a practical perspective (depending, among other factors, on how well the code is parallelized). As can be concluded, CCSD calculations are by an order of magnitude more time-expensive than ones employing RSH DFT (it is essential to note that the CC calculations of the second hyperpolarizability are not parallelized in Dalton 2020.0.1). Interestingly, the maximal freezing of core orbitals has no significant effect on CCSD results but reduces the computation time by ca. 20%. The reduction is modest because the molecules studied in this research comprise predominantly light atoms. CC2 is significantly cheaper but gives poor results. RSH density functional calculations are only by ca. 25% more expensive than the global-hybrid ones. HF calculations are very cheap, and, as they still produce good correlation quality, can be used for time-efficient screening of compounds as the first step in a screening protocol. The influence of PELib solvation is around a 20% increase in computation time in the parallelized Dalton.

For calculations in Gaussian (see Figure S2), we see that adding C-PCM to the Hamiltonian only causes a marginal increase in the computation time (2–4%, except for HF because of its relatively low cost in general). Meta functionals are slightly more costly than the range-separated hybrid ones, but the increase is only about 25%.

Considering the basis sets (in Dalton), there is an astounding effect on computation time when we add polarization functions in large quantities (see Figure S3). The time grows almost 6 times when going from 6-311++G(d,p) to 6-311++G(3df,3pd), in which case the number of polarization functions grows 4.5 times on average for CHCl_3_ and nitrobenzene. Moreover, the time skyrockets almost 10 times when going from aug-cc-pVDZ to aug-cc-pVTZ, when the number of polarization functions increases 3.5 times on average for the same two molecules, and the number of valence functions also grows 1.5 times. Sadlej-pVTZ, aug-cc-pVDZ, and 6-311++G(d,p) are close in the average computation time (difference of 7–15%). Interestingly, adding diffuse functions on hydrogens more significantly increases the computation time than adding them on heavy atoms only (74% vs. 42% when going from 6-311G(d,p) through 6-311+G(d,p) to 6-311++G(d,p)). This stipulates the use of the high-scoring Sadlej-pVTZ instead of the significantly more expensive aug-cc-pVTZ.

#### 6.2.3. Effect of Hydrogen Bond Formation and Multiple Conformers Possible

To find out whether the presence of hydrogen bonds in the solvent does change the absolute value of the second hyperpolarizability, we constructed oligomers of alcohol molecules and water (the structures are available in Supporting Information). These calculations were performed in Gaussian 16 (with CPKS+FF methodology, including C-PCM description of the solvent into the model). Then, we compared the γ value for the monomer with the average γ value per molecule within a H-bonded cluster (at least four different clusters for every compound, including those with different organization of the same number of molecules, mostly linear and cyclic ones).

**Figure 10:**
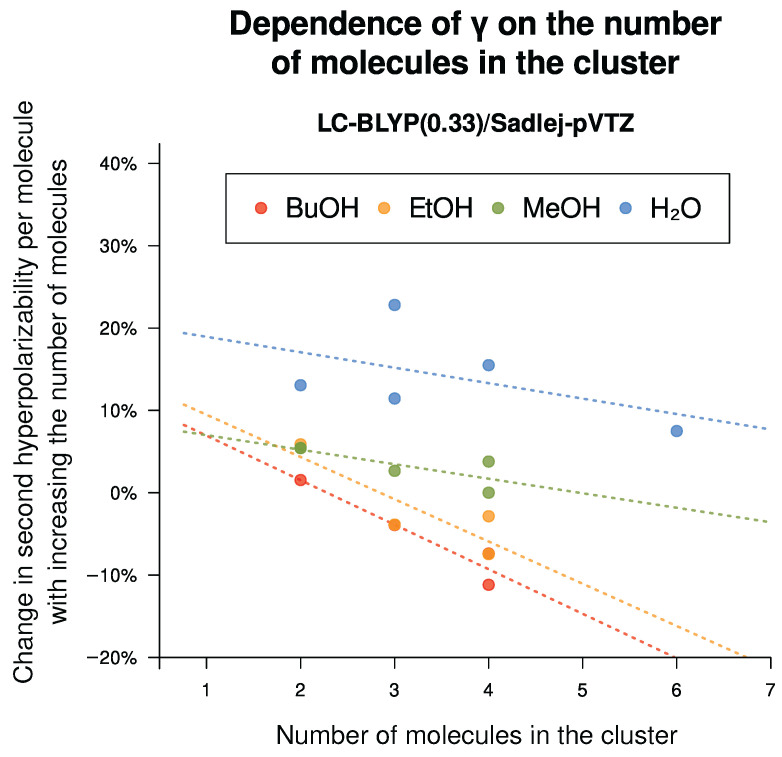
Dependence of the second hyperpolarizability on the number of molecules in a cluster. Calculations were performed with CPKS+FF, LC-BLYP(0.33)/Sadlej-pVTZ in vacuum. Multiple clusters for the same number of molecules usually means a linear and a cyclic one.

The dependence of various molecules is depicted in Figure 10. For smaller molecules (water and methanol), the average γ per monomer in a cluster is always larger than that of an isolated monomer; for higher alcohols and better-performing basis sets, γ only grows for the dimer and falls below that of the isolated monomer for larger clusters.

The influence of these findings on the correlation parameters would be as follows. If the calculated γ value increases or stays put for the water and decreases for the higher alcohols, it must follow that the slope of the correlation must increase (with the intercept decreasing and the effect on the $R^{2}$ varying). From Figure 1 and Figure 2, we can see that the points of water and alcohols typically lie on the right side of the correlation line, which indicates computational overestimation. In this case, the results on $R^{2}$ and (for CAM-B3LYP) on the correlation slope may actually be improved. However, we did not include the clusters in the main analysis, as deducing which of them are actually present in the bulk is well beyond the scope of the present study.

We have also considered the effect of various conformations with potentially different hyperpolarizability, which affects molecules both in the middle and the upper parts of the set (sorted by the absolute value of γ). The optimal geometry is hard to predict, and this is definitely beyond the scope of the present study; however, calculations on 1-buthanol show the variation in value only within 4–6% for the better-performing basis sets.

## 7. Conclusions

The present study tested a variety of computational options to assess the value of the molecular dipolar second hyperpolarizability. We may conclude the following:

1. Presence of diffuse functions is mandatory in a basis set used for the calculations of the second hyperpolarizability. Adding large amounts of polarization functions, contrariwise, does not improve results but leads to great computational expenses. Sadlej et al.’s basis set (POL or Sadlej-pVTZ) [104,105] is computationally very robust and resource-efficient, and thus highly recommended for calculations. The basis set aug-cc-pVTZ is only slightly and only sometimes performing better, yet it is much more resource-demanding.
2. LC-BLYP(0.33)/Sadlej-pVTZ is a very well-performing choice of model chemistry in terms of both predictive power and computational efficiency.
3. HF/Sadlej-pVTZ is a very good model chemistry for fast and crude screening of compounds. To increase its predictive power, the slope and the intercept should be determined using some more advanced reference calculations.
4. Coupled-cluster (CC) formalism as implemented for the response-equation formalism in Dalton 2020.0.1 is not well suited for the production calculations of the second hyperpolarizability, possibly because the formalism is not yet well implemented. In addition, coupled-cluster calculations are rather sensitive to the basis set used.
5. Even the maximal available freezing of the core orbitals does not diminish the performance of the CC methods but significantly decreases the resource consumption.
6. The meta-global-hybrid density functionals are not suited for the calculations of the second hyperpolarizability. Also, they are basis-set-dependent and basis-set-inefficient.
7. In the construction of a range-separated hybrid density functional (RSH) for the calculations of the second hyperpolarizability, it is generally important to have comparatively low HF-like exchange percentage in the far- and middle-range inter-electronic distances and (to a lesser extent) even slightly lower percentage in the close range. It is not worthwhile to use the overall average percentage of the HF-like exchange as a parameter for optimizing the results. However, a specific combination of RSH parameters can produce better results than following these rules.
8. The mean absolute deviation (MAD) descriptor provides less efficient discrimination between factors when comparing different quantum chemical methods. The correlation descriptors should be used instead. The most susceptible descriptor is the intercept of the linear correlation.
9. There is little difference in whether the coupled-perturbed KS/HF plus finite field (CPKS+FF) or the response-equation formalism is used for the calculation of the second hyperpolarizability.
10. Calculations in the vacuum are still more robust than calculations with contemporary solvent models included in the Hamiltonian. However, the PELib model is close in its performance to the vacuum calculations and never yields results that are particularly far from the experiment in terms of statistical descriptors. IEF-PCM and SMD do not outperform the simple C-PCM solvation model.

## Figures and Tables

**Table 1:** Summary of methodologies for computation of the second hyperpolarizability γ (see text for details).

Methodology	Static γ	Dynamic γ	Model Chemistries	Benefits	Challenges
FF	Yes	No	HF, DFT, MP*n*, CC*n*, MCSCF	Simple implementation; Efficient for large systems	Selecting field strength; Numerical differentiation challenges
CPKS+FF	Yes	Partially *	HF, DFT	Comparatively easy to implement	* Numerical differentiation challenges
RE+FF	Yes	Partially	HF, DFT, CC*n*, MCSCF	Comparatively easy to implement	Numerical differentiation challenges
Fully analytical RE	Yes	Yes	HF, DFT, CC*n*, MCSCF	Full frequency dependence (incl. OKE)	Undertaking to implement
Fully analytical CPHF	Yes	Yes	HF, DFT	Full frequency dependence (incl. OKE)	Undertaking to implement
SOS	Yes	Yes	HF, DFT, MP*n*, CC*n*, MCSCF	Easy to implement; Full frequency dependence (incl. OKE)	In practice, truncation is mandatory, which requires tuning for particular systems

* Only for certain frequency combinations (not OKE).

## Data Availability

The data that support the findings of this study are available on request.

## Supplementary Materials

The following supporting information can be downloaded at: https://www.mdpi.com/article/10.3390/nano15171302/s1, Figure S1: The results of the test set of geometry optimization method, excluded from the main study. Figure S2: Basis set dependence of the computational time, in CPKS+FF calculations. Figure S3: Basis set dependence of the computational time, in RE calculations. Figure S4: Popularity of quantum chemical computational software for calculations of the second hyperpolarizabilities. Figure S5: Popularity of two-electron Hamiltonians for calculations of the second hyperpolarizability. Figure S6: Number of functions of different types for several basis sets, including those used in the present study, for two representative molecules. Table S1: Design matrix for the range-separated density functionals of the LC-type. Table S2: Proportion of the non-normal data series among the datasets. Table S3: Excitation energies (in nm) obtained using linear-response and state-specific (IBSF) TD-DFT/TD-HF methods for selected compounds. Table S4: TD-DFT computed excitation wavelengths and oscillator strengths (f) for CS_2_, DMSO, and nitrobenzene.

## Abbreviations

The following abbreviations are used in this manuscript:

a.u.	Atomic Units
C-PCM	Conductor-like Polarizable Continuum Model
CC2	Coupled-Cluster Singles and Approximate Doubles
CCSD	Coupled-Cluster Singles and Doubles
CC*n*	Coupled Cluster (in general)
CPHF	Coupled-Perturbed Hartree–Fock
CPKS	Coupled-Perturbed Kohn–Sham
DFT	Density Functional Theory
DMSO	DiMethyl SulfOxide
DMFA	N,N-DiMethylFormAmide
FF	Finite Field
FFD	Full-Factorial Design
GH	Global-Hybrid [density functionals]
HF	Hartree–Fock
IEF-PCM	Integral Equation Formalism PCM
MAD	Mean Absolute Deviation
MCSCF	Multi-Configurational Self-Consistent Field
MGH	τ-Meta-Global-Hybrid [density functionals]
MP2	Møller–Plesset Perturbation Theory (second-order)

MP*n*	Møller–Plesset Perturbation Theory (in general)
OKE	Optical Kerr effect
PCM	Polarizable Continuum Model
RE	Response Equation
RSH	Range-Separated-Hybrid [density functionals]
SMD	Solvation Model based on Density
SOS	Sum-Over-States
TDDFT	Time-Dependent Density Functional Theory
