# Supplementary material for: Use of Factorial Design for Calculation of Second Hyperpolarizabilities
Source: Nanomaterials (Basel). 2025 Aug 23;15(17):1302. doi: 10.3390/nano15171302 (PMC12430289; doi:10.3390/nano15171302)
Supplement: Supplementary file 1 [file nanomaterials-15-01302-s001.zip › figs/FigS1.pdf]

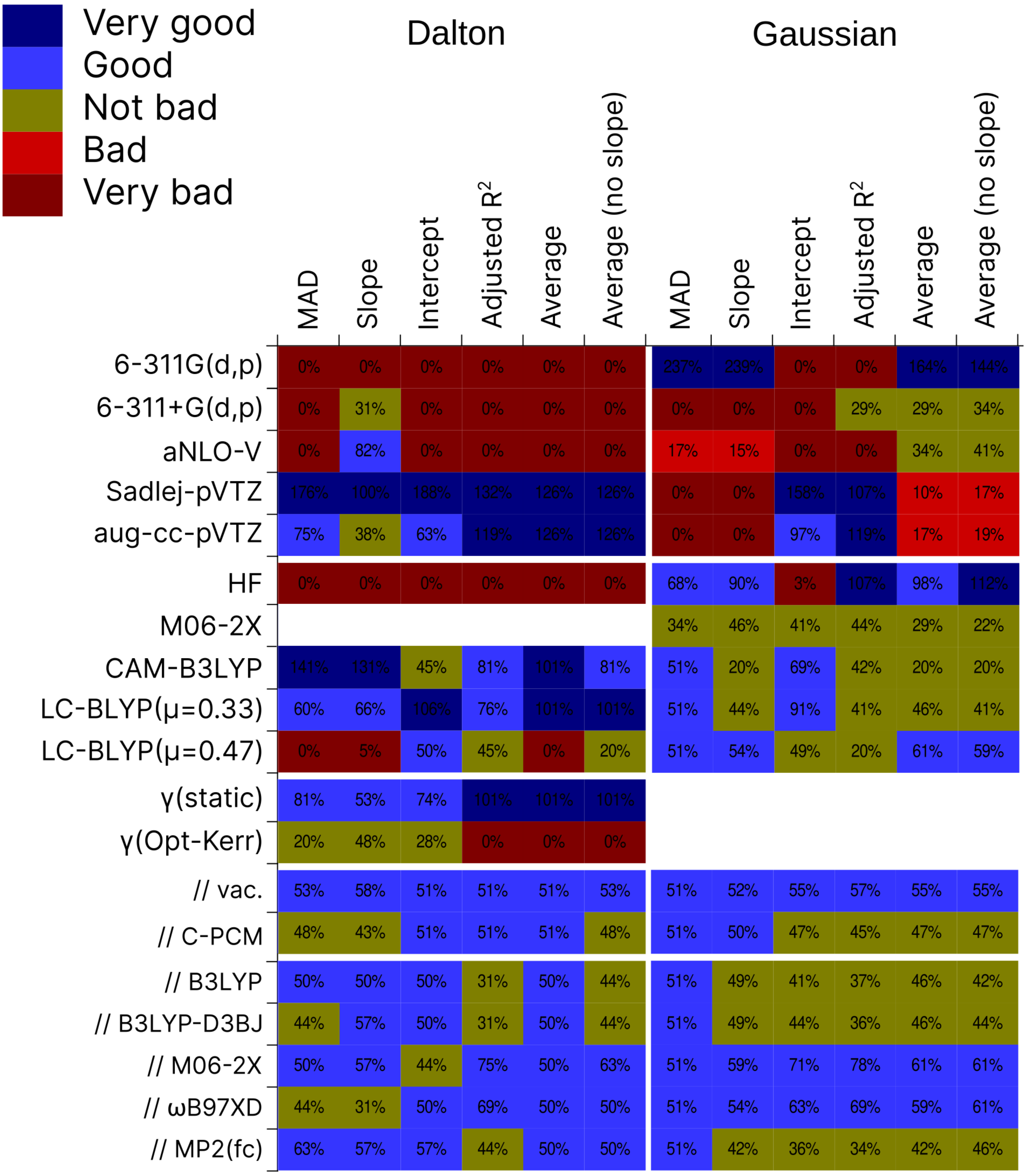
Very good
Good
Not bad
Bad
Very bad
Dalton
Gaussian
MAD Slope Intercept Adjusted R2 Average Average (no slope) MAD Slope Intercept Adjusted R2 Average Average (no slope)
6-311G(d,p) 0% 0% 0% 0% 0% 0% 237% 239% 0% 0% 164% 144%
6-311+G(d,p) 0% 31% 0% 0% 0% 0% 0% 0% 0% 29% 29% 34%
aNLO-V 0% 82% 0% 0% 0% 0% 17% 15% 0% 0% 34% 41%
Sadlej-pVTZ 176% 100% 188% 132% 126% 126% 0% 0% 158% 107% 10% 17%
aug-cc-pVTZ 75% 38% 63% 119% 126% 126% 0% 0% 97% 119% 17% 19%
HF 0% 0% 0% 0% 0% 0% 68% 90% 3% 107% 98% 112%
M06-2X 34% 46% 41% 44% 29% 22%
CAM-B3LYP 141% 131% 45% 81% 101% 81% 51% 20% 69% 42% 20% 20%
LC-BLYP(μ=0.33) 60% 66% 106% 76% 101% 101% 51% 44% 91% 41% 46% 41%
LC-BLYP(μ=0.47) 0% 5% 50% 45% 0% 20% 51% 54% 49% 20% 61% 59%
γ(static) 81% 53% 74% 101% 101% 101%
γ(Opt-Kerr) 20% 48% 28% 0% 0% 0%
// vac. 53% 58% 51% 51% 51% 53% 51% 52% 55% 57% 55% 55%
// C-PCM 48% 43% 51% 51% 51% 48% 51% 50% 47% 45% 47% 47%
// B3LYP 50% 50% 50% 31% 50% 44% 51% 49% 41% 37% 46% 42%
// B3LYP-D3BJ 44% 57% 50% 31% 50% 44% 51% 49% 44% 36% 46% 44%
// M06-2X 50% 57% 44% 75% 50% 63% 51% 59% 71% 78% 61% 61%
// ωB97XD 44% 31% 50% 69% 50% 50% 51% 54% 63% 69% 59% 61%
// MP2(fc) 63% 57% 57% 44% 50% 50% 51% 42% 36% 34% 42% 46%
