# Supplementary material for: Use of Factorial Design for Calculation of Second Hyperpolarizabilities
Source: Nanomaterials (Basel). 2025 Aug 23;15(17):1302. doi: 10.3390/nano15171302 (PMC12430289; doi:10.3390/nano15171302)
Supplement: Supplementary file 1 [file nanomaterials-15-01302-s001.zip › figs/FigS5.pdf]

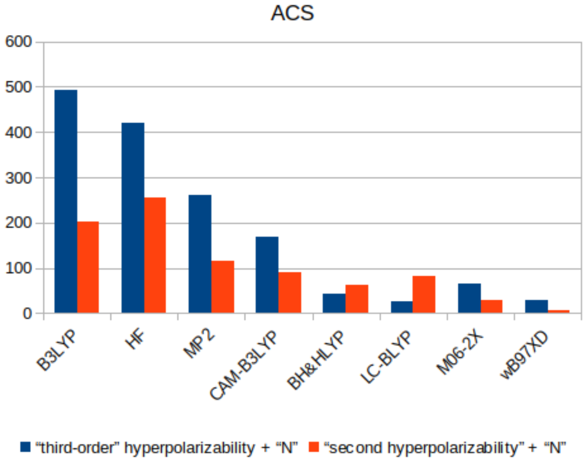
ACS
0
100
200
300
400
500
600
B3LYP
HF
MP2
CAM-B3LYP
BH&HLYP
LC-BLYP
M06-2X
wB97XD
"third-order" hyperpolarizability + "N"
"second hyperpolarizability" + "N"

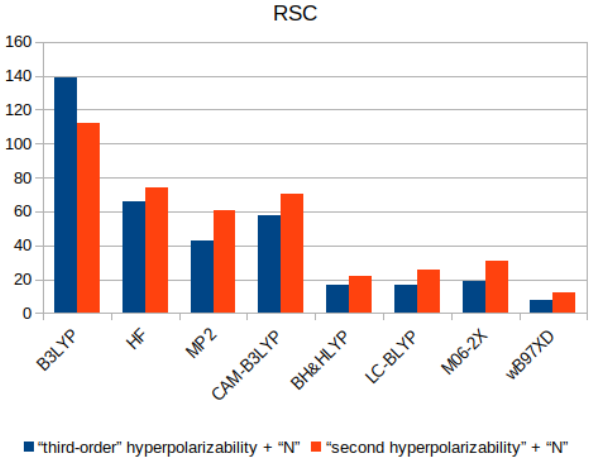
RSC
0
20
40
60
80
100
120
140
160
B3LYP
HF
MP2
CAM-B3LYP
BH&HLYP
LC-BLYP
M06-2X
wB97XD
"third-order" hyperpolarizability + "N"
"second hyperpolarizability" + "N"

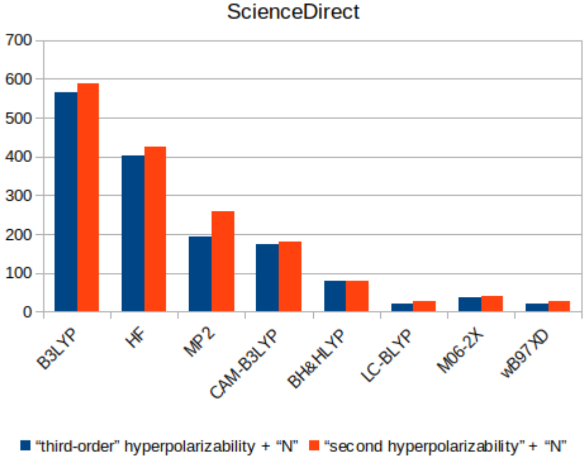
ScienceDirect
0
100
200
300
400
500
600
700
B3LYP
HF
MP2
CAM-B3LYP
BH&HLYP
LC-BLYP
M06-2X
wB97XD
"third-order" hyperpolarizability + "N"
"second hyperpolarizability" + "N"

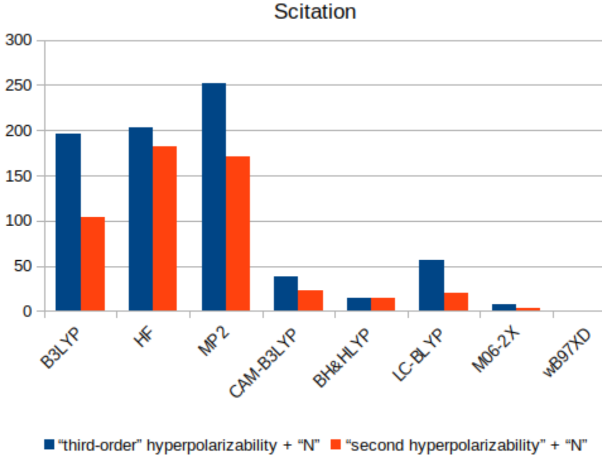
Scitation
0
50
100
150
200
250
300
B3LYP
HF
MP2
CAM-B3LYP
BH&HLYP
LC-BLYP
M06-2X
wB97XD
"third-order" hyperpolarizability + "N"
"second hyperpolarizability" + "N"

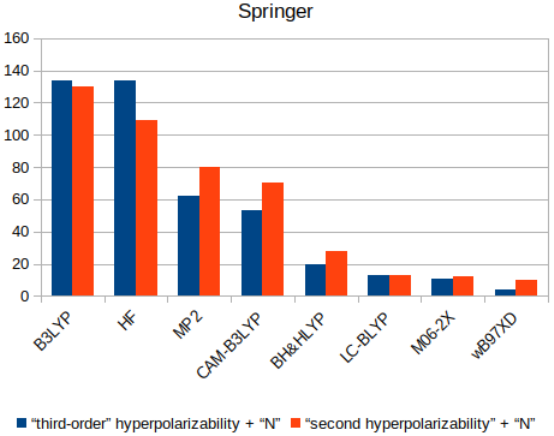
Springer
0
20
40
60
80
100
120
140
160
B3LYP
HF
MP2
CAM-B3LYP
BH&HLYP
LC-BLYP
M06-2X
wB97XD
"third-order" hyperpolarizability + "N"
"second hyperpolarizability" + "N"

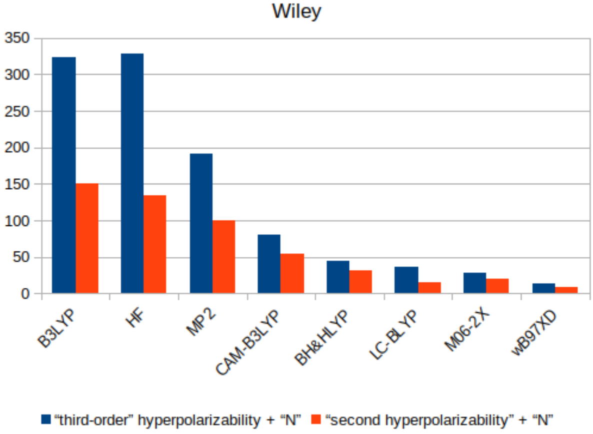
Wiley
0
50
100
150
200
250
300
350
B3LYP
HF
MP2
CAM-B3LYP
BH&HLYP
LC-BLYP
M06-2X
wB97XD
"third-order" hyperpolarizability + "N"
"second hyperpolarizability" + "N"
