# Supplementary material for: Use of Factorial Design for Calculation of Second Hyperpolarizabilities
Source: Nanomaterials (Basel). 2025 Aug 23;15(17):1302. doi: 10.3390/nano15171302 (PMC12430289; doi:10.3390/nano15171302)
Supplement: Supplementary file 1 [file nanomaterials-15-01302-s001.zip › nanomaterials-3796781-supplementary.pdf]

Supplementary Materials: Use of Factorial Design for Calculation of Second Hyperpolarizabilities

Igors Mihailovs [1,2,3,*], Ekaterina Belobrovko [1,4], Arturs Bundulis [1], Dmitry Bocharov [1,5], Eugene A. Kotomin [1*], Martins Rutkis [1]

1. The Short Literature Review

The following is the discussion of various other "parameters" needed to compute the second hyperpolarizability. As studies on computing γ are not too numerous, we also included studies discussing these "parameters" for β calculations; this should not hamper the analysis because there is a great deal of correlation between these quantities.

Often, it is chosen by researchers to use static values of hyperpolarizability when benchmarking computational methods against their higher-level counterparts (as dynamic calculations are, contrary to FF, not available for all methods, especially those of higher precision). However, it is also well known that polarizabilities of higher order exhibit very pronounced frequency dependence [1]. Also, direct comparisons between finite-field and fully analytical RE calculations suggest that in certain cases the correspondence is not good enough [2], and the ratio between FF and static SOS (as well as CPHF) is noticeable and varies from method to method [3]. Another study revealed that the performance of different methods changes noticeably when going from static to dynamic values of hyperpolarizability [4]. Therefore, it may be beneficial for the correct description of OKE to use the value of γ calculated at the frequency employed in the experiment and to compare it with the experimental data. In fact, many studies comparing the calculated values with experimental ones tend to use the dynamic calculated quantities. When the dynamic value cannot be calculated, a fairly common workaround is to obtain the dynamic factor from one method and multiply it by the static value from another method, which is deemed more precise. This is often employed for MP2 calculations (quite logically, the dynamic coefficient is usually taken from CPHF) [4–7].

Another factor in the calculation is the solvent effects, the importance of which is well-established for hyperpolarizabilities [8–11] and is more pronounced for higher-order quantities [5], more [7] or less [5] for dynamic ones. On the other hand, it is frequently that the molecule's geometry used in calculations is optimized in vacuum [12], not in the solution. At least for electronic spectra (a property directly related to hyperpolarizabilities), it is known that the changes in geometry due to medium polarity change and their effect on electron distribution are usually in the opposite direction to the direct changes in electronic structure [13]. There is also direct evidence that the relative performance of different methods changes [4,14,15] (and absolute performance improves [14]) when geometries are re-optimized with solvent effects taken into account. Also, even in comprehensive studies geometry of the molecule sometimes remains unchanged, optimized at the same theoretical level [7,8,12,16–21], which can also introduce additional uncertainty to results. All this is especially evident if we consider the fact that one of the most crucial [22–24] geometry characteristic of push–pull polyenes (which are typical NLO-phores), the bond-length alternation, is known to be method- [23–28] and medium-polarity-dependent [29,30], and so are dihedral angles, too [31–34], as well as other properties [35]. Truly, it is known that these indirect effects (through geometry) are weaker than the direct effects (through computation method of the property itself) — but the former can reach 150% [26] and beyond [32] in certain cases.

For small molecules, there is usually not much discussion about which method to use for the geometry optimization, and the data in literature reviews is usually quite outdated in terms of methods used, as for small molecules difference is usually negligible [36,37]. It is quite established, though, that triple-zeta basis sets provide reasonable accuracy for most non-sophisticated cases [36]. As the property we are interested is of quite high order, we reckoned that one could obtain some additional insight into the problem by varying the functional used for the geometry optimization, too, for which we selected the most-frequently used B3LYP [8,38–40], praised M06-2X [37,40–42] and MP2 [37], as well as dispersion-corrected functionals ωB97XD and B3LYP-D3BJ which also tend to perform well [41].

It has been a somewhat established opinion (or stereotype) that pure GGA and global-hybrid density functionals (with a rare exception) are not up to the task of computing second-order hyperpolarizabilities, whereas MP2 and range-separated hybrid density functionals are performing well [11,43,44]. In terms of method, often B3LYP global-hybrid density functional is used, especially in single-molecule descriptive studies [35,45–68]. This functional is, however, often found to have quite low performance in calculations of hyperpolarizabilities [3,8,14,19,69–72]. Quite frequently, Hartree—Fock theory is still used, owing to its long-known fair performance for this task [2–4,17,38,39,59,73–78]. One of the most popular [11,31,35,40,42,49,79–88] (see Section B in Supporting Information) and reasonable [11,12,19,43,70–72,89–92] alternatives among density functionals is CAM-B3LYP. Sometimes others are employed, such as LC-BLYP [43,93] and other functionals of the LC series. Another rather popular range-separated functional is ωB97XD [62,94]. The ωB97XD functional is found to have similar performance as CAM-B3LYP [43,95], closer to MP2 values of γ than LC-n series [95], which would suggest better performance, as it is known that MP2 values are usually overestimated somewhat in comparison with MP4 [96]. In another study, on the contrary, it was also found that the simplest form of the range-separated hybrid (RSH) functionals (LC-n) performed better than more sophisticatedly designed ωB97-n series against experimental values of β_μ (the projection on the dipole moment) [8] or against CCSD(T)-derived β_{HRS} values [14]. The case of ωB97-n is supported (against CAM-B3LYP, at least) for other groups and also for γ, this time against the experimental values [71]; ωB97XD is, however, not the most accurate functional within the ωB97-n series [8,12,14,16,71], often [7,69,72] (not always [97]) performing worse than CAM-B3LYP and known to have relatively significant basis-set dependence [98].

In general, there is somewhat conflicting evidence about the optimal configuration of the long-range corrected density functionals. For example, Lu et al. [8] found (on an extensive experimental data set of the first-hyperpolarizability projection onto the dipole moment from EFISHG measurements) that 100% of exact exchange (EXX) in the long range is mandatory for qualitative predictions (incl. HF and M06-HF Hamiltonians). On the opposite, Wang et al. [16] found (on the data set of γ from the four-wave mixing experiment for 4 streptocyanine dyes) that the experimental trends are best described by non-hybrid functionals (PBE and M06L) or by IP-tuned range-separated hybrids with a low amount of EXX even at 10 Åinter-electron distance; these results were obtained through application of SOS methodology but supported by SOS/SAC-CI computations. The problem with SOS is that it is known to converge only after adding rather many states (50 or more [98]) to the approximation, and the biggest increase comes only after 5—20 steps, evidence that the approximation may be misjudged to be converged; this does not, however, apply to Wang et al., as they checked the convergence for up to 300 states and used 100 states for production calculations [16]. Suggestion that pure density functionals perform better than hybrid ones may refer only specifically to that one case, as in multiple studies, the opposite was found [17,69,99]. There are also other studies suggesting that "LR—100%EXX" RSHs are

somewhat outperforming the alternatives (like CAM-B3LYP) [14,17,71,72,90,97,100,101] but not always [70]. However, it is frequently (but not always) observed that the best ω (or μ) of RSH is not the default 0.47 but 0.33 (like in CAM-B3LYP) or close to it [43,90,100,102]](for bimetallic complexes with σ–, not π–dominated γ, this choice can be quite bad, though [103]). In a recent study, it was discovered that LC-BLYP performs better than all other tested methods in protic solvents but somewhat worse than CAM-B3LYP, M05-2X, and M06-2X — in aprotic ones [4]. It now also becomes more frequently used in non-benchmark studies [104,105].

Also, global hybrids with a relatively large amount of EXX (40—50%) are quite frequently [90] praised for their utility (with exceptions [8]), in particular, Minnesota meta-hybrid functionals M06-2X and M05-2X [7,14,70,74,89–92,101] (not always of similar performance [14]) and the global-hybrid BHandHLYP (also known as BHHLYP and BH&HLYP) [18,19,69,71,72,84,91,106]. The problem with the latter can be a higher basis-set dependence of the quality of results in comparison with. e.g., CAM-B3LYP [98]; also, in certain cases, it performs much poorer than on average [14].

Meta-functionals might perform similarly [11,70] to or even better than [42] that of RSH functionals. For example, in Ref. [11] M06-2X is found to yield considerably better results than the more modern MN15 (it contains slightly less EXX and its GGA part is also constructed differently, belonging to the non-separable gradient class of GGA); another study of the same team had the opposite result, though [70]. Some studies even employ M06 out of general considerations [107].

Considering the ω parameter, the well-known scheme of "optimal tuning" (OT-RSH), in which this parameter is adjusted for each molecule until Janak's theorem is satisfied, is considerably [8,72]20,114 but not always [70,100,108] successful for computing the hyperpolarizabilities. As it involves a non-trivial computational procedure (i.e., beyond putting certain keywords in the input file), which may be unsatisfactory for a general audience, we excluded this option from our study. Double-hybrid functionals (as B2PLYP) generally (but not always [108]) do not perform better than RSH or even BHandHLYP [4,14,17,19,20,69] and were therefore also excluded from the present study.

The uncertainty in method is not restricted to the DFT realm but is also observed in the MPn series [32] and CCn series [19], or both [109]. MP2 results are quite frequently used as reference values [4,12,89,95,110]; or cited as outperforming density functionals of any form (sans double-hybrid ones, closely related to MP2) [108]. However, while MP2 calculations are often more than capable of correctly describing hyperpolarizability [14,28,74,90,92,101,108], it is now known that MP2 can be deficient in particular cases [3,17,21,111]. Moreover, MP2 can only provide finite-field static values from a numerical differentiation procedure; for a quantity of such a high order as γ is, the poor performance is therefore more or less expected. CCSD(T) is frequently used as a high-quality reference [42,70,108,112–114]. Productivity of CCSD (without the perturbation corrections) was questioned before [19,95,109], but other studies found it to be not much worse than CCSD(T) [90] or CC3 [115,116]. Inclusion of triple correction as a requirement for certain cases has been known for a long time [117]. The performance of CC2 is found to differ from that of CCSD for solvent molecules, but not as much as the CCS one [118]. Other studies report that CC2 yields results much closer to EOM-CCSD than CAM-B3LYP for the two-photon absorption cross-section [113] and should at least outperform MPn methods [114]. Besides CCn and MPn series, multi-reference methods such as CAS-SCF and its derivatives are sometimes employed in predicting hyperpolarizabilities [19,119,120]; they do not, however, enjoy popularity in the field because of a non-trivial setup in most cases. Results by CAS-SCF and CCSD are sometimes quite different [19].

Importantly, as has been recently found out, density functionals that perform well for the first hyperpolarizability may fail to do so for the second one [108].

There is also no consensus about how large and what type of basis set to use. It is more or less well-established that *some* (particularly, some d) polarization [3,5,12,17] and *some* diffuse functions [3,5,18,95,104] on heavier-than-H atoms are essential for a reliable description of hyperpolarizabilities. The importance of diffuse functions is found to systematically exceed that of polarization functions on heavy atoms [5,95] or sometimes to match them in terms of importance [5], and to be generally more important than adding valence shells [5,18,95]. In the latter study, adding diffuse functions increased γ by $\sim$10% while adding polarization decreased it by 3 to 12%; in another study [95] considering 3rd-row element clusters, the increase in value of β after adding diffuse functions was 25—70% for double-zeta and 15—40% for triple-zeta bases while adding additional polarization functions cause major oscillations (up to 20%) in predicted value, less pronounced for higher-zeta and especially — for already augmented basis sets. The last conclusion suggests that adding additional polarization functions to non-augmented or small-zeta basis worsens their quality (so-called "over-polarization"). Sometimes adding even a very large quantity of polarization functions essentially does not change the result [12,17]. A similar effect is also observed for diffuse functions ("over-augmentation") [3,99]. The common and somewhat solid conclusion is that there is no point in using polarization or diffuse functions on hydrogen atoms (probably except for cases when it is more electronegative than its bonded peer) [5,12,18]. Also, increasing the number of "standard-sized" valence shells generally raises the values of hyperpolarizabilities (less than adding diffuse functions [5,95] but can also decrease them if diffuse functions are already present, thus stabilizing the basis set [95]. In general, smaller molecules and those comprising heavier elements are more sensitive to changes in basis set [95], and already big basis sets have smaller benefits from further upgrades [5,95], but diffuse functions are required in any case. If the basis set is sufficiently large, there is no big difference between one with more diffuse functions (aug-cc-pVTZ), one with more polarization functions (aug-pc-2), and one similar to aug-pc-2 but with even fewer diffuse functions (6-311++G(3df,3pd)) [98].

Another option is some special-purpose basis set, for example, one by Paschoal and Dos Santos) [17] or the one designed by A. Sadlej et al. [121,122] (named Sadlej-pVTZ [123,124] or POL [125] in different sources) specifically for calculations of [hyper]polarizabilities; this basis works very well in some cases [2,20,99,112,126] but less well in other cases [19,99]. These basis sets were constructed by removing redundant functions and re-optimizing the basis set to better reproduce the property under consideration. It is indeed observed in certain cases that even just removing unnecessary diffuse functions from a heavily augmented basis set (aug-cc-pVDZ) improves results to put them on par with aug-cc-pVTZ or daug-cc-pVDZ [126].

It is sometimes incorrectly assumed that basis sets from different families with analogous designations are essentially the same. In reality, for example, Pople basis sets are much less equipped with diffuse functions and (often) also much less equipped with polarization functions than Dunning's correlation-consistent basis sets. For example, for a nitrobenzene molecule an in-field popular [2,19,47,52–55,60,62,66,68,73,82,97,127] Pople-family basis set 6-311++G(d,p) [128–131] provides 243 contracted functions, whereas Dunning's basis set with seemingly analogous name – aug-cc-pVTZ [132–134] –, provides astonishing 529 contracted functions (particularly, *more than 5 times more* diffuse functions; in fact, aug-cc-pVDZ [133–135] is closer — and *still larger* — with 340 functions). Thus, of course, still raises the question: which basis set to use, as calculations with more diffuse and (especially) polarized functions usually require more computational time and resources, create instabilities in calculation [19] as well as introduce [2,101] the basis-set superposition error (mostly for

small molecules, sometimes up to 100% [2]). Indeed, many studies confirmed that smaller basis sets like 6-311++G(d,p) often perform not worse than Dunning's analogues [2,69] (or even aug-cc-pVQZ sometimes [19]).

2. The Details of the Quantum Chemical Calculations Performed

a. CCSD calculations were carried out in three different ways: 1) with all shells active, CCSD(full); 2) with only the most core levels frozen on all atoms, CCSD(min-fc); 3) with all but the valence shells frozen on all atoms, CCSD(max-fc). In CC2 calculations, all shells were active, hence it bears a designation CC2(full); frozen-core CC2 is not available in Dalton 2020.

b. Coupled-cluster (CC) calculations were not available for certain molecules, at least for some basis sets: no CC calculation was undertaken for the 1-octanol; for CCSD(min-fc) and CCSD(max-fc) *p*-xylene and hexane were missing for aug-cc-pVDZ and Sadlej-pVTZ; for CCSD(full), hexane was only computed with 6-311G(d,p) and aNLO-V, while *p*-xylene was not computed for any basis set with toluene and *o*-dichlorobenzene missing for aug-cc-pVDZ and Sadlej-pVTZ, CCl_4 also unavailable for the latter basis set (CCSD(full)/Sadlej-pVTZ is missing 6 out of 22 molecules, maximum number of missing calculations); CC2(full) calculations.

c. We did not include CCSD(T) in our study, despite it being considered the golden standard for small molecules. The reason for this is that the triple correction is only added perturbatively, and so does not affect anything but the energy and the potential energy surface (i.e., the geometry optimization). In contrast with the long-range dispersion correction, it would have an effect if the second hyperpolarizabilities were computed by the finite-field approach (the numerical differentiation), because the field changes the wavefunction on which the perturbation is applied. In the present study, however, we are using two other methodologies: the response-equation formalism (RE) and the coupled-perturbed Hartree–Fock/Kohn–Sham, with added differentiation to the third order (CPHF/CPKS+FF). For neither of these, the triple correction in CCSD(T) is applicable. We had tried computing the second hyperpolarizability with CCSDT, i.e., the coupled-cluster with the full, non-perturbative triples; unfortunately, computational resources available to us proved to be insufficient for these highly resource-consuming calculations. Therefore, we had to resort to CCSD as the method of highest reputation available, which is known to be inferior to CCSDT and CCSD(T). The latter is the reason we did not specifically label it as the theoretical reference method.

d. To test how the range-separated-hybrid functionals depend on the parameters of the model, we tested combinations of ω, α, and β parameters for the CAM-B3LYP scheme of Ewald split of the two-electron interaction operator:

$$\frac{1}{r_{12}} = \frac{1-(\alpha+\beta\cdot\mathrm{erf}(\omega r_{12}))}{r_{12}} + \frac{\alpha+\beta\cdot\mathrm{erf}(\omega r_{12})}{r_{12}} \tag{1}$$

where ω is the parameter setting the balance between the DFT and the HF-like exchange at intermediate inter-electron distances; α is the percentage of HF-like exchange at zero inter-electron distance, and $\alpha + \beta$ is the percentage of HF-like exchange at the infinite inter-electron distance. We selected values for ω, α and β which are employed in functionals LC-BLYP (ω = 0.47, α = 0.00 and β = 1.00) and CAM-B3LYP (ω = 0.33, α = 0.19 and $\alpha + \beta$ = 0.65), and tested eight combinations of them (see Table S1).

This table is also an example of a design matrix, which was used in this study for constructing factorial experiments. Among all these functionals, we made a special emphasis on testing LC-BLYP(0.33), as was already reported in the literature [43] as performing

Table S1. Design matrix for the range-separated density functionals of the LC-type.

Functional designation	ω	α	$\alpha + \beta$
CAM-B3LYP	0.33	0.19	0.65
CAM-B3LYP(0.47)	0.47	0.19	0.65
LC-BLYP(0.19; 0.33)	0.33	0.19	1.00
LC-BLYP(0.19; 0.47)	0.47	0.19	1.00
CAM-B3LYP(0.00; 0.33)	0.33	0.00	0.65
CAM-B3LYP(0.00; 0.47)	0.47	0.00	0.65
LC-BLYP(0.33)	0.33	0.00	1.00
LC-BLYP	0.47	0.00	1.00

better than both LC-BLYP and CAM-B3LYP; because of this, it was included even in sub-experiments where only a few functionals were present.

e. Basis sets which were not available out-of-the-box in one or both of the software packages were either downloaded from the Basis Set Exchange [136] (Sadlej-pVTZ for Gaussian) or implemented using their descriptions in the original publication (aNLO-V) [17].

f. PElib solvation model used surface tessellation density .NTESS=240 for better quality of results. C-PCM in Dalton 2020 is not implemented for third-order electric properties, including the second hyperpolarizability γ, but is implemented for the first hyperpolarizability β. A comparison between the two methods in calculations of β showed that the ratio of the absolute difference of both solvation models to absolute value for C-PCM dropped exponentially with the increase in the latter value and was at or below 5% for the *o*-dichlorobenzene and nitrobenzene (but asymptotically reached 20–25% at $\gamma \rightarrow 0$; also, acetone was an outlier confirmed by personal communications with Dalton developers). The comparison can be seen in the other parts of the Supplementary information.

g. PCM methods in Gaussian used the default program settings.

h. In our comparisons of different integration grids in Gaussian, we capitalized on the fact that the two Gaussian versions available to us have different settings by default, and the main difference in defaults is the integration grid used: FineGrid (or (75,302) pruned Lebedev grid [137], or spherical harmonics up to 29th order) and UltraFineGrid (or (99,590) pruned Lebedev grid [138], or spherical harmonics up to 41st order). The radial integration accuracy was left at the default level of 10^{-12}. The defaults for grids, however, also use a scheme where higher-order properties utilize progressively coarser grids for efficiency reasons, as is common for quantum chemistry software. As many users still use Gaussian 09 today, we reckoned it would be efficient to use the default settings for these programs, and not specific grid keywords. Hence, one level is Gaussian 09 and another level is Gaussian 16.

i. In our comparisons of different integration grids in Dalton 2020, we used, as one level, the .FINE grid, which is actually even finer than the UltraFineGrid in Gaussian, with .FINE corresponding to the angular expansion order of 42, while UltraFineGrid corresponds to 41. To test the differences here, we have also performed a subexperiment in Dalton with the angular expansion order set to 41 (keyword .ANGINT=41). The FineGrid in Gaussian corresponds to .ANGINT=29. Thus, the three levels used were .FINE, .ANGINT=41 and .ANGINT=29. The radial integration accuracy was left at the default level of 10^{-13}.

j. While Dalton provides a keyword to compute the OKE averaged second polarizability (.IDRI) and we are able to calculate γ in both static and dynamic fashion, in Gaussian we had to select the static value, as it was the closest one in terms of frequency symmetry: $\gamma(0; 0, 0, 0)$ vs. $\gamma(-\omega; \omega, -\omega, \omega)$. There are some dynamic values available from the Gaussian calculations but they are of different frequency symmetry (the direct current Kerr effect

has $\gamma(-\omega; \omega, 0, 0)$ and the electric-field-induced second harmonic generation has $\gamma(-2\omega; \omega, \omega, 0)$). All values used in our analysis were the average γ parallel to the incoming electric field.

k. When the preparation of this manuscript was in its last stage, we discovered that actually the .IDRI keyword seems to not be defined correctly, as not only multiple components are computed redundantly but also many components are not computed at all; however, as we can judge from the calculations with CPKS+FF, all of these missing components are at least one order of magnitude smaller in their absolute value than all the key components, and all of these are computed. This subject requires further investigation; however, we consider it acceptable to still present the results of this part of the present study.

In the part of the study regarding the geometry utilized in the calculations, we only used 12 selected compounds (chloroform, acetonitrile, acetone, p-xylene, o-dichlorobenzene, pyridine, toluene, dichloromethane, ethanol, methanol, and nitrobenzene, which mainly cover the structural diversity of our set). We used ten methods for geometry optimization: two-electron Hamiltonians B3LYP, B3LYP-D3BJ (B3LYP with Grimme's dispersion correction [139,140] with Becke—Johnson's damping [141]), M06-2X, ωB97XD and frozen-core MP2, all with 6-311G(d,p) basis set, in vacuum or with C-PCM. All geometry optimizations were performed with method-precise force constants (full Hessian computation) at the final stage of optimization. An exception is MP2(fc), as numerical force constants are rather costly; we, however, re-optimized the geometries in solution with the full Hessian, and the values of a selected subset of second hyperpolarizabilities (LC-BLYP with four basis sets) did not change more than by 0.16%. In this dataset, we used five levels of the exchange-correlation Hamiltonian (CAM-B3LYP, LC-BLYP, LC-BLYP(0.33), M06-2X, and HF), five levels of basis sets. The results for both Gaussian and Dalton can be seen in the Fig. S1.

In the analysis of data, we found that there is little difference in γ values depending on which density functional was used for the geometry optimization and on whether solvent-related terms were included in the Hamiltonian for geometry optimization (and when there were differences, they were inconsistent across the computational space). Overall, though, the leader is the M06-2X density functional, rather closely followed by the ωB97XD functional. As we were going to make calculations on hydrogen-bonded clusters as well, we still deemed it to be more appropriate if we used the latter method, as M06-2X, though providing a good description of short- and middle-range Van der Waals interactions, can easily fail for the long ones. Therefore, we selected ωB97XD/6-311G(d,p)/C-PCM as the geometry optimization method to reduce the total computation time and to make the rest of data more comprehensible (just like we found to be done in other studies [7,8,12,16–21]). Another notable conclusion is that the B3LYP with D3BJ dispersion correction does not provide good geometries for the calculations of the second hyperpolarizability, as opposed to the popular belief about its ability to describe the non-covalent interactions.

We have also compared the RMSD of atomic positions for all the geometries used, which we computed using UCSF Chimera 1.15 [142,143]; before calculation, the structures were rotated and translated to obtain the best match of atomic positions. In the vast majority of cases, the root-mean-square differences between the geometries did not exceed 0.01 Å. The three cases where the performance of functionals was actually different were acetone, p-xylene, and nitrobenzene. In acetone, the dihedral between the methyl groups varied from almost eclipsed (107° for M06-2X) to the one-third towards the staggered conformation (89.5° for ωB97XD), and the difference between the dihedral values between M06-2X and the next closest functional (B3LYP) was twice as large as the next largest difference among the ordered data. In p-xylene, the dihedral between the methyl groups was 60.6–60.7°

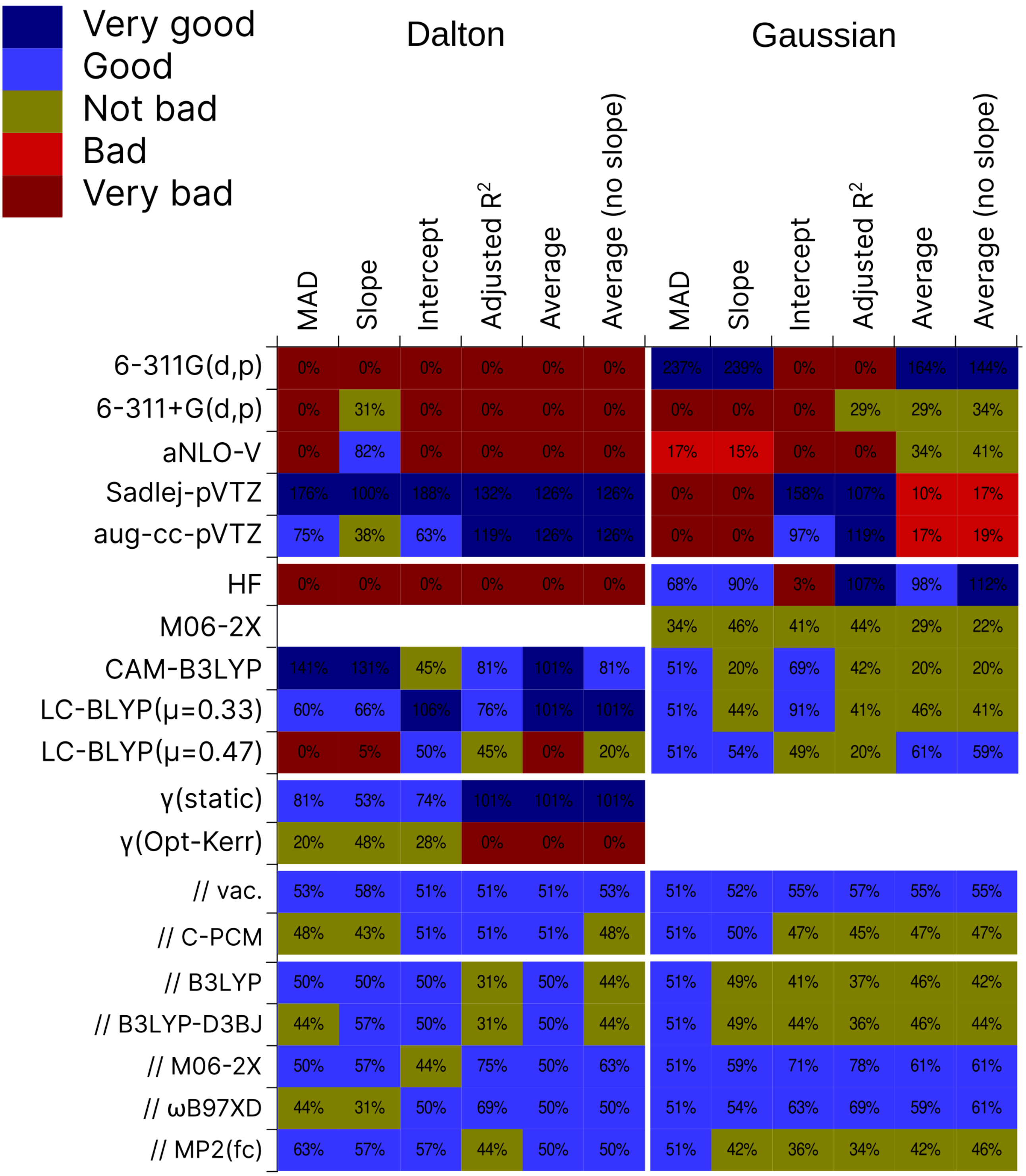

Figure S1. The results of the test set of the geometry optimization method, excluded from the main study.

for all methods except for M06-2X, which produced 47.1°. In the nitrobenzene, however, the largest difference was shown by MP2(fc), which yielded the dihedral angle of 20.6° between the nitro group and neighboring ring atoms, while all the other methods predicted

co-planarity of both moieties. To test the impact of the changes in geometry further, we studied the dependence of γ on the dihedral angle between the benzene and nitro group planes (as it was the largest difference and also directly affecting the π conjugation); the resulting relation is quite weak at dihedral values below 20° for all methods (M06-2X, CCSD and CAS-SCF were not tested) and until about 40–50° for CAM-B3LYP, LC-BLYP (μ=0.33) and BH&HLYP; the data can be found in the Supporting information spreadsheets. Also, average computation time (on identical nodes) did not change substantially for geometries computed either with MP2(fc) or with ωB97XD.

The functional we chose for optimizing the geometry contains long-range dispersion energy correction (or simply dispersion correction) included. However, we did not include the presence of dispersion correction in the Hamiltonian in the list of computational factors despite its importance in many quantum chemical calculations. The reason for this is that the dispersion correction is an additive term for the full single-point energy, dependent solely on the positions of the atomic nuclei [144]. Importantly, however, it does not affect the wavefunction or electron density, hence it has no impact on the response properties [145]. The long-range dispersion correction should therefore have no impact on the second hyperpolarizability value even if it was computed with the finite-field methodology. The reason for this is that the geometry of the molecule does not change during the computation, hence the dispersion term remains constant regardless of the applied field strength. Therefore, this constant term will disappear during the numerical differentiation.

3. The Conversion Between the Experimental and the Calculated Data

To calculate values for the real part of the third-order susceptibility of our materials, we used the following equation presented in the literature [146]:

$$\chi_{\Re}^{(3)}\left[\frac{\mathrm{m}^2}{\mathrm{V}^2}\right] = \frac{4\cdot\varepsilon_0\cdot c\cdot n_0^2}{3}\cdot n_2\left[\frac{\mathrm{m}^2}{\mathrm{W}}\right] \tag{2}$$

where ε_0 is the vacuum dielectric constant, c is the speed of light in vacuum, and n_0 is the linear refractive index. To convert the real part of third-order susceptibility from SI to the Gaussian (esu) system, the following relation was used [147]:

$$\chi_{\Re}^{(3)}[\mathrm{esu}] = \frac{9\cdot 10^8}{4\pi}\cdot\chi_{\Re}^{(3)}\left[\frac{\mathrm{m}^2}{\mathrm{V}^2}\right]. \tag{3}$$

To better compare NLO properties on the molecular scale, we calculated values for second-order hyperpolarizability of our materials using the following expression [148]:

$$\gamma[\mathrm{esu}] = \frac{\chi_{\Re}^{(3)}[\mathrm{esu}]}{\left(\frac{1}{3}(n_0^2+2)\right)^4\cdot N} \tag{4}$$

where N is the molecule concentration per cm^3. Values for the density and linear refraction index were taken from multiple sources with efforts to ensure consistency (mostly via PubChem [149]; see the other parts of the Supplementary information for details).

Different conventions can be used to define third-order susceptibility, usually differing by *(i)* inclusion of Taylor series‘ coefficient of $1/(3!)$ or lack thereof; *(ii)* inclusion of coefficients from the field dependence expansion in combinations of static and dynamic fields [150–153]. For calculated values, both coefficients are explicitly not included in the value of γ (because this is the most fundamental way possible, with convergence to zero frequency); this is named "T" [150] or "IV" [151] convention. For experimental values, usually all coefficients are implicitly absorbed into γ because these values usually are not purely electronic third-order polarizability; this is named "X" [150] or "I" [151] convention. To convert values between these conventions, the following equations need to be used:

$$\begin{cases} \chi_{\mathrm{exp}}^{(3)}(-\omega;\omega,\omega,-\omega) = \frac{1}{8}\cdot\chi_{\mathrm{QCC}}^{(3)}(-\omega;\omega,\omega,-\omega) \\ \chi_{\mathrm{exp}}^{(3)}(-\omega;\omega,0,0) = \frac{1}{2}\cdot\chi_{\mathrm{QCC}}^{(3)}(-\omega;\omega,\omega,-\omega) \\ \chi_{\mathrm{exp}}^{(3)}(-2\omega;\omega,\omega,0) = \frac{1}{4}\cdot\chi_{\mathrm{QCC}}^{(3)}(-2\omega;\omega,\omega,0) \\ \chi_{\mathrm{exp}}^{(3)}(0;0,0,0) = \frac{1}{6}\cdot\chi_{\mathrm{QCC}}^{(3)}(0;0,0,0) \end{cases} \tag{S5}$$

4. Factorial Design of the Study

In total, we defined and processed the following data subsets:

1. Hamiltonians and basis sets in Dalton, CC methods included (15 levels of Hamiltonian, 5 levels of basis sets).

2. Hamiltonians and basis sets in Dalton, CC methods included (15 levels of Hamiltonian, 5 levels of basis sets), dynamic values of γ.

3. Hamiltonians, basis sets, and static vs. dynamic values in Dalton, CC methods included (15 levels of Hamiltonian, 5 levels of basis sets, 2 levels of static vs. dynamic).

4. Hamiltonians and basis sets in Dalton, CC methods excluded (11 levels of Hamiltonian, 8 levels of basis sets).

5. Hamiltonians and basis sets in Gaussian 16, meta-global-hybrid functionals included (15 levels of Hamiltonian, 8 levels of basis sets).

6. Hamiltonians and basis sets in Gaussian 16 and Dalton, using UltraFineGrid and .FINE grids (11 levels of Hamiltonian, 8 levels of basis sets, 2 levels of CPKS+FF vs. RE).

7. Hamiltonians and basis sets in Gaussian 09 and Dalton, using FineGrid and .ANGINT=29 grids (5 levels of Hamiltonian, 5 levels of basis sets, 2 levels of CPKS+FF vs. RE).

8. Range-separated-hybrid density functionals only, (8 levels of Hamiltonian, 8 levels of basis sets, 2 levels of CPKS+FF vs. RE).

9. Range-separated-hybrid density functionals only in Gaussian 16 (8 levels of Hamiltonian, 8 levels of basis sets).

10. Range-separated-hybrid density functionals only in Dalton (8 levels of Hamiltonian, 8 levels of basis sets).

11. Hamiltonians, basis sets and integration grid in Gaussian (6 levels of Hamiltonian, 5 levels of basis sets, 2 levels of Gaussian 09 (FineGrid) vs. Gaussian 16 (UltraFineGrid)).

12. Hamiltonians, basis sets and integration grid in Dalton, set 1 (8 levels of Hamiltonian, 5 levels of basis sets, 2 levels of .FINE vs. .ANGINT=29).

13. Hamiltonians, basis sets and integration grid in Dalton, set 2 (5 levels of Hamiltonian, 8 levels of basis sets, 2 levels of .FINE vs. .ANGINT=29).

14. Hamiltonians, basis sets and integration grid in Dalton, set 3 (3 levels of Hamiltonian, 5 levels of basis sets, 3 levels of .FINE vs. .ANGINT=29 vs. .ANGINT=41).

15. Hamiltonians, basis sets and solvation presence in the Hamiltonian, in Dalton, set 1 (8 levels of Hamiltonian, 5 levels of basis sets, 2 levels of vacuum vs. PElib).

16. Hamiltonians, basis sets and solvation presence in the Hamiltonian, in Dalton, set 2 (5 levels of Hamiltonian, 8 levels of basis sets, 2 levels of vacuum vs. PElib).

17. Hamiltonians, basis sets and solvation presence in the Hamiltonian, in Gaussian 16, set 1 (12 levels of Hamiltonian, 5 levels of basis sets, 2 levels of vacuum vs. C-PCM).

18. Hamiltonians, basis sets and solvation presence in the Hamiltonian, in Gaussian 16, set 2 (5 levels of Hamiltonian, 8 levels of basis sets, 2 levels of vacuum vs. C-PCM).

19. Hamiltonians, basis sets and solvation model type in the Hamiltonian, in Gaussian 16 (6 levels of Hamiltonian, 5 levels of basis sets, 3 levels of C-PCM vs. IEF-PCM vs. SMD).

20. Hamiltonians, basis sets and solvation presence in the Hamiltonian, in Gaussian 16, set 2 (5 levels of Hamiltonian, 8 levels of basis sets, 2 levels of vacuum vs. IEF-PCM); this subset was added *post hoc* because of the results for subsets 18 and 19.

Each of these subsets was analyzed separately, but conclusions were drawn in connection with the results from other subsets.

Data normality. To assess the applicability of parametric statistical methods, we employed the Shapiro–Wilk normality test for every subset of the data (see Table 2). Interestingly, the number of apparently non-normal ($p < 0.05$) data series in all subsets never exceeds 5%, sans the subsets which contain either CC data series or τ-meta density functional data series (in these cases, the number of non-normal data series can even exceed 20%). Specifically, about 40% of CC data series and about 50% or more of τ-meta functional data series are not normally distributed. As comparing these Hamiltonians with other methods is one of the most important tasks of the present study, we decided to forego using the conventional parametric research methods and use the non-parametric linear regression (Kendall–Theil–Sen regression [154,155] in Siegel's [156] repeated median formulation). The main idea of such a regression is to take the median over all the possible slopes and (separately) all intercepts of the straight lines connecting all points in the 2D subset. Such a measure is not dependent in any way on the assumed distribution of the data; however, it also cannot provide the confidence intervals, only the p-value [157].

Table S2. Proportion of the non-normal data series among the datasets.

Dataset	Proportion of the non-normally distributed data series	Proportion of CC, τ-meta or other data series among the series which are not normally distributed	Proportion of CC or τ-meta data series which are not normally distributed
1	13%	67% (CC), 67% (6-311G(d,p)), 10% (GH)	35% (CC)
2	16%	75% (CC), 8% (GH)	45% (CC)
3	15%	75% (CC), 9% (GH)	40% (CC)
4	3%	33% (GH), no τ-meta or CC	—
5	21%	80% (τ-meta), 8% (GH)	63% (τ-meta)
6	4.5%	38% (GH), no τ-meta or CC	—
7	2%	100% (GH), no τ-meta or CC	—
8	4%	(all tested are RSH, no τ-meta or CC)	—
9	4.5%	(all tested are RSH, no τ-meta or CC)	—
10	3%	(all tested are RSH, no τ-meta or CC)	—
11	8%	100% (τ-meta)	50% (τ-meta)
12	4%	100% (GH), no τ-meta or CC	—
13	0%	no τ-meta or CC	—
14	0%	no τ-meta or CC	—
15	2.5%	100% (GH), no τ-meta or CC	—
16	1%	no τ-meta or CC	—
17	22%	93% (τ-meta), 7% (GH)	60% (τ-meta)
18	0%	all tested are RSH, no τ-meta or CC	—
19	9%	75% (τ-meta), 25% (GH)	40% (τ-meta)
20	10%	83% (τ-meta), 17% (GH)	50% (τ-meta)

5. Definition of Linear Contrasts

To evaluate the performance of the specific traits, we calculated linear contrasts [158]. The statistical definition starts with the model, which we (for the sake of simplicity) present here for the case of two factors:

$$y_{ij} = \mu + f_i + b_j + \varepsilon_{ij}, \tag{S6}$$

where y_{ij} is the value of a descriptor at the level i of the factor f and level j of the factor b;

μ is the overall average of descriptor values (over the whole population of possible values of this descriptor);

f_i is the influence of the factor f at the level i on the descriptor value;

b_j is the influence of the factor b at the level j on the descriptor value;

ε_{ij} is the unexplained deviation of the descriptor value from the systematic influence of the factors f and b.

We assumed no interactions between the factors f and b here. Then, the analysis of variance (ANOVA) identity is:

$$SS_{total} = S_{ST} + S_{SR}, \tag{S7}$$

where SS_{total} is the total sum of the squares of deviations from the sample mean;

SS_T is the part of the total sum explained by the effect of treatments;

SS_R is the residual sum of squares, containing all the unexplained effects within the present model.

By expanding this equation, we obtain:

$$\sum_{i,j}(y_{ij} - y_{..})^2 = \sum_i (y_{i.} - y_{..})^2 + \sum_j (y_{.j} - y_{..})^2 + \sum_{i,j}(y_{ij} - y_{i.} - y_{.j} + y_{..})^2, \tag{S8}$$

where $y_{..}$ is the overall average value of the descriptor (over the sample used in the experiment);

$y_{i.}$ is the average for all values of the descriptor which were observed for the level i of the factor f;

$y_{.j}$ is the average for all values of the descriptor which were observed for the level j of the factor b.

Now, the estimated effect of applying a specific treatment (i.e., a combination of levels of multiple factors) can be expressed as:

$$t_j = y_{.j} - y_{..}, \tag{S9}$$

We can define a general treatment comparison as a linear contrast of treatment effects:

$$L_k = \sum_j l_{jk} t_j = \sum_j l_{jk} y_{.j}, \tag{S10}$$

where $\sum_j l_{ik} = 0$ and the second equality holds for the estimate of the contrast L_k.

It can be shown, then, that the sum of the squared deviations for the specific contrast L_k can be calculated as:

$$SS(L_k) = \frac{\left(\sum_j l_{jk} n_j y_{.j}\right)^2}{\sum_j l_{jk}^2 n_j}, \tag{S11}$$

where n_j is the number of observations for the level j of the factor b.

For p levels of the factor b, we can define $(p-1)$ linear contrasts, each having its own sum of squares $\mathrm{SS}(L_k)$. The residual sum of squares SS_R is then defined as the difference between the global sum of squares and the sum of all the contrast sums of squares:

$$SS_R = SS_T - \sum_k SS(L_k). \tag{S12}$$

Now we can use the F test to determine if there is any significance in a particular contrast L_k. For example, suppose we have 5 basis sets: 6-311G(d,p), 6-311+G(d,p), aug-cc-pVDZ, aNLO-V, and Sadlej-pVTZ. For these, we can define a contrast L_1 that checks whether the basis set is specifically tailored for the calculation of polarizabilities. The contrast coefficients would be $\vec{l}_1 = (-2,\ -2,\ -2,\ +3,\ +3)$.

By carrying out the F test, we can evaluate the probability p-value indicating the likelihood that this contrast has no statistical significance. Let us say we obtain $p = 0.73$ (marginal significance at the confidence level of 95% or 0.95). This would imply that simply choosing any special basis set does not necessarily yield better results in the specific descriptor (e.g., the slope) of the correlation between the calculated and experimental second hyperpolarizabilities.

On the other hand, we can define another contrast L_2 to test whether triple-zeta basis sets are generally better than double-zeta ones. Then the coefficients would be $\vec{l}_2 = (+3,\ +3,\ -2,\ -2,\ -2)$, and the result of an F test would be $p = 0.04$ (significant if the confidence level is 0.95).

This, in its turn, means that the value of this particular descriptor is indeed systematically influenced using either triple-zeta or double-zeta basis sets. However, the low p-value *does not automatically mean that the effect of this contrast would be positive*; it just means the effect is significant. Whether using triple-zeta basis sets brings in a positive effect can be determined by looking at the value of the L_2 itself: if it is positive, then we should rather use triple-zeta basis sets; if L_2 is negative, we would recommend using double-zeta basis sets instead. Regarding the L_1, we cannot say whether it is worth using a non-specified tailored basis set at all.

6. Additional Data on Computation Time

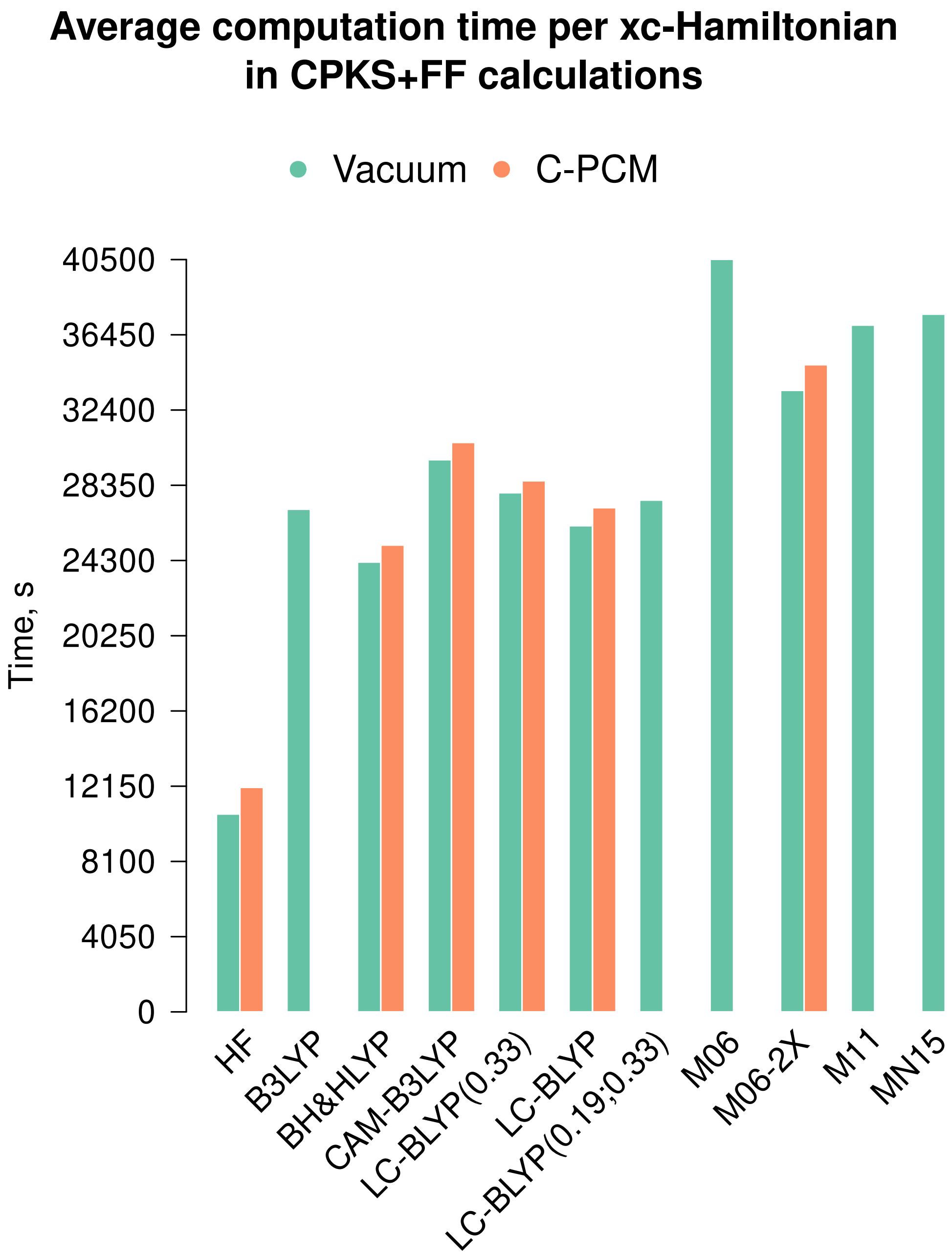

Figure S2. Basis-set dependence.

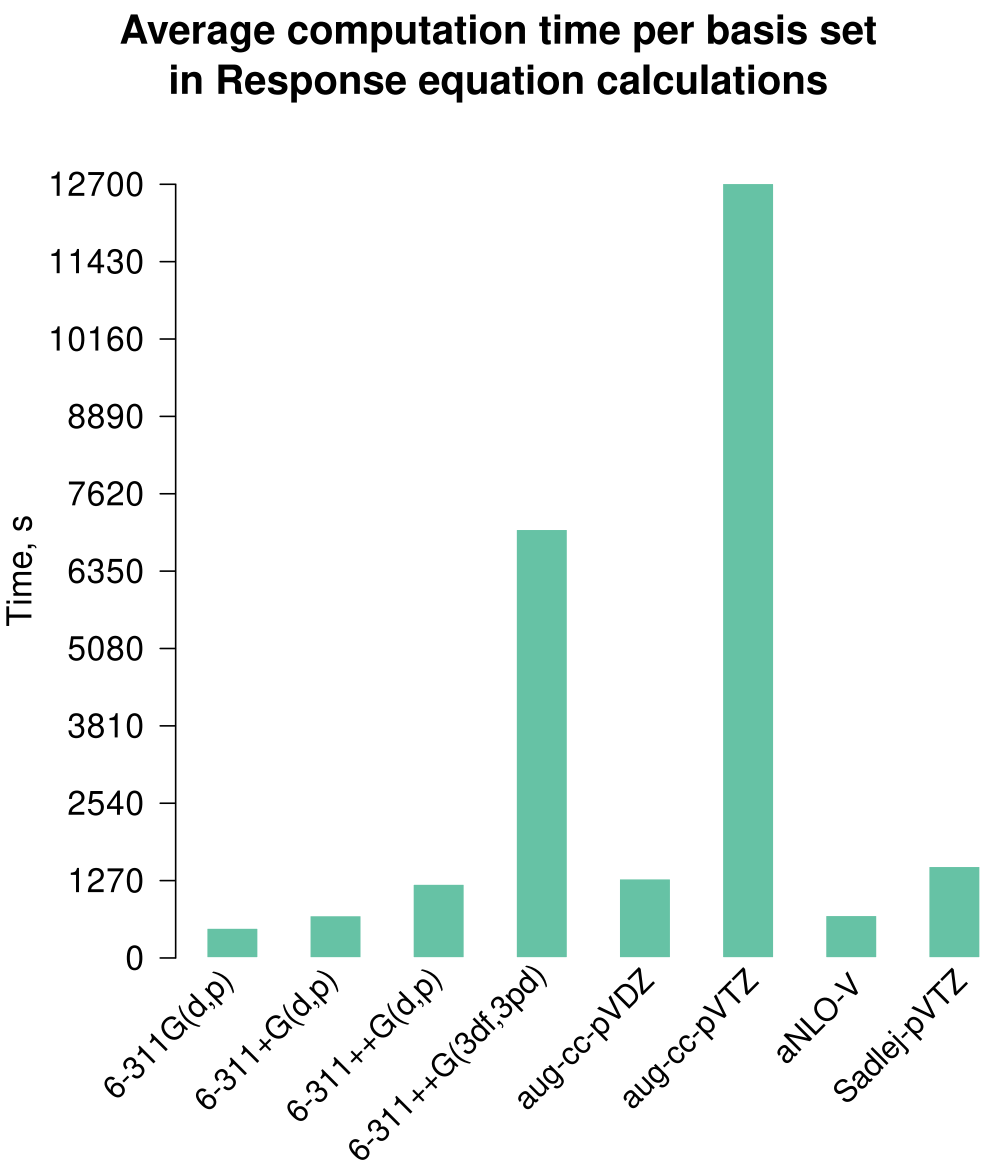

Figure S3. Basis-set dependence.

7. Popularity of Quantum Chemical Computational Software for Calculations of the Second Hyperpolarizabilities

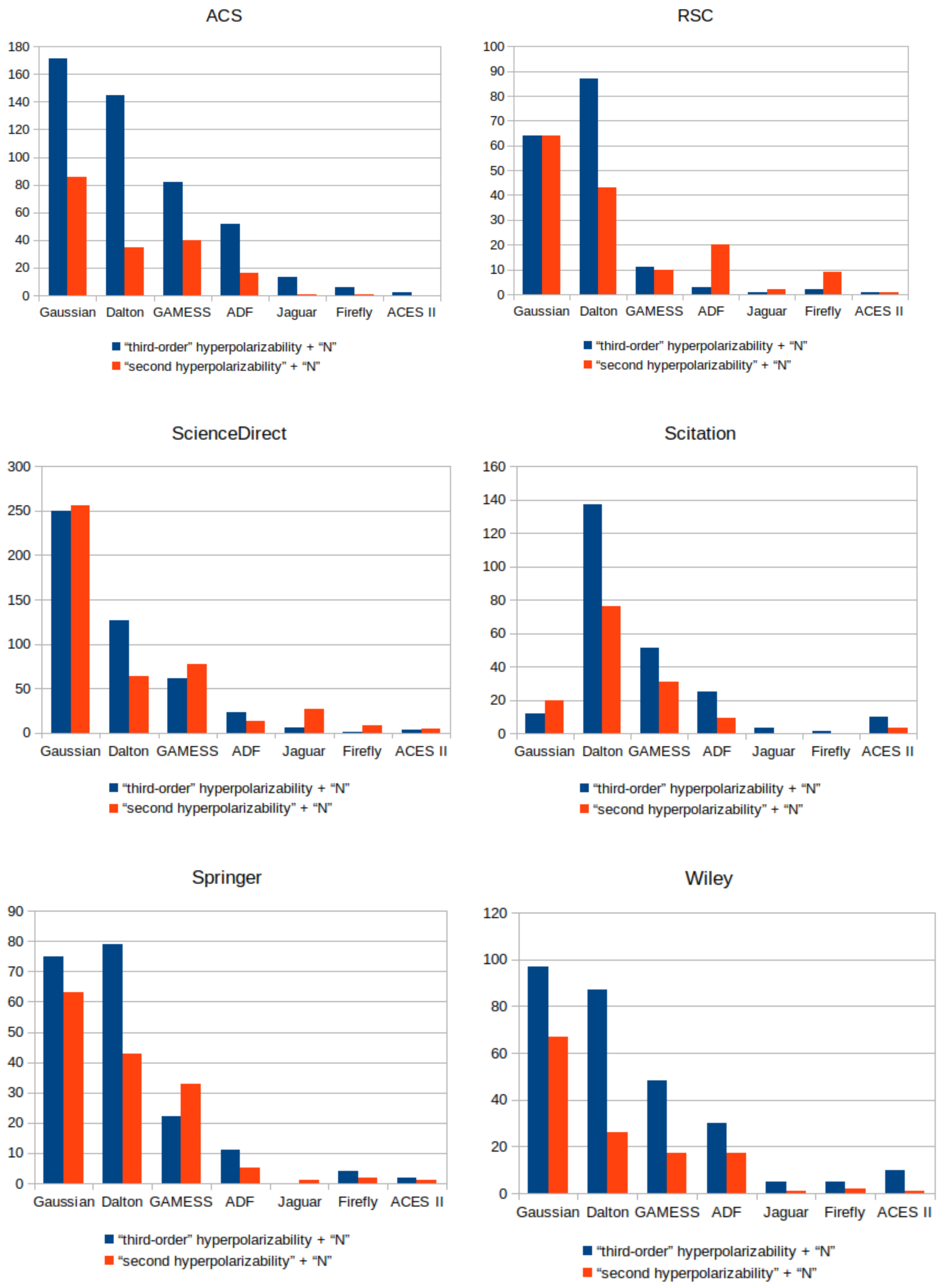

Figure S4. Popularity of quantum chemical computational software for calculations of the second hyperpolarizabilities.

8. Popularity of Two-Electron Hamiltonians for Calculations of the Second Hyperpolarizability

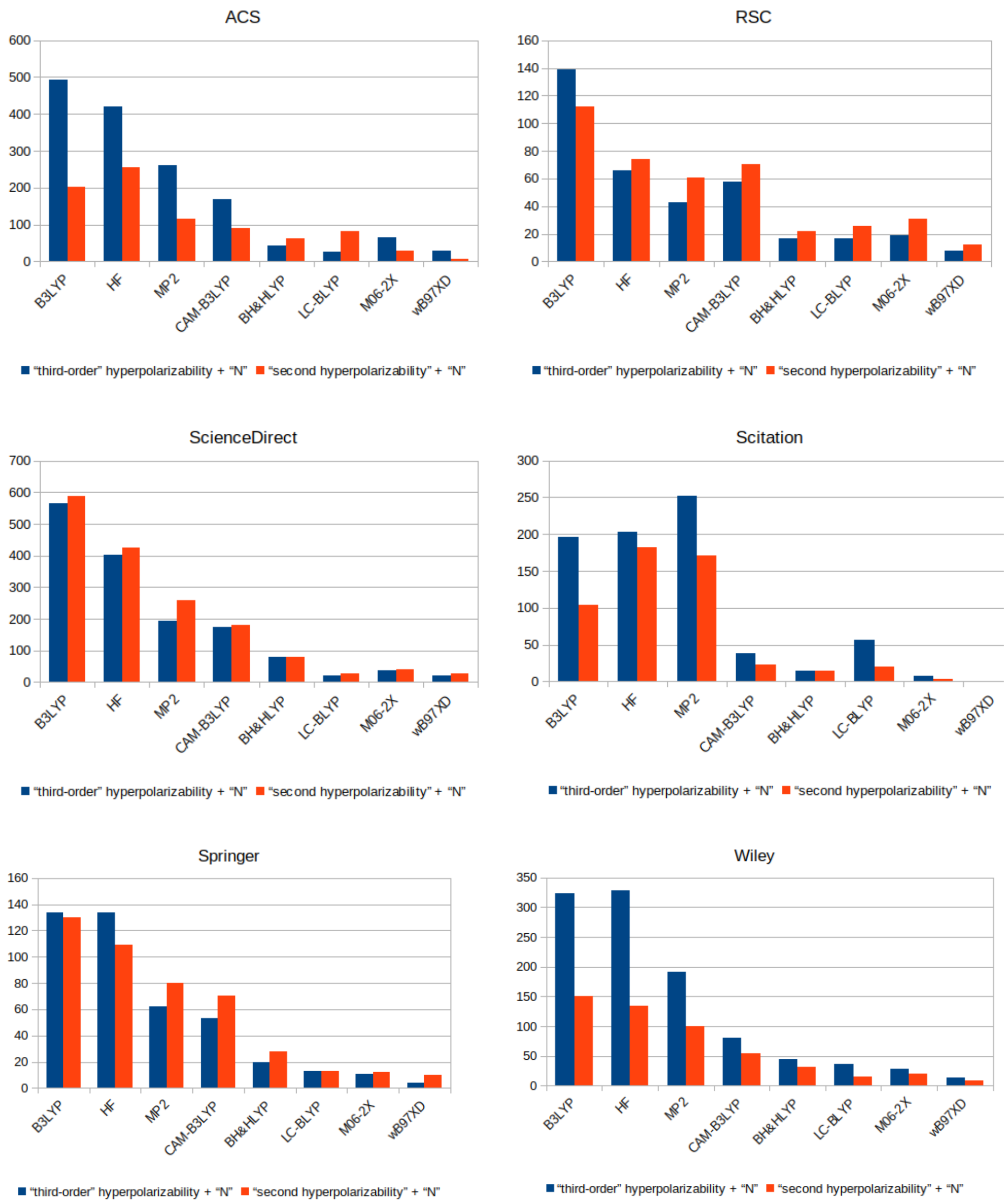

Figure S5. Popularity of two-electron Hamiltonians for calculations of the second hyperpolarizability.

9. Composition of Different Basis Sets

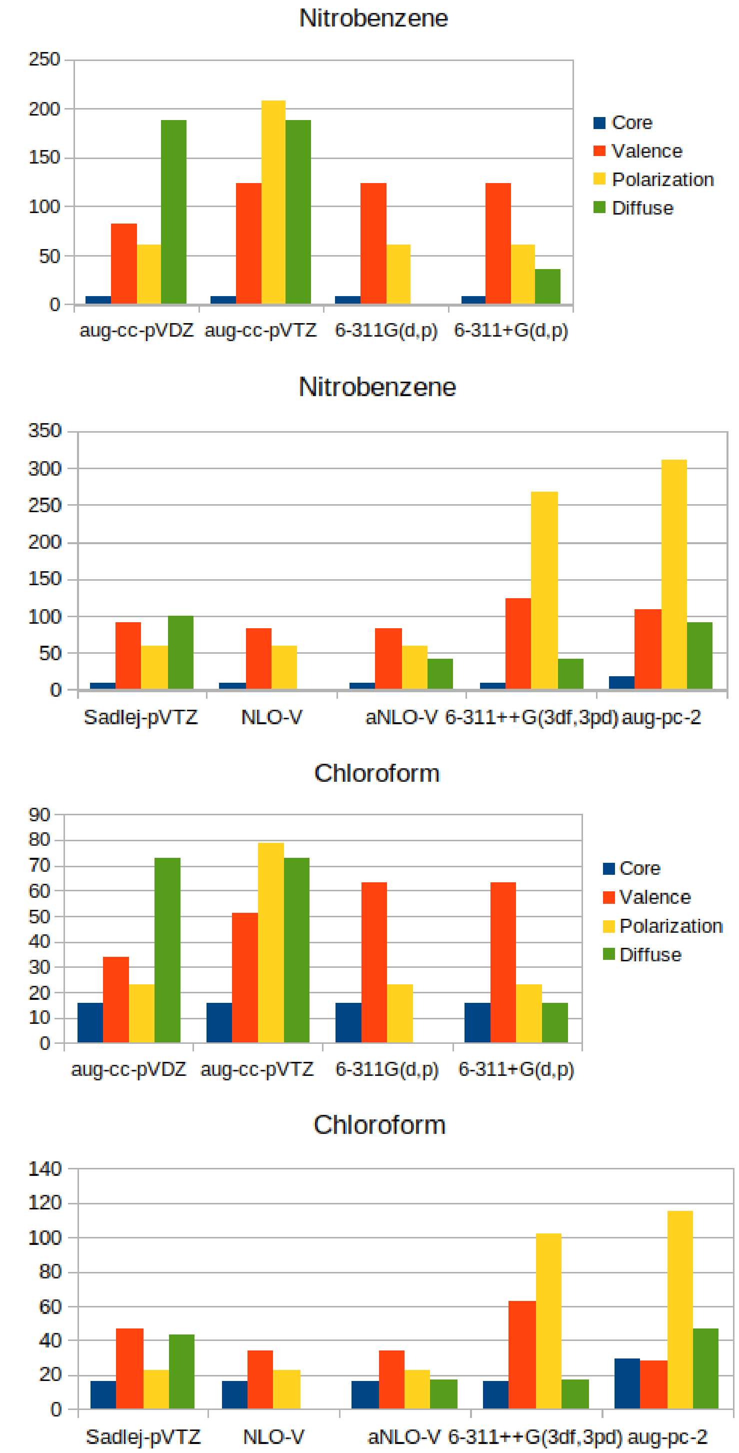

Figure S6. Number of functions of different types for several basis sets, including those used in the present study, for two representative molecules.

10. Calculations of One-Photon Electronic Spectra of Selected Compounds

One-photon absorption (1PA) spectra are calculated with the same functionals as the second hyperpolarizability and the 6-311+G(d,p) basis set. They are presented both in the linear-response and the self-consistent PCM formalisms (in the last one, molecular orbitals are modified at each iteration; following Marenich et al.'s convention [159], we use the designation IBSF for it, after the authors' initials). As can be seen, the first absorption

Table S3. Excitation energies (in nm) obtained using linear-response and state-specific (IBSF) TD-DFT/TD-HF methods for selected compounds.

Compound	Transition	Linear-response TD-DFT/TD-HF					State-specific (IBSF) TD-DFT/TD-HF				
		CAM-B3LYP	HF	M06-2X	LC-BLYP (μ=0.47)	LC-BLYP (μ=0.33)	CAM-B3LYP	HF	M06-2X	LC-BLYP (μ=0.47)	LC-BLYP (μ=0.33)
**D**	$S_1 \leftarrow S_0$	238	219	235	231	239	236	215	233	230	237
	$S_2 \leftarrow S_0$	215	219	210	210	212	212	215	207	207	209
**N**	$S_1 \leftarrow S_0$	317	260	321	310	323	324	255	316	305	319
	$S_2 \leftarrow S_0$	281	242	285	273	281	312	240	282	270	279
	$S_1 \leftarrow S_0$	326	319	344	347	342	326	319	344	347	342
**CS_2**	$S_2/S_3 \leftarrow S_0$	303	298	305	314	312	301	296	304	313	310
	$S_4 \leftarrow S_0$	199	191	199	198	202	187	179	187	185	189

maximum of CS2 is much closer to 400 nm than that of the nitrobenzene, hence CS2 should be described better by our calculations (but the opposite is observed).

Table S4. TD-DFT computed excitation wavelengths and oscillator strengths (f) for CS_2, DMSO, and nitrobenzene.

CS_2	DMSO	Nitrobenzene
337.20 nm, f = 0.0000	222.07 nm, f = 0.0044	313.25 nm, f = 0.0000
309.03 nm, f = 0.0000	202.17 nm, f = 0.0613	272.67 nm, f = 0.0005
309.03 nm, f = 0.0000	196.10 nm, f = 0.0284	260.60 nm, f = 0.0374
202.40 nm, f = 1.2670		250.67 nm, f = 0.3219
		200.80 nm, f = 0.0071

Only transitions above $\approx$ 200 nm are shown. LC-BLYP(0.33)/Sadlej-pVTZ, TD-LR, SMD.

Abbreviations

The following abbreviations are used in this manuscript:

a.u.	Atomic Units
C-PCM	Conductor-like Polarizable Continuum Model
CC2	Coupled-Cluster Singles and Approximate Doubles
CCSD	Coupled-Cluster Singles and Doubles
CPHF	Coupled-Perturbed Hartree–Fock
CPKS	Coupled-Perturbed Kohn–Sham
DFT	Density Functional Theory
DMSO	DiMethyl SulfOxide
DMFA	*N*, *N*-DiMethylFormAmide
FF	Finite Field
FFD	Full Factorial Design
GH	Global-Hybrid [density functionals]
HF	Hartree–Fock
IEF-PCM	Integral Equation Formalism PCM
MAD	Mean Absolute Deviation
MGH	τ-Meta-Global-Hybrid [density functionals]
MP2	Møller–Plesset Perturbation Theory (second-order)
OKE	Optical Kerr effect
PCM	Polarizable Continuum Model
RE	Response Equation
RSH	Range-Separated-Hybrid [density functionals]
SMD	Solvation Model based on Density
SOS	Sum-Over-States
TDDFT	Time-Dependent Density Functional Theory

References

1. Li, H.P.; Shen, X.P.; Han, K.; Tang, G. Solvent Effects on Polarizability and Hyperpolarizability of Spirobifluorene Derivative. *J. Mol. Model.* **2014**, *20*, 2126. https://doi.org/10.1007/s00894-014-2126-7.
2. Hatua, K.; Mondal, A.; Nandi, P.K. Static Second Hyperpolarizability of Inverse Sandwich Compounds (M_1–C_5H_5–M_2) of Alkali (M_1 = Li, Na, K) and Alkaline Earth Metals (M_2 = Be, Mg, Ca). *Phys. Chem. Chem. Phys.* **2018**, *20*, 13331–13339. https://doi.org/10.1039/C8CP01210E.
3. Suponitsky, K.Y.; Tafur, S.; Masunov, A.E. Applicability of Hybrid Density Functional Theory Methods to Calculation of Molecular Hyperpolarizability. *J. Chem. Phys.* **2008**, *129*, 044109. https://doi.org/10.1063/1.2936121.
4. Brandão, I.; Fonseca, T.L.; Franco, L.R.; Georg, H.C.; Castro, M.A. Applicability of DFT Functionals for Evaluating the First Hyperpolarizability of Phenol Blue in Solution. *J. Chem. Phys.* **2021**, *154*, 094501. https://doi.org/10.1063/5.0033134.
5. Labidi, S.N.; Kanoun, M.B.; De Wergifosse, M.; Champagne, B. Theoretical Assessment of New Molecules for Second-Order Nonlinear Optics. *Int. J. Quantum Chem.* **2011**, *111*, 1583–1595. https://doi.org/10.1002/qua.22757.
6. Xu, H.L.; Sun, S.L.; Muhammad, S.; Su, Z.M. Three-Propeller-Blade-Shaped Electride: Remarkable Alkali-Metal-Doped Effect on the First Hyperpolarizability. *Theor. Chem. Acc.* **2011**, *128*, 241–248. https://doi.org/10.1007/s00214-010-0837-0.
7. Shalin, N.I.; Fominykh, O.D.; Balakina, M.Y. Effect of Acceptor Moieties on Static and Dynamic First Hyperpolarizability of Azobenzene Chromophores. *Chem. Phys. Lett.* **2019**, *717*, 21–28. https://doi.org/10.1016/j.cplett.2018.12.045.
8. Lu, S.I.; Chiu, C.C.; Wang, Y.F. Density functional theory calculations of dynamic first hyperpolarizabilities for organic molecules in organic solvent: Comparison to experiment. *J. Chem. Phys.* **2011**, *135*, 134104. https://doi.org/10.1063/1.3644336.
9. Marder, S.R.; Gorman, C.B.; Tiemann, B.G.; Perry, J.W.; Bourhill, G.; Mansour, K. Relation Between Bond-Length Alternation and Second Electronic Hyperpolarizability of Conjugated Organic Molecules. *Science* **1993**, *261*, 186–189. https://doi.org/10.1126/science.261.5118.186.
10. Zhu, W.; Wu, G.S.; Jiang, Y. Incorporation of Solvent Effects into Density Functional Predictions of Molecular Polarizabilities and Hyperpolarizabilities. *Int. J. Quantum Chem.* **2002**, *86*, 347–355. https://doi.org/10.1002/qua.10029.
11. Hrivnák, T.; Medved', M.; Bartkowiak, W.; Zaleśny, R. Hyperpolarizabilities of Push–Pull Chromophores in Solution: Interplay between Electronic and Vibrational Contributions. *Molecules* **2022**, *27*, 8738. https://doi.org/10.3390/molecules27248738.
12. Lu, S.I. Computational Study of Static First Hyperpolarizability of Donor–Acceptor Substituted (E)-Benzaldehyde Phenylhydrazone. *J. Comput. Chem.* **2011**, *32*, 730–736. https://doi.org/10.1002/jcc.21667.

13. Manzoni, V.; Coutinho, K.; Canuto, S. An Insightful Approach for Understanding Solvatochromic Reversal. *Chem. Phys. Lett.* **2016**, *655–656*, 30–34. https://doi.org/10.1016/j.cplett.2016.05.028.
14. Castet, F.; Champagne, B. Assessment of DFT Exchange–Correlation Functionals for Evaluating the Multipolar Contributions to the Quadratic Nonlinear Optical Responses of Small Reference Molecules. *J. Chem. Theory Comput.* **2012**, *8*, 2044–2052. https://doi.org/10.1021/ct300174z.
15. Brandão, I.; Franco, L.R.; Fonseca, T.L.; Castro, M.A.; Georg, H.C. Confirming the Relationship Between First Hyperpolarizability and the Bond Length Alternation Coordinate for Merocyanine Dyes. *J. Chem. Phys.* **2017**, *146*, 224505. https://doi.org/10.1063/1.4985672.
16. Wang, C.; Yuan, Y.; Tian, X.; Sun, J.; Yuan, J. The effects of exact exchange of density functionals on the evaluation of second hyperpolarizabilities of streptocyanines using sum-over-states method. *Comput. Theor. Chem.* **2016**, *1085*, 40–45. https://doi.org/10.1016/j.comptc.2016.04.006.
17. Paschoal, D.; Dos Santos, H.F. Assessing the Quantum Mechanical Level of Theory for Prediction of Linear and Nonlinear Optical Properties of Push-Pull Organic Molecules. *J. Mol. Model.* **2013**, *19*, 2079–2090. https://doi.org/10.1007/s00894-012-1644-4.
18. Li, H.P.; Shen, X.P.; Han, K.; Tang, G.; Zhang, Z.H. Quantum Chemistry Study on the Third-Order Nonlinear Optical Properties of Spirobifluorene Derivatives. *Comput. Theor. Chem.* **2013**, *1023*, 95–98. https://doi.org/10.1016/j.comptc.2013.09.016.
19. Mondal, A.; Hatua, K.; Roy, R.S.; Nandi, P.K. Successive Lithiation of Acetylene, Ethylene and Benzene: A Comprehensive Computational Study of Large Static Second Hyperpolarizability. *Phys. Chem. Chem. Phys.* **2017**, *19*, 4768–4777. https://doi.org/10.1039/C6CP07845A.
20. Banerjee, P.; Nandi, P.K. Hydrides, Alkalides, and Halides of Calcium Metal Chain: Electronic Structure and NLO Property. *Struct. Chem.* **2018**, *29*, 859–870. https://doi.org/10.1007/s11224-017-1069-x.
21. Xu, L.; Kumar, A.; Wong, B.M. Linear Polarizabilities and Second Hyperpolarizabilities of Streptocyanines: Results from Broken-Symmetry DFT and New CCSD(T) Benchmarks. *J. Comput. Chem.* **2018**, *39*, 2350–2359. https://doi.org/10.1002/jcc.25519.
22. Champagne, B.; Perpète, E.A. Bond Length Alternation Effects on the Static Electronic Polarizability and Second Hyperpolarizability of Polyacetylene Chains. *Int. J. Quantum Chem.* **1999**, *75*, 441–447. https://doi.org/10.1002/(SICI)1097-461X(1999)75:4/5<441::AID-QUA10>3.0.CO;2-B.
23. Limacher, P.A.; Mikkelsen, K.V.; Lüthi, H.P. On the Accurate Calculation of Polarizabilities and Second Hyperpolarizabilities of Polyacetylene Oligomer Chains Using the CAM-B3LYP Density Functional. *J. Chem. Phys.* **2009**, *130*, 194114. https://doi.org/10.1063/1.3139023.
24. Körzdörfer, T.; Parrish, R.M.; Sears, J.S.; Sherrill, C.D.; Brédas, J.L. On the Relationship Between Bond-Length Alternation and Many-Electron Self-Interaction Error. *J. Chem. Phys.* **2012**, *137*, 124305. https://doi.org/10.1063/1.4752431.
25. Jacquemin, D.; Champagne, B.; André, J.M. Electron Correlation Effects upon the Static (Hyper)polarizabilities of Push-Pull Conjugated Polyenes and Polyynes. *Int. J. Quantum Chem.* **1997**, *65*, 679–688. https://doi.org/10.1002/(SICI)1097-461X(1997)65:5<679::AID-QUA34>3.0.CO;2-V.
26. Jacquemin, D.; Perpète, E.A.; André, J.M. NLO Response of Polymethineimine and Polymethineimine/Polyacetylene Conformers: Assessment of Electron Correlation Effects. *Int. J. Quantum Chem.* **2005**, *105*, 553–563. https://doi.org/10.1002/qua.20649.
27. Jacquemin, D.; Laurent, A.D.; Perpète, E.A.; André, J.M. An Ab Initio Simulation of the UV/Visible Spectra of N-Benzylideneaniline Dyes. *Int. J. Quantum Chem.* **2009**, *109*, 3506–3515. https://doi.org/10.1002/qua.22303.
28. Patel, P.D.; Masunov, A.E. Time-Dependent Density Functional Theory Study of Structure-Property Relationships in Diarylethene Photochromic Compounds. In *Lecture Notes in Computer Science. Computational Science – ICCS 2009*; Springer, 2009; Vol. 5545, pp. 211–220.
29. Franco, L.R.; Brandão, I.; Fonseca, T.L.; Georg, H.C. Elucidating the Structure of Merocyanine Dyes with the ASEC-FEG Method. Phenol Blue in Solution. *J. Chem. Phys.* **2016**, *145*, 194301. https://doi.org/10.1063/1.4967290.
30. Janowska, I.; Zakrzewski, J.; Nakatani, K.; Palusiak, M.; Walak, M.; Scholl, H. Ferrocenyl D–π–A Conjugated Polyenes with 3-Dicyanomethylidene-1-indanone and 1,3-Bis(dicyanomethylidene)indane Acceptor Groups: Synthesis, Linear and Second-Order Nonlinear Optical Properties and Electrochemistry. *J. Organomet. Chem.* **2006**, *691*, 323–330. https://doi.org/10.1016/j.jorganchem.2005.08.033.
31. Alparone, A. Structural, Torsional, Vibrational and Response Electric Properties of 2,2′-Bitellurophene Rotamers. An Ab Initio and Density Functional Theory Investigation. *Struct. Chem.* **2014**, *25*, 959–968. https://doi.org/10.1007/s11224-013-0370-6.
32. Raptis, S.G.; Nasiou, S.M.; Demetropoulos, I.N.; Papadopoulos, M.G. Static and Frequency Dependent Polarizabilities and Hyperpolarizabilities of H_2Sn. *J. Comput. Chem.* **1998**, *19*, 1698–1715. https://doi.org/10.1002/(SICI)1096-987X(19981130)19:15<1698::AID-JCC3>3.0.CO;2-I.
33. Briquet, L.; Vercauteren, D.P.; André, J.M.; Perpète, E.A.; Jacquemin, D. On the Geometries and UV/Vis Spectra of Substituted trans-Azobenzenes. *Chem. Phys. Lett.* **2007**, *435*, 257–262. https://doi.org/10.1016/j.cplett.2006.12.065.
34. de Queiroz, T.B.; Kümmel, S. Charge-Transfer Excitations in Low-Gap Systems under the Influence of Solvation and Conformational Disorder: Exploring Range-Separation Tuning. *J. Chem. Phys.* **2014**, *141*, 084303. https://doi.org/10.1063/1.4892937.

35. Valverde, C.; Osório, F.A.P.; Fonseca, T.L.; Baseia, B. DFT Study of Third-Order Nonlinear Susceptibility of a Chalcone Crystal. *Chem. Phys. Lett.* **2018**, *706*, 170–174. https://doi.org/10.1016/j.cplett.2018.06.001.
36. Jacobsen, H.; Cavallo, L. Directions for Use of Density Functional Theory: A Short Instruction Manual for Chemists. In *Handbook of Computational Chemistry*; Springer International Publishing: Cham, 2017; pp. 225–267.
37. Lewars, E.G. *Computational Chemistry: Introduction to the Theory and Applications of Molecular and Quantum Mechanics*, 3 ed.; Springer International Publishing: Cham, 2016.
38. Govindarasu, K.; Kavitha, E. Molecular structure, vibrational spectra, NBO, UV and first order hyperpolarizability, analysis of 4-Chloro-DL-phenylalanine by density functional theory. *Spectrochim. Acta A Mol. Biomol. Spectrosc.* **2014**, *133*, 799–810. https://doi.org/10.1016/j.saa.2014.06.019.
39. Govindarasu, K.; Kavitha, E. Vibrational spectra, molecular structure, NBO, UV, NMR, first order hyperpolarizability, analysis of 4-Methoxy-4′-Nitrobiphenyl by density functional theory. *Spectrochim. Acta A Mol. Biomol. Spectrosc.* **2014**, *122*, 130–141. https://doi.org/10.1016/j.saa.2013.10.122.
40. Avramopoulos, A.; Reis, H.; Tzeli, D.; Zaleśny, R.; Papadopoulos, M.G. Photoswitchable Molecular Units with Tunable Nonlinear Optical Activity: A Theoretical Investigation. *Molecules* **2023**, *28*, 5646. https://doi.org/10.3390/molecules28155646.
41. Shu, C.; Jiang, Z.; Biczysko, M. Toward accurate prediction of amino acid derivatives structure and energetics from DFT: glycine conformers and their interconversions. *J. Mol. Model.* **2020**, *26*, 129. https://doi.org/10.1007/s00894-020-4342-7.
42. Marques, S.; Castro, M.A.; Pontes, R.B.; Leão, S.A.; Fonseca, T.L. 155. Second hyperpolarizabilities of alkali- and alkaline-earth-doped boron nitride nanotubes. *Chem. Phys. Lett.* **2023**, *821*, 140473. https://doi.org/10.1016/j.cplett.2023.140473.
43. Mondal, A.; Hatua, K.; Nandi, P.K. Static second hyperpolarizability of twisted ethylene: A comprehensive computational study. *J. Theor. Comput. Chem.* **2015**, *14*, 1550060. https://doi.org/10.1142/S0219633615500601.
44. Bulik, I.W.; Zaleśny, R.; Bartkowiak, W.; Luis, J.M.; Kirtman, B.; Scuseria, G.E.; Avramopoulos, A.; Reis, H.; Papadopoulos, M.G. Performance of density functional theory in computing nonresonant vibrational (hyper)polarizabilities. *J. Comput. Chem.* **2013**, *34*, 1775–1784. https://doi.org/10.1002/jcc.23316.
45. Sreedharan, R.; Ravi, S.; Raghi, K.R.; Kumar, T.K.M.; Naseema, K. Growth, linear- nonlinear optical studies and quantum chemistry formalism on an organic NLO crystal for opto-electronic applications: experimental and theoretical approach. *SN Appl. Sci.* **2020**, *2*, 1–18. https://doi.org/10.1007/s42452-020-2360-9.
46. Armaković, S.; Armaković, S.; Šetrajčić, J.; Holodkov, V. Aromaticity, response, and nonlinear optical properties of sumanene modified with boron and nitrogen atoms. *J. Mol. Model.* **2014**, *20*, 2538. https://doi.org/10.1007/s00894-014-2538-4.
47. Sheena Mary, Y.; Raju, K.; Panicker, C.Y.; Al-Saadi, A.A.; Thiemann, T. Molecular conformational analysis, vibrational spectra, NBO analysis and first hyperpolarizability of (2E)-3-(3-chlorophenyl)prop-2-enoic anhydride based on density functional theory calculations. *Spectrochim. Acta A Mol. Biomol. Spectrosc.* **2014**, *131*, 471–483. https://doi.org/10.1016/j.saa.2014.04.111.
48. Nithya, R.; Sowmiya, M.; Kolandaivel, P.; et al.. Structural, optical, and charge transport properties of cyclopentadithiophene derivatives: a theoretical study. *Struct. Chem.* **2014**, *25*, 715–731. https://doi.org/10.1007/s11224-013-0325-y.
49. Patil, D.S.; Avhad, K.C.; Kadam, M.M.; Sekar, N. Synthesis of red emitting triphenylamine derived NLOphoric D–π–A molecules: photophysical, and viscosity sensing studies. *SN Appl. Sci.* **2019**, *1*, 259. https://doi.org/10.1007/s42452-019-0268-z.
50. Shruthi, C.; Ravindrachary, V.; Guruswamy, B.; Prasad, D.J.; Goveas, J. Evaluation of structural, Hirshfeld surface and density functional theory of novel NLO thienyl chalcone single crystal. *SN Appl. Sci.* **2019**, *1*, 1697. https://doi.org/10.1007/s42452-019-1370-y.
51. Tafur, S.; Mikhailov, I.A.; Belfield, K.D.; Masunov, A.E. Predictions of Two Photon Absorption Profiles Using Time-Dependent Density Functional Theory Combined with SOS and CEO Formalisms. In Proceedings of the Computational Science – ICCS 2009; Allen, G.; Nabrzyski, J.; Seidel, E.; van Albada, G.D.; Dongarra, J.; Sloot, P.M.A., Eds., Berlin, Heidelberg, 2009; pp. 179–188. https://doi.org/10.1007/978-3-642-01973-9_20.
52. Issaoui, N.; Ghalla, H.; Muthu, S.; Flakus, H.T.; Oujia, B. Molecular structure, vibrational spectra, AIM, HOMO–LUMO, NBO, UV, first order hyperpolarizability, analysis of 3-thiophenecarboxylic acid monomer and dimer by Hartree–Fock and density functional theory. *Spectrochim. Acta A Mol. Biomol. Spectrosc.* **2015**, *136*, 1227–1242. https://doi.org/10.1016/j.saa.2014.10.008.
53. Jeyavijayan, S. Molecular structure, vibrational spectra, NBO analysis, first hyperpolarizability, and HOMO–LUMO studies of 2-amino-4-hydroxypyrimidine by density functional method. *J. Mol. Struct.* **2015**, *1085*, 137–146. https://doi.org/10.1016/j.molstruc.2014.12.047.
54. Karabacak, M.; Kurt, M.; Cinar, M.; Ayyappan, S.; Sudha, S.; Sundaraganesan, N. The spectroscopic (FT-IR, FT-Raman, UV) and first order hyperpolarizability, HOMO and LUMO analysis of 3-aminobenzophenone by density functional method. *Spectrochim. Acta A Mol. Biomol. Spectrosc.* **2012**, *92*, 365–376. https://doi.org/10.1016/j.saa.2012.02.067.
55. Krawczyk, P. Time-dependent density functional theory calculations of the solvatochromism of some azo sulfonamide fluorochromes. *J. Mol. Model.* **2015**, *21*, 118. https://doi.org/10.1007/s00894-015-2651-z.

56. Krishnakumar, V.; Sangeetha, R.; Mathammal, R.; Barathi, D. Density functional theory, comparative vibrational spectroscopic studies, HHOMO–LUMO, first hyperpolarizability analyses of 2-fluoro 5-nitrotoluene and 2-bromo 5-nitrotoluene. *Spectrochim. Acta A Mol. Biomol. Spectrosc.* **2013**, *104*, 77–86. https://doi.org/10.1016/j.saa.2012.10.002.
57. Li, Y.; Zhang, Y.; Qi, D.; Sun, C.; Yang, L. Nonlinear optical properties and performance optimization of the pro-aromatic chromophores for NLO materials. *J. Mater. Sci. Mater. Electron.* **2014**, *25*, 5255–5263. https://doi.org/10.1007/s10854-014-2298-z.
58. Ghiasi, R.; Gholipour, F. Borazine-based conjugated derivatives: Structural, electronic, and optical properties. *Russ. J. Phys. Chem. A* **2014**, *88*, 984–994. https://doi.org/10.1134/S0036024414060260.
59. Govindarasu, K.; Kavitha, E.; Sundaraganesan, N. Synthesis, structural, spectral (FTIR, FT-Raman, UV, NMR), NBO and first order hyperpolarizability analysis of *N*-phenylbenzenesulfonamide by density functional theory. *Spectrochim. Acta A Mol. Biomol. Spectrosc.* **2014**, *133*, 417–431. https://doi.org/10.1016/j.saa.2014.06.040.
60. Gümüs, H.P.; Ömer Tamer.; Avci, D.; Tarcan, E.; Atalay, Y. Theoretical investigations on nonlinear optical and spectroscopic properties of 6-(3,3,4,4,4-pentafluoro-2-hydroxy-1-butenyl)-2,4-pyrimidinedione: An efficient NLO material. *Russ. J. Phys. Chem. A* **2014**, *88*, 2348–2358. https://doi.org/10.1134/S0036024414130068.
61. Jonin, C.; Salmon, E.; Brevet, P.F. Hyper-Rayleigh scattering of adenine, thymine, and cytosine in neat water. *J. Chem. Phys.* **2021**, *155*, 204306. https://doi.org/10.1063/5.0069623.
62. Sarkar, A.; Das, M.; Bagchi, S. Electronic spectra and hyperpolarizabilities of structurally similar donor–acceptor dyes. A density functional theory analysis. *J. Mol. Struct.* **2015**, *1102*, 11–17. https://doi.org/10.1016/j.molstruc.2015.08.027.
63. Al-Zahrani, F.A.; Arshad, M.N.; Asiri, A.M.; Mahmood, T.; Gilani, M.A.; El-shishtawy, R.M. Synthesis and structural properties of 2-((10-alkyl-10H-phenothiazin-3-yl)methylene)malononitrile derivatives; a combined experimental and theoretical insight. *Chem. Cent. J.* **2016**, *10*, 13. https://doi.org/10.1186/s13065-016-0158-z.
64. Baillargeon, P.; Seidler, T.; Champagne, B.; et al.. Polar and helical isomorphous crystals of proline derivatives: Influence of a fluorine atom on the electric susceptibility. *Chem. Afr.* **2021**, *4*, 553–562. https://doi.org/10.1007/s42250-021-00236-w.
65. Balachandran, V.; Parimala, K. Tautomeric purine forms of 2-amino-6-chloropurine (N_9H_{10} and N_7H_{10}): Structures, vibrational assignments, NBO analysis, hyperpolarizability, HOMO–LUMO study using B3 based density functional calculations. *Spectrochim. Acta A Mol. Biomol. Spectrosc.* **2012**, *96*, 340–351. https://doi.org/10.1016/j.saa.2012.05.050.
66. Balachandran, V.; Parimala, K. Molecular structure, vibrational spectra, NBO analysis, first hyperpolarizability, and HOMO, LUMO studies of mesityl chloride by density functional methods. *J. Mol. Struct.* **2012**, *1007*, 136–145. https://doi.org/10.1016/j.molstruc.2011.10.035.
67. Beena, T.; Sudha, L.; Nataraj, A.; Balachandran, V.; Kannan, D.; Ponnuswamy, M.N. Synthesis, spectroscopic, dielectric, molecular docking and DFT studies of (3E)-3-(4-methylbenzylidene)-3,4-dihydro-2H-chromen-2-one: an anticancer agent. *Chem. Cent. J.* **2017**, *11*, 6. https://doi.org/10.1186/s13065-016-0230-8.
68. Sheena Mary, Y.; Raju, K.; Panicker, C.Y.; Al-Saadi, A.A.; Thiemann, T.; Van Alsenoy, C. Molecular conformational analysis, vibrational spectra, NBO analysis and first hyperpolarizability of (2E)-3-phenylprop-2-enoic anhydride based on density functional theory calculations. *Spectrochim. Acta A Mol. Biomol. Spectrosc.* **2014**, *128*, 638–646. https://doi.org/10.1016/j.saa.2014.02.194.
69. Hatua, K.; Nandi, P.K. Double coned inverse sandwich complexes M-(η^{n4}-C_4H_4)-M′ of Gr-IA and Gr-IIA metals: theoretical study of electronic structure and second hyperpolarizability. *J. Mol. Model.* **2014**, *20*, 2440. https://doi.org/10.1007/s00894-014-2440-0.
70. Zaleśny, R.; Medved′, M.; Sitkiewicz, S.P.; Matito, E.; Luis, J.M. Can Density Functional Theory Be Trusted for High-Order Electric Properties? The Case of Hydrogen-Bonded Complexes. *J. Chem. Theory Comput.* **2019**, *15*, 3570–3579. https://doi.org/10.1021/acs.jctc.9b00139.
71. Mishra, V.R.; Ghanavatkar, C.W.; Sharma, S.; Premarani, A.; Mathew, E.; Joe, I.H.; Sekar, N. Linear and NLO Properties of Functional Group and Position Isomers of Azo and Azomethine: Comparative Photophysical-Electrochemical Properties, Z-Scan and DFT Studies. *Chem. Select* **2020**, *5*, 10743–10753. https://doi.org/10.1002/slct.202001072.
72. Besalú-Sala, P.; Sitkiewicz, S.P.; Salvador, P.; Matito, E.; Luis, J.M. A new tuned range-separated density functional for the accurate calculation of second hyperpolarizabilities. *Phys. Chem. Chem. Phys.* **2020**, *22*, 11871–11880. https://doi.org/10.1039/D0CP01291B.
73. Agarwal, P.; Choudhary, N.; Gupta, A.; Tandon, P. Density functional theory studies on the structure, spectra (FT-IR, FT-Raman, and UV) and first order molecular hyperpolarizability of 2-hydroxy-3-methoxy-N-(2-chloro-benzyl)-benzaldehyde-imine: Comparison to experimental data. *Vibr. Spectrosc.* **2013**, *64*, 134–147. https://doi.org/10.1016/j.vibspec.2012.11.005.
74. Castet, F.; Lerychard, T.; Pielak, K.; Szalóki, G.; Dalinot, C.; Leriche, P.; Sanguinet, L.; Champagne, B.; Rodriguez, V. How Dimerization Through a Spiro Junction Modifies the Nonlinear Optical Properties of a Push–Pull Organic Dye: Insights from Theory and Hyper-Rayleigh Scattering. *ChemPhotoChem* **2017**, *1*, 93–101. https://doi.org/10.1002/cptc.201600039.
75. Jensen, L.; van Duijnen, P.T.; Snijders, J.G.; Chong, D.P. Time-dependent density functional study of the static second hyperpolarizability of BB-, NN- and BN-substituted C60. *Chem. Phys. Lett.* **2002**, *359*, 524–529. https://doi.org/10.1016/S0009-2614(02)00739-X.

76. Govindarasu, K.; Kavitha, E. Vibrational spectra, molecular structure, NBO, NMR, UV first order hyperpolarizability, analysis of (S)-(–)-N-(5-Nitro-2-pyridyl) alaninol by Density functional theory. *Spectrochim. Acta A Mol. Biomol. Spectrosc.* **2014**, *127*, 498–510. https://doi.org/10.1016/j.saa.2014.02.107.
77. Migalska-Zalas, A.; Korchi, K.E.; Chtouki, T. Enhanced nonlinear optical properties due to electronic delocalization in conjugated benzodifuran derivatives. *Opt. Quantum Electron.* **2018**, *50*, 389. https://doi.org/10.1007/s11082-018-1659-x.
78. Vishnumurthy, K.A.; Girish, K.H.; Adhikari, A.V. Synthesis, physicochemical properties and computational study of donor–acceptor polymer for optical limiting application. *SN Appl. Sci.* **2020**, *2*, 1727. https://doi.org/10.1007/s42452-020-03523-2.
79. Maity, R.; Mandal, D.; Misra, A. Role of π-electron conjugation in determining the electrical responsive properties of polychlorinated biphenyls: a DFT based computational study. *SN Appl. Sci.* **2020**, *2*, 418. https://doi.org/10.1007/s42452-020-2068-x.
80. Begam Elavarasi, S.; Mariam, D.; Ummal Momeen, M.; Hu, J.; Guin, M. Effect of fluorination on bandgap, first and second order hyperpolarizabilities in lithium substituted adamantane: A time dependent density functional theory. *Chem. Phys. Lett.* **2019**, *715*, 310–316. https://doi.org/10.1016/j.cplett.2018.11.034.
81. Mveme, C.D.D.; Tchangnwa Nya, F.; Ejuh, G.W.; Ndjaka, J.M.B. A density functional theory (DFT) study of the doping effect on 4-[2-(2-N, N-dihydroxy amino thiophene) vinyl] benzenamine. *SN Appl. Sci.* **2021**, *3*, 317. https://doi.org/10.1007/s42452-021-04277-1.
82. Krawczyk, P. Modulation of benzofuran structure as a fluorescent probe to optimize linear and nonlinear optical properties and biological activities. *J. Mol. Model.* **2020**, *26*, 272. https://doi.org/10.1007/s00894-020-04539-6.
83. Li, X.; Sun, S.L.; Ma, N.N.; Qiu, Y.Q.; Fu, Q. Quantum chemical studies on tuning the second-order nonlinear optical molecular switching of triarylborane derivatives. *Chin. Sci. Bull.* **2012**, *57*, 1772–1780. https://doi.org/10.1007/s11434-012-5059-5.
84. Mohbiya, D.R.; Sekar, N. Tuning 'Stokes Shift' and ICT Character by Varying the Donor Group in Imidazo[1,5a]pyridines: A Combined Optical, DFT, TD-DFT and NLO Approach. *Chem. Select* **2018**, *3*, 1635–1644. https://doi.org/10.1002/slct.201702579.
85. Woller, T.; Geerlings, P.; De Proft, F.; Champagne, B.; Alonso, M. Aromaticity as a Guiding Concept for Spectroscopic Features and Nonlinear Optical Properties of Porphyrinoids. *Molecules* **2018**, *23*, 1333. https://doi.org/10.3390/molecules23061333.
86. Valverde, C.; Vinhal, R.S.; Naves, L.F.N.; Custódio, J.M.F.; Baseia, B.; de Oliveira, H.C.B.; Perez, C.N.; Napolitano, H.B.; Osório, F.A.P. Remarkable Nonlinear Properties of a Novel Quinolidone Derivative: Joint Synthesis and Molecular Modeling. *Molecules* **2022**, *27*, 2379. https://doi.org/10.3390/molecules27082379.
87. Gong, P.; An, L.; Tong, J.; Liu, X.; Liang, Z.; Li, J. Design of A-D-A-Type Organic Third-Order Nonlinear Optical Materials Based on Benzodithiophene: A DFT Study. *Nanomaterials* **2022**, *12*, 3700. https://doi.org/10.3390/nano12203700.
88. Chadli, R.; Rabah, M.Z.; Khelladi, I.; Haddou, A.; Ameri, B.; Sakkal-Rahal, M. Synthesis, spectroscopic characterization techniques of aromatic iminoaniline derived compound, along with the evaluation of its LNO properties using quantum chemistry. *Sci. Rep.* **2024**, *14*, 27383. https://doi.org/10.1038/s41598-024-75117-6.
89. Zhang, C.C.; Xu, H.L.; Hu, Y.Y.; Sun, S.L.; Su, Z.M. Quantum Chemical Research on Structures, Linear and Nonlinear Optical Properties of the Li@n-Acenes Salt (n = 1, 2, 3, and 4). *J. Phys. Chem. A* **2011**, *115*, 2035–2040. https://doi.org/10.1021/jp110412n.
90. Cardenuto, M.H.; Champagne, B. The first hyperpolarizability of nitrobenzene in benzene solutions: investigation of the effects of electron correlation within the sequential QM/MM approach. *Phys. Chem. Chem. Phys.* **2015**, *17*, 23634–23642. https://doi.org/10.1039/C5CP03455H.
91. Torrent-Sucarrat, M.; Anglada, J.M.; Luis, J.M. Evaluation of the Nonlinear Optical Properties for Annulenes with Hückel and Möbius Topologies. *J. Chem. Theory Comput.* **2011**, *7*, 3935–3943. https://doi.org/10.1021/ct2005424.
92. Marques, S.; Castro, M.A.; Leão, S.A.; Fonseca, T.L. Electronic and Vibrational Hyperpolarizabilities of Lithium Substituted (Aza)benzenes and (Aza)naphthalenes. *J. Phys. Chem. A* **2018**, *122*, 7402–7412. https://doi.org/10.1021/acs.jpca.8b05612.
93. Mydlova, L.; Sahraoui, B.; El-Ghayoury, A.; Berdowski, J.; Migalska-Zalas, A.; Makowska-Janusik, M. Hierarchical Modeling of the Nonlinear Optical Response of Composite Materials Based on Tetrathiafulvalene Derivatives. *Molecules* **2024**, *29*, 3720. https://doi.org/10.3390/molecules29163720.
94. Kosar, N.; Kanwal, S.; Hamid, M.H.S.A.; Ayub, K.; Gilani, M.A.; Imran, M.; Arshad, M.; Alkhalifah, M.A.; Sheikh, N.S.; Mahmood, T. Role of Delocalization, Asymmetric Distribution of π-Electrons and Elongated Conjugation System for Enhancement of NLO Response of Open Form of Spiropyran-Based Thermochromes. *Molecules* **2023**, *28*, 6283. https://doi.org/10.3390/molecules28176283.
95. Karamanis, P.; Maroulis, G. An ab initio study of CX3-substitution (X = H, F, Cl, Br, I) effects on the static electric polarizability and hyperpolarizability of diacetylene. *J. Phys. Org. Chem.* **2011**, *24*, 588–599. https://doi.org/10.1002/poc.1797.
96. Jacquemin, D.; Perpète, E.A.; Ciofini, I.; Adamo, C. Assessment of recently developed density functional approaches for the evaluation of the bond length alternation in polyacetylene. *Chem. Phys. Lett.* **2005**, *405*, 376–381. https://doi.org/10.1016/j.cplett.2005.02.037.
97. Garza, A.J.; Osman, O.I.; Wazzan, N.A.; Khan, S.B.; Asiri, A.M.; Scuseria, G.E. A computational study of the nonlinear optical properties of carbazole derivatives: theory refines experiment. *Theor. Chem. Acc.* **2014**, *133*, 1458. https://doi.org/10.1007/s00214-014-1458-9.

98. Banerjee, P.; Nandi, P.K. Electronic structure and second hyperpolarizability of M(NA2)2 (M = Be, Mg, Ca; A = H, Li, Na) complexes. *Chem. Phys. Lett.* **2015**, *637*, 164–171. https://doi.org/10.1016/j.cplett.2015.08.008.
99. Pecul, M. Density functional and coupled cluster calculations of dynamic hyperpolarizabilities and their geometry derivatives. *Chem. Phys. Lett.* **2005**, *404*, 217–226. https://doi.org/10.1016/j.cplett.2005.01.057.
100. Nénon, S.; Champagne, B.; Spassova, M.I. Assessing Long-Range Corrected Functionals with Physically-Adjusted Range-Separated Parameters for Calculating the Polarizability and the Second Hyperpolarizability of Polydiacetylene and Polybutatriene Chains. *Phys. Chem. Chem. Phys.* **2014**, *16*, 7083–7088. https://doi.org/10.1039/C4CP00105B.
101. A. Zawada, A.K.K.; Bartkowiak, W. On the potential application of DFT methods in predicting the interaction-induced electric properties of molecular complexes. Molecular H-bonded chains as a case of study. *J. Mol. Model.* **2012**, *18*, 3073–3086. https://doi.org/10.1007/s00894-011-1312-0.
102. S. Bonness, H. Fukui, K.Y.R.K.B.C.E.B.; Nakano, M. Theoretical investigation on the second hyperpolarizabilities of open-shell singlet systems by spin-unrestricted density functional theory with long-range correction: Range separating parameter dependence. *Chem. Phys. Lett.* **2010**, *493*, 195–199. https://doi.org/10.1016/j.cplett.2010.05.026.
103. H. Fukui, Y. Inoue, R.K.Y.S.B.C.; Nakano, M. Tuned long-range corrected density functional theory method for evaluating the second hyperpolarizabilities of open-shell singlet metal–metal bonded systems. *Chem. Phys. Lett.* **2012**, *523*, 60–64. https://doi.org/10.1016/j.cplett.2011.12.033.
104. Yoneda, K.; Nakano, M.; Fukuda, K.; Matsui, H.; Takamuku, S.; Hirosaki, Y.; Kubo, T.; Kamada, K.; Champagne, B. Third-order nonlinear optical properties of one-dimensional open-shell molecular aggregates composed of phenalenyl radicals. *Chem. Eur. J.* **2014**, *20*, 11129–11136. https://doi.org/10.1002/chem.201402197.
105. R. Kishi, M. Dennis, K.F.Y.M.K.M.H.U.; Nakano, M. Theoretical Study on the Electronic Structure and Third-Order Nonlinear Optical Properties of Open-Shell Quinoidal Oligothiophenes. *J. Phys. Chem. C* **2013**, *117*, 21498–21508. https://doi.org/10.1021/jp407482h.
106. Qiu, Y.; Li, Z.H.; Ma, N.; Sun, S.; Zhang, M.; Liu, P.J. Third-order nonlinear optical properties of molecules containing aromatic diimides: effects of the aromatic core size and a redox-switchable modification. *J. Mol. Graph. Model.* **2013**, *41*, 79–88. https://doi.org/10.1016/j.jmgm.2013.01.008.
107. Haq, S.; Khalid, M.; Hussain, A.; Haroon, M.; Alshehri, S.M. A first principles based prediction of electronic and nonlinear optical properties towards cyclopenta thiophene chromophores with benzothiophene acceptor moieties. *Sci. Rep.* **2024**, *14*, 13971. https://doi.org/10.1038/s41598-024-64700-6.
108. Kaka, K.S.; Beaujean, P.; Castet, F.; Champagne, B. A quantum chemical investigation of the second hyperpolarizability of *p*-nitroaniline. *J. Chem. Phys.* **2023**, *159*, 114104. https://doi.org/10.1063/5.0164602.
109. M. Nakano, S. Yamada, S.K.; Yamaguchi, K. Hyperpolarizabilities of one-dimensional Hn systems: Second hyperpolarizability density analyses for regular and charged solitonlike linear chains. *Int. J. Quantum Chem.* **1998**, *70*, 269–282. https://doi.org/10.1002/(SICI)1097-461X(1998)70:2<269::AID-QUA3>3.0.CO;2-S.
110. D. Jacquemin, É. A. Perpète, G.E.S.I.C.; Adamo, C. Extensive TD-DFT investigation of the first electronic transition in substituted azobenzenes. *Chem. Phys. Lett.* **2008**, *465*, 226–229. https://doi.org/10.1016/j.cplett.2008.09.071.
111. Zakharov, A.B.; Ivanov, V.V.; Adamowicz, L., Optical Parameters of π-Conjugated Oligomer Chains from the Semiempirical Local Coupled-Cluster Theory. In *Practical Aspects of Computational Chemistry IV*; Springer US: Boston, MA, 2016; pp. 57–102. https://doi.org/10.1007/978-1-4899-7699-4_3.
112. Banerjee, P.; Avramopoulos, A.; Nandi, P.K. Static second-hyperpolarizability of diffuse electron cyclic compounds M_2A_2 (M = Be, Mg, Ca; A = Li, Na, K): Effect of basis set and electron correlation. *Chem. Phys. Lett.* **2019**, *729*, 92–98. https://doi.org/10.1016/j.cplett.2019.05.031.
113. Beerepoot, M.T.P.; Friese, D.H.; List, N.H.; Kongsted, J.; Ruud, K. Benchmarking two-photon absorption cross sections: Performance of CC2 and CAM-B3LYP. *Phys. Chem. Chem. Phys.* **2015**, *17*, 19306–19314. https://doi.org/10.1039/C5CP03241E.
114. Day, P.N.; Pachter, R.; Nguyen, K.A. Analysis of nonlinear optical properties in donor–acceptor materials. *J. Chem. Phys.* **2014**, *140*, 184308. https://doi.org/10.1063/1.4874267.
115. M. W. Jørgensen, R. Faber, A.L.; Sauer, S.P.A. Benchmarking Correlated Methods for Frequency-Dependent Polarizabilities: Aromatic Molecules with the CC3, CCSD, CC2, SOPPA, SOPPA(CC2), and SOPPA(CCSD) Methods. *J. Chem. Theory Comput.* **2020**, *16*, 3006–3018. https://doi.org/10.1021/acs.jctc.9b01300.
116. Beaujean, P.; Champagne, B. Coupled cluster evaluation of the frequency dispersion of the first and second hyperpolarizabilities of water, methanol, and dimethyl ether. *J. Chem. Phys.* **2016**, *145*, 044311. https://doi.org/10.1063/1.4958736.
117. Noga, J.; Pluta, T. Coupled cluster calculations of the electric properties of BeS. An analysis of the fifth-order non-iterative corrections. *Chem. Phys. Lett.* **1997**, *264*, 101–108. https://doi.org/10.1016/S0009-2614(96)01295-X.
118. Beaujean, P.; Champagne, B. Coupled cluster evaluation of the second and third harmonic scattering responses of small molecules. *Theor. Chem. Acc.* **2018**, *137*, 50. https://doi.org/10.1007/s00214-018-2219-y.

119. Tunega, D.; Noga, J. Static electric properties of LiH: explicitly correlated coupled cluster calculations. *Theor. Chem. Acc.* **1998**, *100*, 78–84. https://doi.org/10.1007/s002140050368.
120. Roos, B.O.; Sadlej, A.J. Polarized basis sets for accurate predictions of molecular electric properties. Electric moments of the LiH molecule. *Chem. Phys.* **1985**, *94*, 43–53. https://doi.org/10.1016/0301-0104(85)85064-3.
121. Sadlej, A.J. Medium-size polarized basis sets for high-level correlated calculations of molecular electric properties. *Collect. Czechoslov. Chem. Commun.* **1988**, *53*, 1995–2016. https://doi.org/10.1135/cccc19881995.
122. Sadlej, A.J. Medium-size polarized basis sets for high-level-correlated calculations of molecular electric properties. *Theor. Chim. Acta* **1991**, *79*, 123–140. https://doi.org/10.1007/BF01127101.
123. Aidas, K.; Angeli, C.; Bak, K.L.; Bakken, V.; Bast, R.; Boman, L.; Christiansen, O.; Cimiraglia, R.; Coriani, S.; Dahle, P.; et al. The Dalton Quantum Chemistry Program System. *WIREs Comput. Mol. Sci.* **2014**, *4*, 269–284. https://doi.org/10.1002/wcms.1172.
124. Dalton Project. *Dalton, a Molecular Electronic Structure Program, Release v2020.0.1*, 2020. Available at http://daltonprogram.org.
125. Foresman, J.B.; Frisch, E. Exploring Chemistry with Electronic Structure Methods, Gaussian, Inc., 3rd edn. *Exploring Chem. Electron. Struct. Methods* **2015**.
126. B. Champagne, E. Botek, M.N.T.N.; Yamaguchi, K. Basis set and electron correlation effects on the polarizability and second hyperpolarizability of model open-shell conjugated systems. *J. Chem. Phys.* **2005**, *122*, 114315. https://doi.org/10.1063/1.1880992.
127. Hatua, K.; Nandi, P.K. Beryllium-Cyclobutadiene Multidecker Inverse Sandwiches: Electronic Structure and Second-Hyperpolarizability. *J. Phys. Chem. A* **2013**, *117*, 12581–12589. https://doi.org/10.1021/jp407563f.
128. McLean, A.D.; Chandler, G.S. Contracted Gaussian basis sets for molecular calculations. I. Second row atoms, Z=11–18. *J. Chem. Phys.* **1980**, *72*, 5639–5648. https://doi.org/10.1063/1.438980.
129. R. Krishnan, J. S. Binkley, R.S.; Pople, J.A. Self-consistent molecular orbital methods. XX. A basis set for correlated wave functions. *J. Chem. Phys.* **1980**, *72*, 650–654. https://doi.org/10.1063/1.438955.
130. Clark, T.; Chandrasekhar, J.; Spitznagel, G.W.; von Ragué Schleyer, P. Efficient diffuse function-augmented basis sets for anion calculations. III. The 3-21+G basis set for first-row elements, Li–F. *J. Comput. Chem.* **1983**, *4*, 294–301. https://doi.org/10.1002/jcc.540040303.
131. Frisch, M.J.; Pople, J.A.; Binkley, J.S. Self-consistent molecular orbital methods 25. Supplementary functions for Gaussian basis sets. *J. Chem. Phys.* **1984**, *80*, 3265–3269. https://doi.org/10.1063/1.447079.
132. Kendall, R.A.; Thom H. Dunning, J.; Harrison, R.J. Electron affinities of the first-row atoms revisited. Systematic basis sets and wave functions. *J. Chem. Phys.* **1992**, *96*, 6796–6806. https://doi.org/10.1063/1.462569.
133. Davidson, E.R. Comment on "Comment on Dunning's correlation-consistent basis sets". *Chem. Phys. Lett.* **1996**, *260*, 514–518. https://doi.org/10.1016/0009-2614(96)00917-7.
134. Woon, D.E.; Dunning, T.H. Gaussian basis sets for use in correlated molecular calculations. III. The atoms aluminum through argon. *J. Chem. Phys.* **1993**, *98*, 1358–1371. https://doi.org/10.1063/1.464303.
135. Dunning, T.H. Gaussian basis sets for use in correlated molecular calculations. I. The atoms boron through neon and hydrogen. *J. Chem. Phys.* **1989**, *90*, 1007–1023. https://doi.org/10.1063/1.456153.
136. Pritchard, B.P.; Altarawy, D.; Didier, B.; Gibson, T.D.; Windus, T.L. New Basis Set Exchange: An Open, Up-to-Date Resource for the Molecular Sciences Community. *J. Chem. Inf. Model.* **2019**, *59*, 4814–4820. https://doi.org/10.1021/acs.jcim.9b00725.
137. Lebedev, V.I. Quadratures on the sphere. *USSR Comput. Math. Math. Phys.* **1976**, *16*, 10–24. https://doi.org/10.1016/0041-5553(76)90100-2.
138. Lebedev, V.I.; Skorokhodov, A.L. Quadrature formulas for a sphere of orders 41, 47 and 53. *Russ. Acad. Sci. Dokl. Math.* **1992**, *45*, 587–592.
139. Grimme, S. Semiempirical GGA-type density functional constructed with a long-range dispersion correction. *J. Comput. Chem.* **2006**, *27*, 1787–1799. https://doi.org/10.1002/jcc.20495.
140. Grimme, S.; Hansen, A.; Brandenburg, J.G.; Bannwarth, C. Dispersion-Corrected Mean-Field Electronic Structure Methods. *Chem. Rev.* **2016**, *116*, 5105–5154. https://doi.org/10.1021/acs.chemrev.5b00533.
141. Becke, A.D.; Johnson, E.R. A density-functional model of the dispersion interaction. *J. Chem. Phys.* **2005**, *123*, 154101. https://doi.org/10.1063/1.2065267.
142. Pettersen, E.F.; Goddard, T.D.; Huang, C.C.; Couch, G.S.; Greenblatt, D.M.; Meng, E.C.; Ferrin, T.E. UCSF Chimera–a visualization system for exploratory research and analysis. *J. Comput. Chem.* **2004**, *25*, 1605–1612. https://doi.org/10.1002/jcc.20084.
143. UCSF Chimera, Version 1.15 (build 42258), 2020. Resource for Biocomputing, Visualization, and Informatics at the University of California, San Francisco.
144. Chai, J.D.; Head-Gordon, M. Long-range corrected hybrid density functionals with damped atom–atom dispersion corrections. *Physical Chemistry Chemical Physics* **2008**, *10*, 6615. https://doi.org/10.1039/b810189b.
145. Olsen, J.M.H. Inclusion of dispersion corrections in the calculation of absorption spectra. https://gitlab.com/dalton/user-support/-/issues/92.

146. del Coso, R.; Solis, J. Relation between nonlinear refractive index and third-order susceptibility in absorbing media. *J. Opt. Soc. Am. B* **2004**, *21*, 640–644. https://doi.org/10.1364/JOSAB.21.000640.
147. Boyd, R.W.; Shi, Z.; De Leon, I. The third-order nonlinear optical susceptibility of gold. *Opt. Commun.* **2014**, *326*, 74–79. https://doi.org/10.1016/j.optcom.2014.03.005.
148. Sharafudeen, K.N.; Adithya, A.; Vijayakumar, S.; Sudheesh, P.; Kalluraya, B.; Chandrasekharan, K. Multiphoton absorption process and self-focusing effect in coumarin derivative doped PMMA films by z-scan and optical limiting studies. *Curr. Appl. Phys.* **2011**, *11*, 1089–1093. https://doi.org/10.1016/j.cap.2011.02.001.
149. Kim, S.; Chen, J.; Cheng, T.; Gindulyte, A.; He, J.; He, S.; Li, Q.; Shoemaker, B.A.; Thiessen, P.A.; Yu, B.; et al. PubChem in 2021: new data content and improved web interfaces. *Nucleic Acids Res.* **2021**, *49*, D1388–D1395. https://doi.org/10.1093/nar/gkaa971.
150. Willetts, A.; Rice, J.E.; Burland, D.M.; Shelton, D.P. Problems in the comparison of theoretical and experimental hyperpolarizabilities. *J. Chem. Phys.* **1992**, *97*, 7590–7599. https://doi.org/10.1063/1.463479.
151. Shi, R.F.; Garito, A.F. Introduction: Conventions and Standards for Nonlinear Optical Processes. In *Characterization Techniques and Tabulations for Organic Nonlinear Optical Materials*; Kuzyk, M.G.; Dirk, C.W., Eds.; Marcel Dekker: New York, 1998; pp. 1–36. https://doi.org/10.1201/9781315139036.
152. Jonsson, F. Lecture Notes on Nonlinear Optics, 2003. See http://jonsson.eu/research/lectures.
153. Butcher, P.N.; Cotter, D. *The Elements of Nonlinear Optics*; Cambridge Univ. Press: Cambridge, 1990. https://doi.org/10.1017/CBO9781139167994.
154. Theil, H. A Rank-Invariant Method of Linear and Polynomial Regression Analysis. In *Henri Theil's Contributions to Economics and Econometrics: Econometric Theory and Methodology*; Raj, B.; Koerts, J., Eds.; Springer Netherlands: Dordrecht, 1992; pp. 345–381. https://doi.org/10.1007/978-94-011-2546-8_20.
155. Sen, P.K. Estimates of the Regression Coefficient Based on Kendall's Tau. *J. Am. Stat. Assoc.* **1968**, *63*, 1379–1389. https://doi.org/10.1080/01621459.1968.10480934.
156. Borowski, M.; Fried, R. Online signal extraction by robust regression in moving windows with data-adaptive width selection. *Statistics and Computing* **2014**, *24*, 597–613. https://doi.org/10.1007/s11222-013-9391-7.
157. Politi, M.T.; Ferreira, J.C.; Patino, C.M. Nonparametric statistical tests: friend or foe? *J. Bras. Pneumol.* **2021**, *47*, e20210292. https://doi.org/10.36416/1806-3756/e20210292.
158. Mead, R.; Gilmour, S.G.; Mead, A. *Statistical Principles for the Design of Experiments*; Cambridge Univ. Press: Cambridge, 2012. https://doi.org/10.1017/CBO9781139020879.
159. Marenich, A.V.; Cramer, C.J.; Truhlar, D.G.; Guido, C.A.; Mennucci, B.; Scalmani, G.; Frisch, M.J. Practical computation of electronic excitation in solution: vertical excitation model. *Chem. Sci.* **2011**, *2*, 2143–2161. https://doi.org/10.1039/C1SC00313E.

Disclaimer/Publisher's Note: The statements, opinions and data contained in all publications are solely those of the individual author(s) and contributor(s) and not of MDPI and/or the editor(s). MDPI and/or the editor(s) disclaim responsibility for any injury to people or property resulting from any ideas, methods, instructions or products referred to in the content.
