# Supplementary figures and images for: Use of Factorial Design for Calculation of Second Hyperpolarizabilities
Source: Nanomaterials (Basel). 2025 Aug 23;15(17):1302. doi: 10.3390/nano15171302 (PMC12430289; doi:10.3390/nano15171302)

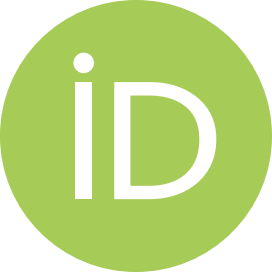

Supplement: Supplementary file 1 [file nanomaterials-15-01302-s001.zip › Definitions/logo-orcid.pdf]

# Average computation time per xc-Hamiltonian in CPKS+FF calculations

● Vacuum ● C-PCM

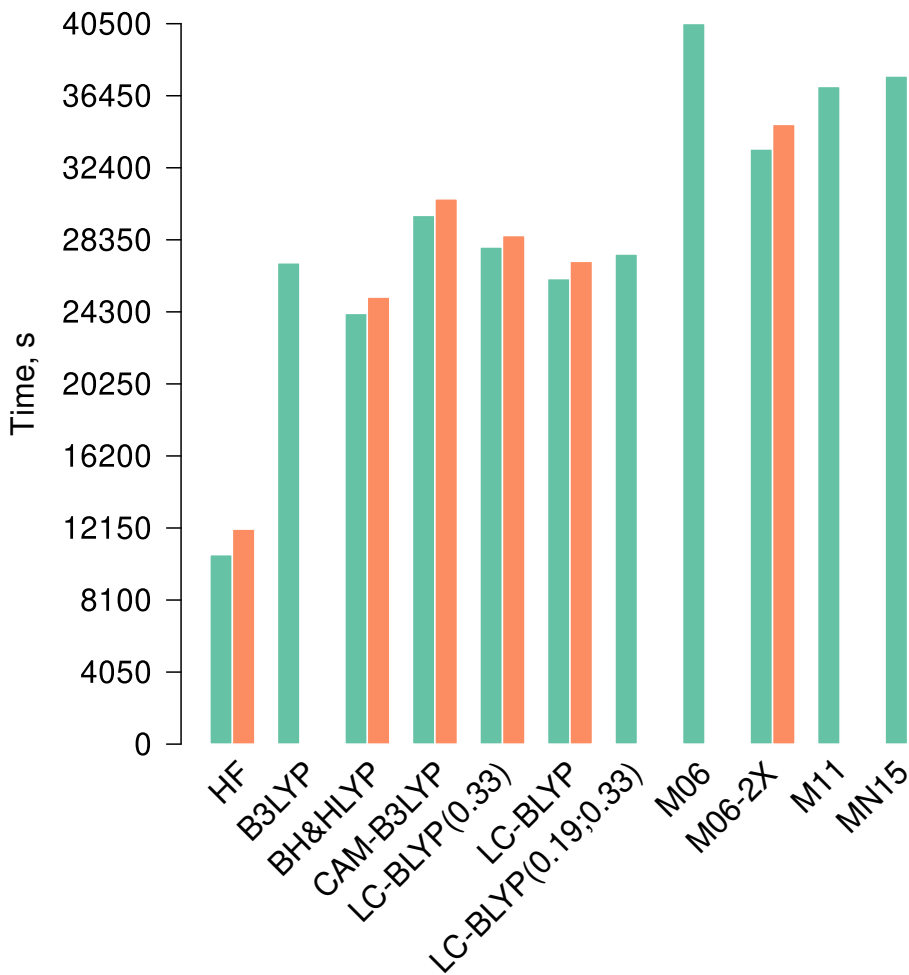

Supplement: Supplementary file 1 [file nanomaterials-15-01302-s001.zip › figs/FigS2.pdf]

# Average computation time per basis set in Response equation calculations

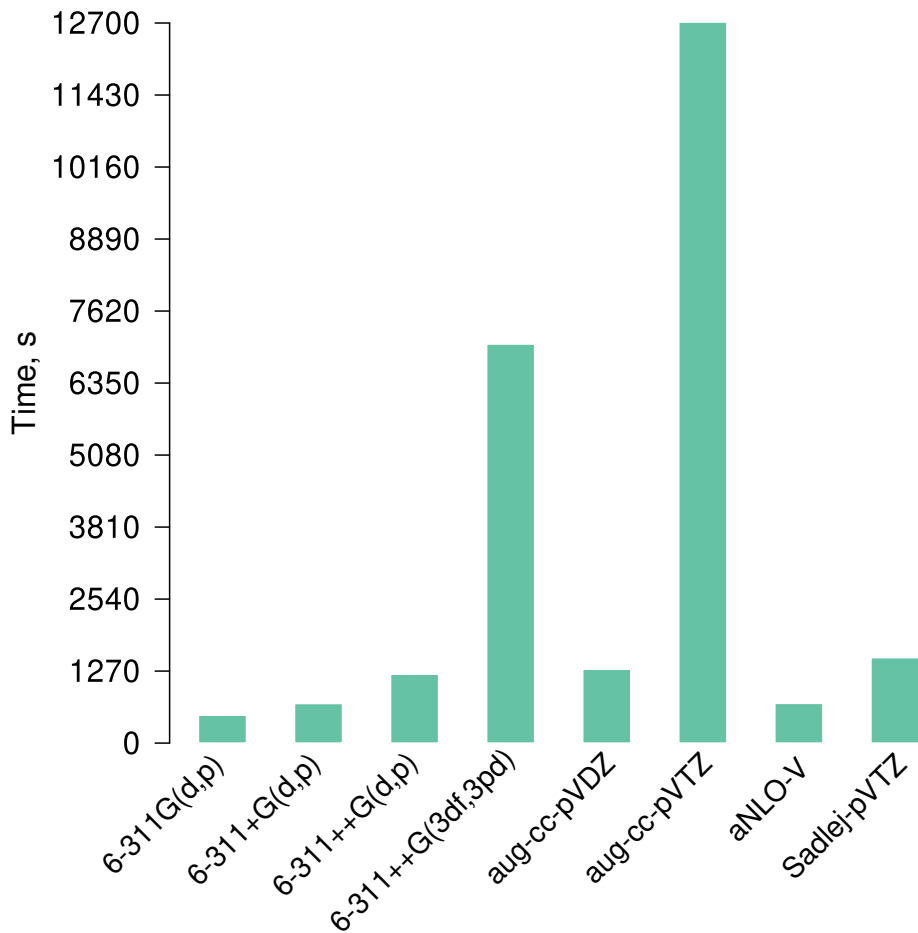

Supplement: Supplementary file 1 [file nanomaterials-15-01302-s001.zip › figs/FigS3.pdf]

ACS

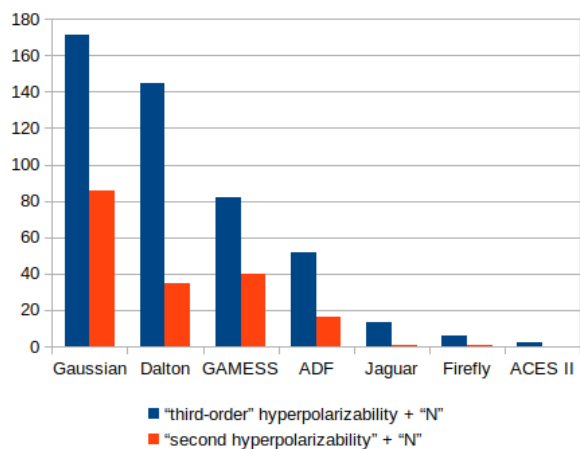

RSC

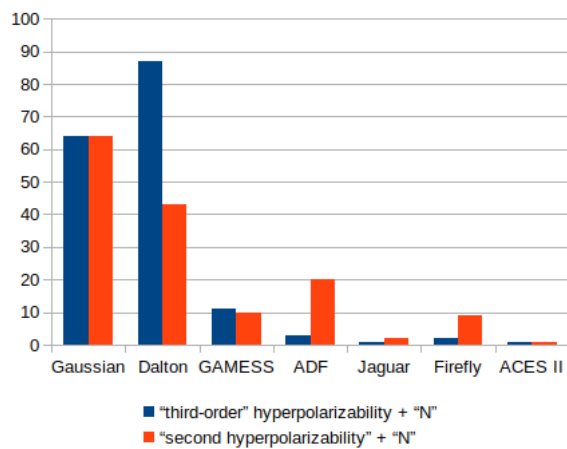

ScienceDirect

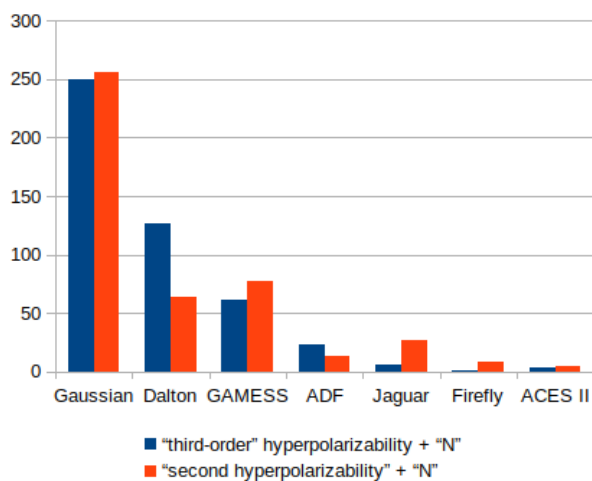

Scitation

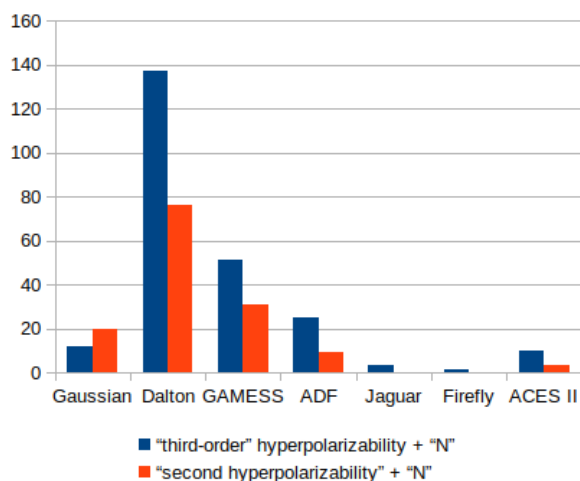

Springer

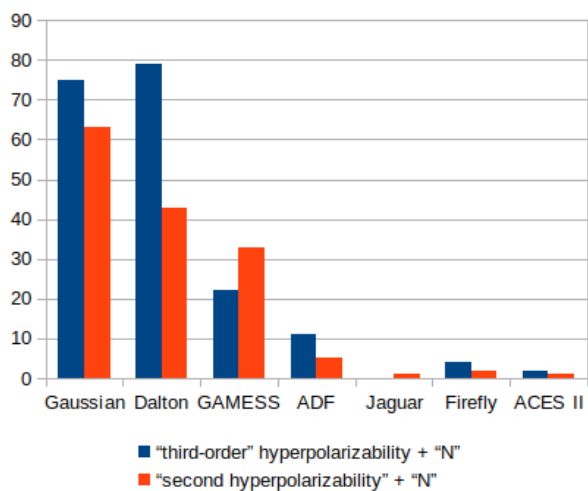

Wiley

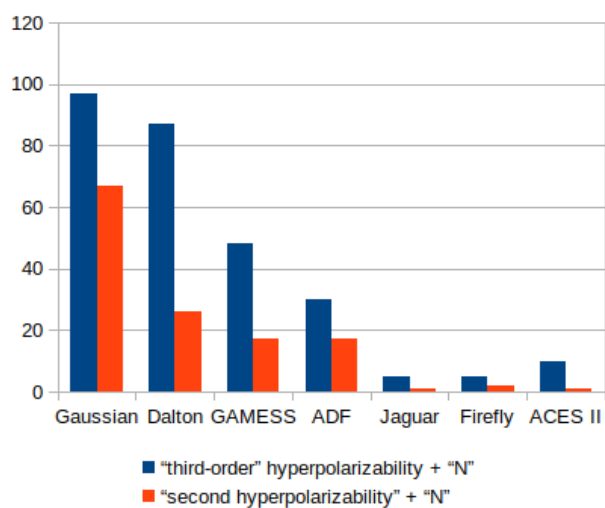

Supplement: Supplementary file 1 [file nanomaterials-15-01302-s001.zip › figs/FigS4.pdf]

### Nitrobenzene

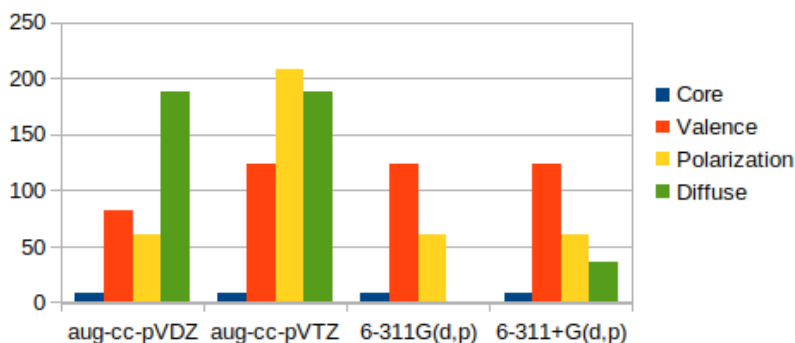

### Nitrobenzene

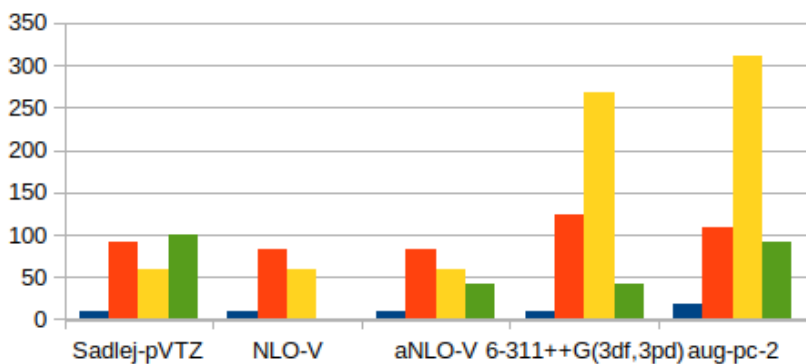

### Chloroform

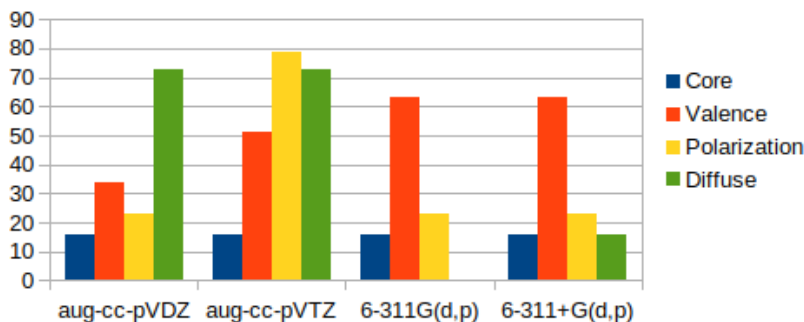

### Chloroform

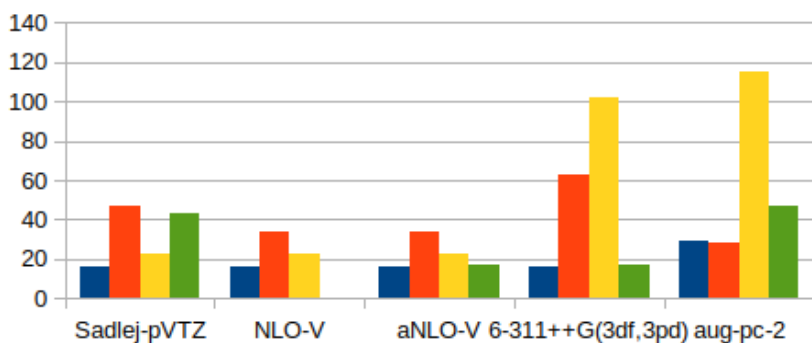

Supplement: Supplementary file 1 [file nanomaterials-15-01302-s001.zip › figs/FigS6.pdf]
